# Spatial and functional separation of mTORC1 signalling in response to different amino acid sources

**DOI:** 10.1038/s41556-024-01523-7

**Published:** 2024-10-09

**Authors:** Stephanie A. Fernandes, Danai-Dimitra Angelidaki, Julian Nüchel, Jiyoung Pan, Peter Gollwitzer, Yoav Elkis, Filippo Artoni, Sabine Wilhelm, Marija Kovacevic-Sarmiento, Constantinos Demetriades

**Affiliations:** 1 Max Planck Institute for Biology of Ageing, Cologne, Germany; 2 Cologne Graduate School of Ageing Research, Cologne, Germany; 3 Center for Biochemistry, Medical Faculty, University of Cologne, Cologne, Germany; 4 Cologne Excellence Cluster on Cellular Stress Responses in Aging-Associated Diseases, University of Cologne, Cologne, Germany

**Keywords:** TOR signalling, Nutrient signalling

## Abstract

Amino acid (AA) availability is a robust determinant of cell growth through controlling mechanistic/mammalian target of rapamycin complex 1 (mTORC1) activity. According to the predominant model in the field, AA sufficiency drives the recruitment and activation of mTORC1 on the lysosomal surface by the heterodimeric Rag GTPases, from where it coordinates the majority of cellular processes. Importantly, however, the teleonomy of the proposed lysosomal regulation of mTORC1 and where mTORC1 acts on its effector proteins remain enigmatic. Here, by using multiple pharmacological and genetic means to perturb the lysosomal AA-sensing and protein recycling machineries, we describe the spatial separation of mTORC1 regulation and downstream functions in mammalian cells, with lysosomal and non-lysosomal mTORC1 phosphorylating distinct substrates in response to different AA sources. Moreover, we reveal that a fraction of mTOR localizes at lysosomes owing to basal lysosomal proteolysis that locally supplies new AAs, even in cells grown in the presence of extracellular nutrients, whereas cytoplasmic mTORC1 is regulated by exogenous AAs. Overall, our study substantially expands our knowledge about the topology of mTORC1 regulation by AAs and hints at the existence of distinct, Rag- and lysosome-independent mechanisms that control its activity at other subcellular locations. Given the importance of mTORC1 signalling and AA sensing for human ageing and disease, our findings will probably pave the way towards the identification of function-specific mTORC1 regulators and thus highlight more effective targets for drug discovery against conditions with dysregulated mTORC1 activity in the future.

## Abstract (web summary)

Fernandes, Angelidaki et al. provide evidence supporting the spatial separation of mTORC1 activation and signalling. Differentially localized mTORC1 complexes phosphorylate distinct substrates in response to different amino acid supplies.

## Main

Cell growth is a crucial and tightly regulated process. Cells take up nutrients, such as amino acids (AAs), from their environment and use them to synthesize complex macromolecules, which they incorporate to increase their mass and grow. As growth is very energy consuming, cells have developed mechanisms to sense nutrient availability and to adjust their metabolism accordingly, so that they only grow when conditions are optimal. These mechanisms are of great importance, as dysregulation of growth can be detrimental for cellular and organismal health^1–3^.

The mechanistic/mammalian target of rapamycin complex 1 (mTORC1) is a master regulator of cellular growth and metabolism. It functions as a sensor and a molecular rheostat that links information from the cellular milieu to the physiological and metabolic properties of the cells^1,4–8^. The availability of AAs, in particular, is one of the most powerful signals for mTORC1 activation. In fact, AA signalling can override other stimuli such as growth factor availability^9–12^. As AAs are the building blocks to make proteins and mTORC1 controls protein synthesis, this mechanism ensures that cells produce proteins only when AAs are available. In addition to protein synthesis, mTORC1 activity affects the majority of cellular functions and, as a result, it can influence organismal health, lifespan and ageing^13^. Hyperactivation of mTORC1—caused mainly by mutations in its upstream regulators—is of clinical relevance and a common feature of most cancer types and several metabolic disorders^5,14–16^. Moreover, AA signalling to mTORC1 is medically relevant too, as nutritional AA overload has been linked to obesity and diabetes via hyperactivation of mTORC1 (ref. ^17^). Therefore, how AAs regulate mTORC1 is a fundamental biological question, relevant for both basic and clinical research.

Work over the last 15 years has built a lysosome-centric model of mTORC1 regulation by AAs, based on which AA availability controls the subcellular localization of mTORC1 via regulating the activity of the lysosomal heterodimeric Rag GTPases, composed of RagA or RagB bound to RagC or RagD. Under conditions of AA sufficiency, an ‘active’ Rag dimer (GTP-bound RagA/B and GDP-bound RagC/D) recruits mTORC1 to the lysosomal surface, where it is activated by another small GTPase called Rheb^18–21^. In contrast, AA starvation leads to ‘inactivation’ of the Rag heterodimer (GDP-bound RagA/B and GTP-bound RagC/D) and the subsequent release of mTORC1 from the lysosomal surface^18,22,23^. Following the original discovery of the Rag GTPases as a central hub in AA sensing^18,19^, the quest to understand how cells sense the availability of AAs over the following years has led to the identification of a large number of lysosomal and cytoplasmic proteins that modify Rag dimer activity in an AA-dependent manner (reviewed in refs. ^13,24,25^). According to this model, mTORC1 is thought to be activated by AAs exclusively on the lysosomal surface, from where it somehow controls all cellular processes^20,22^.

Interestingly, scattered evidence in the literature suggest that the cell biology of AA signalling to mTORC1 likely is more complex than currently appreciated^26^. Indeed, although a fraction of mTORC1 does localize to lysosomes in cells grown under AA-replete conditions, a substantial amount of this complex is found away from lysosomes^27,28^. Whether this non-lysosomal mTORC1 pool is active or inactive remains an important open question. Furthermore, many of the canonical mTORC1 effectors are known to be non-lysosomal proteins, hence where and how mTORC1 acts on its various substrates is, so far, unclear. Finally, because mTORC1 activity (as assessed by the phosphorylation of its most commonly used target, S6K) acutely responds to changes in exogenous AA sufficiency, the teleonomy of the exclusively lysosomal regulation of mTORC1 by AAs remains enigmatic. Taken together, a model where mTORC1 is exclusively present and regulated on the lysosomal surface appears counterintuitive. Here, using a number of pharmacological and genetic tools to perturb lysosomal function or the lysosomal tethering of mTORC1, we show that non-lysosomal mTORC1 can be active when exogenous AAs are present. Also, our data indicate that basal lysosomal proteolysis is required for the recruitment of mTORC1 to lysosomes and the activation of lysosomal mTORC1 signalling, even in the presence of exogenous AAs. Overall, our findings suggest the spatial separation of mTORC1 activities in cells, with lysosomal and non-lysosomal complexes regulating different cellular functions through the phosphorylation of distinct targets at each subcellular location.

## Results

### Lysosomal mTORC1 signalling requires intact lysosome function

**Fig. 1:**
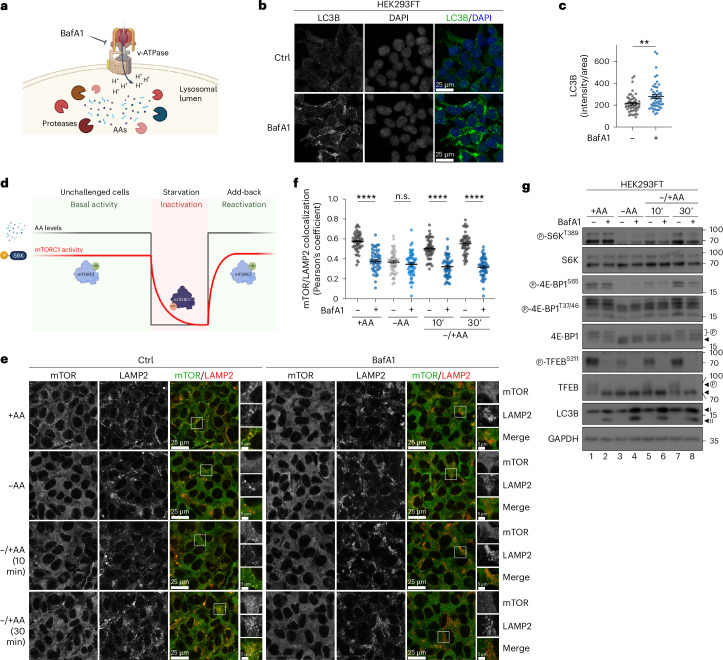
Blockage of lysosomal function disconnects mTORC1 localization and substrate-specific activity. **a**, A schematic model of the pharmacological inhibition of lysosomal function by BafA1 targeting the v-ATPase. **b**,**c**, Basal lysosomal proteolysis in HEK293FT cells shown by accumulation of LC3B upon BafA1 treatment (100 nM, 6 h before fixation) (**b**) and quantification of LC3B signal (**c**). *n*_Ctrl_ = 49 and *n*_BafA1_ = 50 individual cells from five independent fields per condition. **d**, A schematic representation of the treatment strategy followed in this study, assessing mTORC1 activity under basal (unchallenged cells), starvation or acute re-activation (AA add-back) conditions. AA levels are shown by a black line, and mTORC1 activity by a red line (see also Methods). **e**,**f**, Colocalization analysis of mTOR with LAMP2 (lysosomal marker) in HEK293FT WT cells, treated as indicated, using confocal microscopy (magnified insets shown on the right; scale bars, 25 μm and for insets, 5 μm) (**e**) and quantification of colocalization (**f**). *n* = 50 individual cells from five independent fields per condition. **g**, Immunoblots with lysates from HEK293FT WT cells treated with media containing or lacking AAs, in basal (+AA), starvation (−AA) or add-back (–/+AA) conditions, and BafA1 as shown, probed with the indicated antibodies. Arrowheads indicate bands corresponding to different protein forms when multiple bands are present. P, phosphorylated form. For **e**–**g**, BafA1 (100 nM) (or DMSO as control, Ctrl) was added directly in the media for 6 h before fixation (**e** and **f**) or lysis (**g**). For basal (+AA) conditions, culture media were replaced by +AA treatment media 90 min before fixation or lysis. For AA starvation (−AA), culture media were replaced by starvation media 1 h before fixation or lysis. For AA add-back samples (–/+AA), cells were first starved as described above and then starvation media were replaced by +AA treatment media for 10 or 30 min. BafA1 (or DMSO) was also included in the treatment media. The composition of all media is described in Methods. Data in graphs shown as mean ± s.e.m. **P < 0.01, ****P < 0.0001. Source numerical data and unprocessed blots are available in Source data. See also Extended Data Figs. 1 and 2. Source data.

**Extended Data Fig. 1:**
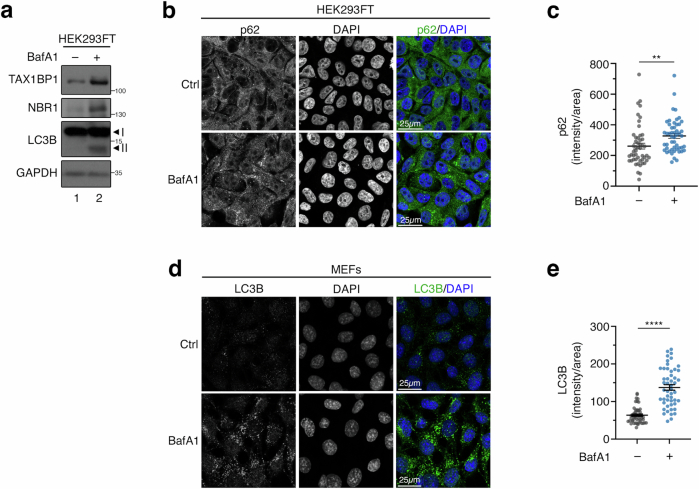
Basal lysosomal degradative activity takes place also in cells grown in the presence of exogenous nutrients. **(a)** Immunoblots with lysates from HEK293FT WT cells, treated with BafA1 (100 nM, 6 h) as shown, probed with the indicated antibodies. n = 3 independent experiments. **(b-c)** p62 accumulates in BafA1-treated cells (100 nM, 6 h), shown by immunofluorescence and confocal microscopy. Scale bars = 25 μm (b). Quantification of p62 signal from n = 50 individual cells from 5 independent fields per condition in (c). Representative data from one out of two independent replicate experiments are shown. **(d-e)** Basal lysosomal degradative activity in MEFs indicated by accumulation of LC3B upon BafA1 treatment (100 nM, 6 h). Scale bars = 25 μm (d). Quantification of LC3B signal from n = 50 individual cells from 5 independent fields per condition in (e). Representative data from one out of three independent replicate experiments are shown. Data in graphs shown as mean ± SEM. ** p < 0.01, **** p < 0.0001. Source numerical data and unprocessed blots are available in source data. Source data

Previous efforts to study how AAs signal to regulate mTORC1 have focused on the lysosomally anchored Rag GTPases and revealed a complex network of proteins that regulate Rag activity and function^13,24,25^. However, the reason for the lysosomal localization of mTORC1 in cells grown in the presence of exogenous AAs has been a long-standing question in the field. Given that lysosomes are the main degradative organelles in cells, we intuitively hypothesized that local AA production, due to basal protein recycling inside lysosomes, may be playing an important role in this process. Bafilomycin A1 (BafA1) is a macrolide antibiotic that blocks lysosomal function by targeting the v-ATPase (vacuolar H^+^ ATPase), causing alkalinization of the lysosomal lumen and preventing protease activity, AA efflux and autolysosome formation^20,29^ (Fig. 1a). Consistent with previous reports^30^, treatment of human embryonic kidney HEK293FT cells with BafA1 caused strong accumulation of LC3B (Fig. 1b,c and Extended Data Fig. 1a) and increased the levels of the macro- and micro-autophagy adaptor proteins TAX1BP1, NBR1 (Extended Data Fig. 1a) and p62 (Extended Data Fig. 1b,c), confirming that basal lysosomal proteolysis is robustly active in these cells, even when they are grown in the presence of exogenous AAs. Similar results were obtained in mouse embryonic fibroblasts (MEFs) (Extended Data Fig. 1d,e), showing that this phenomenon is not cell type or species specific.

**Fig. 2:**
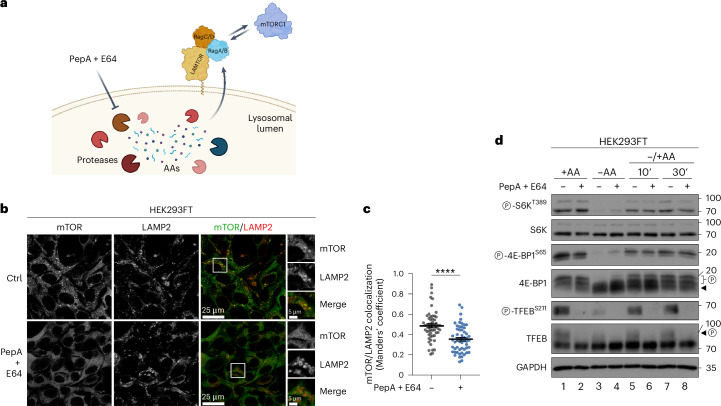
Blockage of lysosomal protease activity disconnects mTORC1 localization on lysosomes and activity towards cytoplasmic substrates. **a**, A schematic model of the pharmacological inhibition of lysosomal proteases by PepA and E64 blocking local AA production. **b**,**c**, Colocalization analysis of mTOR with LAMP2 (lysosomal marker) in HEK293FT WT cells (**b**) and its quantification (**c**), treated as indicated, using confocal microscopy. PepA (50 μM) and E64 (25 μM) (or DMSO as control, Ctrl) were added directly in the media for 16 h before fixation (magnified insets shown to the right; scale bars, 25 μm and for insets, 5 μm). *n* = 56 individual cells from three independent fields per condition. Data shown as mean ± s.e.m. ****P < 0.0001. **d**, Immunoblots with lysates from HEK293FT WT cells, treated with media containing or lacking AAs, in basal (+AA), starvation (−AA) or add-back (–/+AA) conditions, and protease inhibitors (PepA + E64) as shown, probed with the indicated antibodies. PepA (50 μM) and E64 (25 μM) were added directly in the media for 16 h before lysis. For basal (+AA) conditions, culture media were replaced by +AA treatment media 90 min before fixation or lysis. For AA starvation (−AA), culture media were replaced by starvation media 1 h before lysis. For AA add-back samples (–/+AA), cells were first starved as described above and then starvation media were replaced by +AA treatment media for 10 or 30 min. PepA + E64 (or DMSO) were also included in the treatment media. The composition of all media is described in Methods. Arrowheads indicate bands corresponding to different protein forms when multiple bands are present. P, phosphorylated form. Source numerical data and unprocessed blots are available in Source data. Source data

**Fig. 3:**
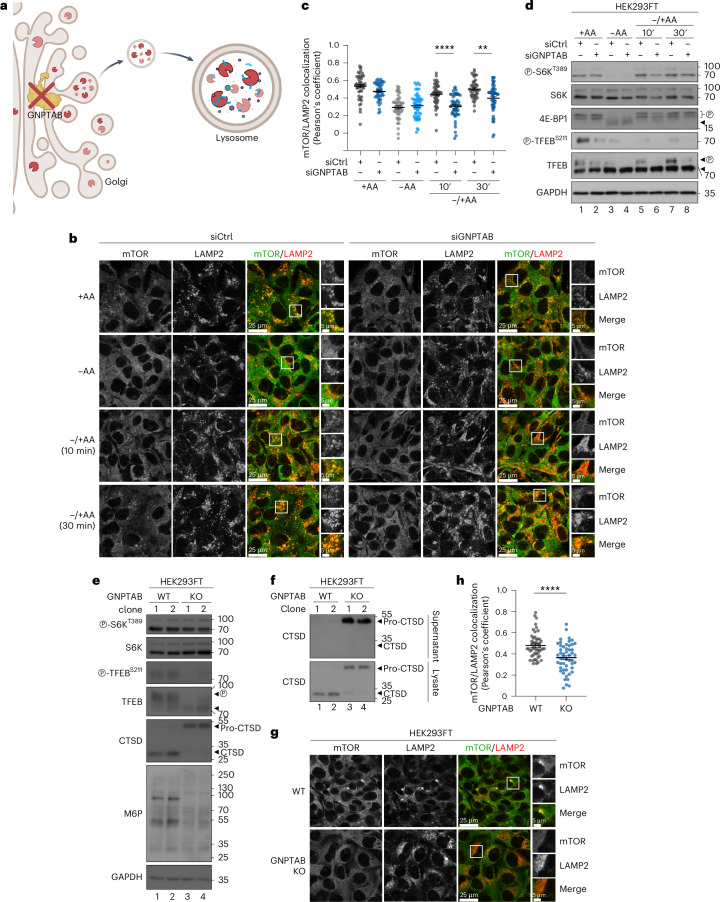
Blockage of proper lysosomal enzyme sorting and delivery disconnects mTORC1 localization on lysosomes and activity towards cytoplasmic substrates. **a**, A schematic model of lysosomal enzyme sorting at the Golgi and delivery to lysosomes that depends on the GNPTAB enzyme. **b**,**c**, Colocalization analysis of mTOR with LAMP2 (lysosomal marker) in HEK293FT WT cells using confocal microscopy (**b**) and its quantification (**c**). Cells were transiently transfected with siRNAs targeting *GNPTAB* or a control RNAi duplex (siCtrl) and treated as indicated. For basal (+AA) conditions, culture media were replaced by +AA treatment media 90 min before fixation. For AA starvation (−AA), culture media were replaced by starvation media 1 h before fixation. For AA add-back samples (–/+AA), cells were first starved as described above and then starvation media were replaced by +AA treatment media for 10 or 30 min. The composition of all media is described in Methods. *n* = 44–50 individual cells from five independent fields per condition (see also Methods). **d**, Immunoblots with lysates from HEK293FT WT cells transiently transfected with siRNAs targeting *GNPTAB* or a control RNAi duplex (siCtrl) and treated with media containing or lacking AAs, in basal (+AA), starvation (−AA) or add-back (–/+AA) conditions as described in **b**, probed with the indicated antibodies. **e**,**f**, Functional characterization of GNPTAB KO HEK293FT cells. Two independent KO clones show impaired cathepsin D (CTSD) processing and mannose-6-phosphate (M6P)-tagging of proteins. Note also the differential effect of GNPTAB loss on the different mTORC1 substrates (TFEB de-phosphorylated in KO cells, whereas S6K phosphorylation is largely unaffected) (**e**). The pro-form of CTSD is aberrantly secreted in the medium of GNPTAB KO cells (**f**). **g**,**h**, Lysosomal accumulations of mTOR are lost in GNPTAB KOs (**g**) and quantification of mTOR/LAMP2 colocalization (**h**). *n* = 50 individual cells from five independent fields per condition. For microscopy, magnified insets are shown to the right. Scale bars, 25 μm and for insets, 5 μm. Arrowheads indicate bands corresponding to different protein forms when multiple bands are present. P, phosphorylated form. Data in graphs shown as mean ± s.e.m. **P < 0.01, ****P < 0.0001. Source numerical data and unprocessed blots are available in Source data. Source data

**Fig. 4:**
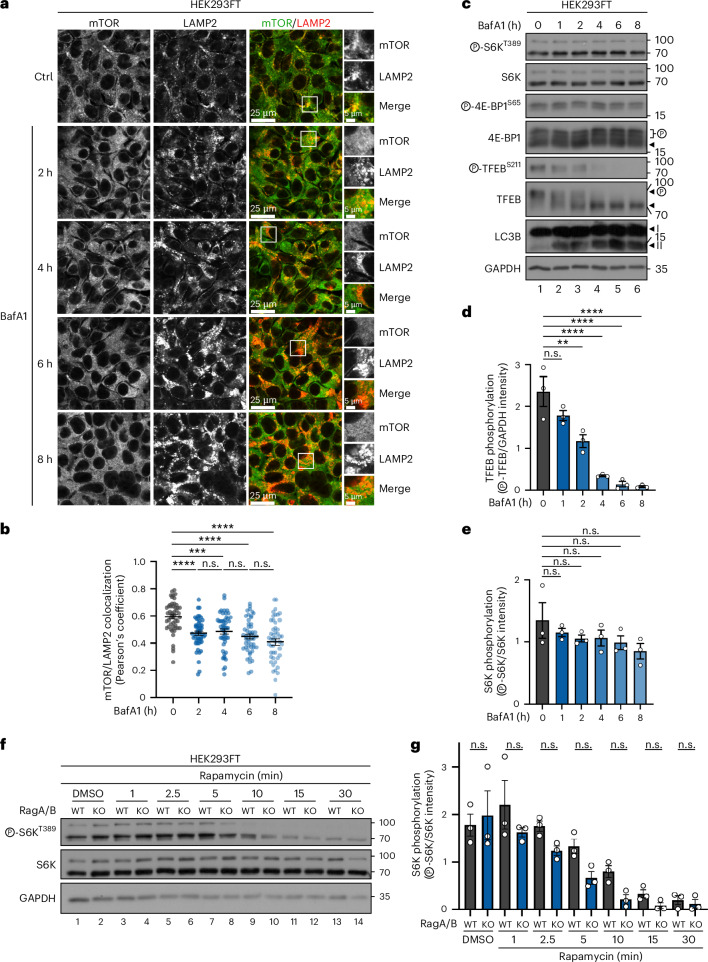
The persistent phosphorylation of cytoplasmic mTORC1 substrates in cells with non-lysosomal mTOR is not due to impaired or slower substrate dephosphorylation. **a**,**b**, mTOR/LAMP2 colocalization (**a**) and its quantification (**b**). mTOR delocalizes away from lysosomes already after 2 h of BafA1 treatment. Time course of BafA1 treatment (100 nM, 2–8 h) to block lysosomal function in HEK293FT cells. Magnified insets shown to the right (**a**). Scale bars, 25 μm and insets, 5 μm. *n* = 49–50 individual cells from five independent fields per condition (see also Methods). **c**–**e**, Dephosphorylation kinetics of lysosomal (TFEB) and cytoplasmic (S6K and 4E-BP1) substrates of mTORC1 upon BafA1 treatment (100 nM, 1–8 h) in HEK293FT cells showing a rapid drop in TFEB phosphorylation, whereas that of S6K/4E-BP1 remains largely unaffected even at much later timepoints (**c**). Quantification of TFEB phosphorylation in (**d**) and S6K phosphorylation in (**e**). **f**,**g**, The rapamycin time course (20 nM, 1–30 min) in control (WT) and RagA/B KO cells, assessing S6K dephosphorylation kinetics (**f**) and the quantification of S6K phosphorylation (**g**). The rate of S6K dephosphorylation is similar between Rag-proficient and Rag-deficient cells. Arrowheads indicate bands corresponding to different protein forms when multiple bands are present. P, phosphorylated form. Data in graphs shown as mean ± s.e.m. **P < 0.01, ***P < 0.001, ****P < 0.0001. n.s., non-significant. Source numerical data and unprocessed blots are available in Source data. Source data

**Extended Data Fig. 2:**
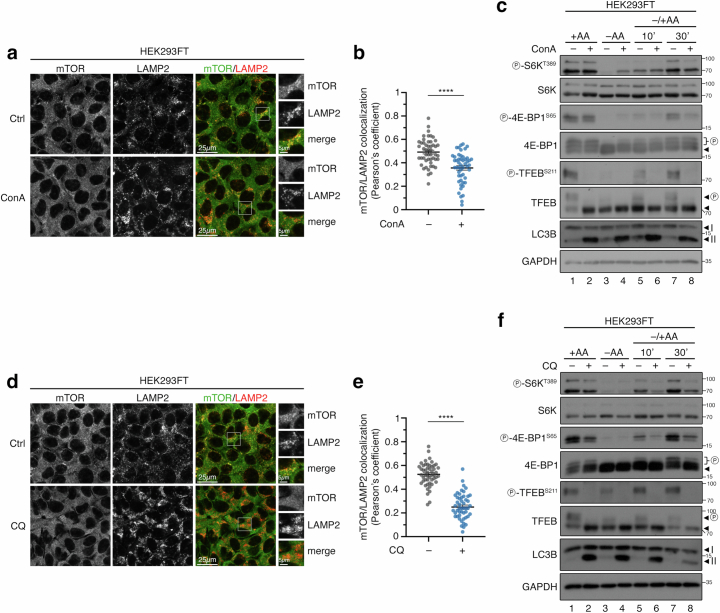
Blockage of lysosomal function using ConA or CQ disconnects mTORC1 localization on lysosomes from its activity towards cytoplasmic substrates. **(a-b)** Lysosomal accumulations of mTOR are lost in concanamycin A (ConA)-treated cells (100 nM, 6 h). Magnified insets shown to the right. Scale bars = 25 μm (for insets, 5 μm) (a). Quantification of mTOR/LAMP2 colocalization in (b). n = 50 individual cells from 5 independent fields per condition. **(c)** ConA treatment (100 nM) preferentially diminishes phosphorylation of the lysosomal substrate TFEB but not of the cytoplasmic substrates S6K and 4E-BP1 under basal culture conditions. ConA (or DMSO as control, Ctrl) was added directly in the media for 6 hours before lysis. For basal (+AA) conditions, culture media were replaced by +AA treatment media 90 min before lysis. For AA starvation (–AA), culture media were replaced by starvation media 1 h before lysis. For AA add-back samples (–/+AA), cells were first starved as described above and then starvation media were replaced by +AA treatment media for 10 or 30 min. ConA (or DMSO) was also included in the treatment media. The composition of all media is described in the Methods (see ‘Cell culture treatments’). **(d-f)** As in (a-c) but for treatments with chloroquine (CQ; 50 μM, 6 h). Arrowheads indicate bands corresponding to different protein forms, when multiple bands are present. P: phosphorylated form. For all panels, representative data from one out of three independent replicate experiments are shown. Data in graphs shown as mean ± SEM. **** p < 0.0001. Source numerical data and unprocessed blots are available in source data. Source data

**Extended Data Fig. 3:**
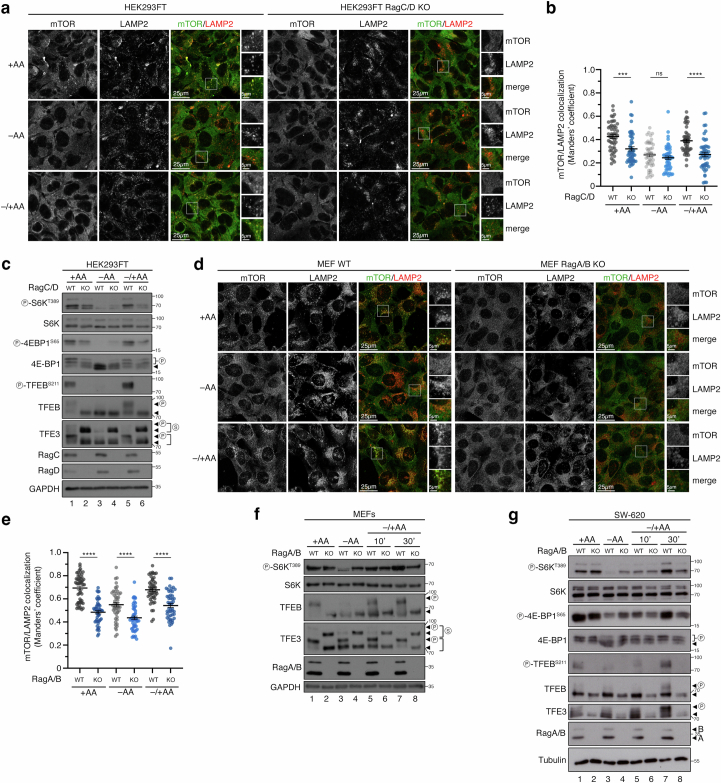
Rag loss-of-function disconnects mTORC1 localization and activity in different cell lines. **(a-b)** Colocalization analysis of mTOR with LAMP2 (lysosomal marker) in HEK293FT WT or RagC/D KO cells, treated as indicated in the figure, using confocal microscopy. For basal (+AA) conditions, culture media were replaced with +AA treatment media 90 min before fixation. For AA starvation (–AA), culture media were replaced by starvation media 1 h before fixation. For AA add-back samples (–/+AA), cells were first starved as described above and then starvation media were replaced by +AA treatment media for 30 min. The composition of all media is described in the Methods (see ‘Cell culture treatments’). Magnified insets shown to the right. Scale bars = 25 μm (for insets, 5 μm) (a). Quantification of colocalization in (b). n_WT(+AA)_ = 50, n_CDKO(+AA)_ = 50, n_WT(–AA)_ = 48, n_CDKO(–AA)_ = 49, n_WT(–/+AA)_ = 49, n_CDKO(–/+AA)_ = 50 individual cells from 5 independent fields per condition. Representative data from one out of three independent experiments are shown. **(c)** Immunoblots with lysates from HEK293FT WT and RagC/D KO cells, treated with media containing or lacking AAs, in basal (+AA), starvation (–AA) or add-back (–/+AA) conditions, probed with the indicated antibodies. Treatments were performed as in (a). n = 3 independent experiments. **(d-e)** Colocalization analysis of mTOR with LAMP2 (lysosomal marker) in WT or RagA/B KO MEF cells, treated as indicated in the figure, using confocal microscopy. Treatments were performed as in (a). Magnified insets shown to the right. Scale bars = 25 μm (for insets, 5 μm) (d). Quantification of colocalization in (e). n_WT(+AA)_ = 51, n_ABKO(+AA)_ = 50, n_WT(–AA)_ = 49, n_ABKO(–AA)_ = 50, n_WT(–/+AA)_ = 49, n_ABKO(–/+AA)_ = 49 individual cells from 3 independent fields per condition. Representative data from one out of two independent experiments are shown. **(f)** As in (c), but with WT and RagA/B KO MEFs. n = 3 independent experiments. **(g)** As in (c), but with WT and RagA/B KO SW-620 cells. n = 3 independent experiments. Arrowheads indicate bands corresponding to different protein forms, when multiple bands are present. P: phosphorylated form; S: SUMOylated form. Data in graphs shown as mean ± SEM. *** p < 0.001, **** p < 0.0001, ns: non-significant. Source numerical data and unprocessed blots are available in source data. Source data

mTORC1 localization and activity respond acutely and dynamically to changes in extracellular AA availability (Fig. 1d–g). Notably, although mTORC1 can be active and is found on lysosomes under either basal or AA resupplementation conditions (Fig. 1d–g), our previous work suggested that the mechanistic details of its activation (as indicated by the phosphorylation of its best-described substrate, S6K) may differ depending on the specific treatment strategy (fig. S1D in ref. ^23^). Most importantly, the involvement of virtually all components of the lysosomal AA-sensing machinery, including the Rags—as well as the upstream network that signals AA sufficiency via the Rags—has been studied using very consistently an ‘add-back’ treatment strategy, originally introduced by the Sabatini laboratory (50 min of AA starvation, followed by 10 min of AA re-addition). Apparently, this protocol only studies the acute re-activation of mTORC1, while overlooking the basal mTORC1 activation in unchallenged cells. Therefore, for subsequent experiments, we studied the regulation of mTORC1 under all aforementioned nutritional conditions (basal, starvation, and add-back of AAs) (Fig. 1d). In control HEK293FT cells, mTOR showed a mixed localization pattern with part of the signal colocalizing with LAMP2 (used as a lysosomal marker) and part being diffusely cytoplasmic. As opposed to control cells, mTOR no longer colocalized with lysosomes in cells treated with BafA1, regardless of the treatment regimen (Fig. 1e,f), thereby supporting our initial hypothesis about basal protein recycling and local AA production in lysosomes being the cause for the lysosomal recruitment of mTORC1. Next, we assessed the effect of BafA1 on the dynamics of mTORC1 activation by AAs. Unlike most previous studies that focused on specific substrates such as S6K to assay mTORC1 activity, we here tested multiple mTORC1 substrates. Interestingly, these experiments showed that phosphorylation of S6K and 4E-BP1, two canonical substrates of mTORC1, was largely unaffected by BafA1 under basal conditions (Fig. 1g). In contrast, the re-activation of mTORC1 upon AA re-addition was compromised in BafA1-treated cells (Fig. 1g), in line with a previous report using concanamycin A (ConA) to block lysosomal function and AA efflux from these organelles^27^. Strikingly, unlike for S6K and 4E-BP1, the phosphorylation of TFEB, a lysosomal non-canonical mTORC1 substrate, was abolished by BafA1 under all conditions tested (Fig. 1g), paralleling the loss of lysosomal mTORC1 accumulation (Fig. 1e,f). Of note, culturing cells in starvation media specifically lacking AAs readily caused the dephosphorylation of all mTORC1 substrates in both control and BafA1-treated cells (Fig. 1g). Similar results were obtained by (1) using ConA, an independent v-ATPase inhibitor (Extended Data Fig. 2a–c), (2) using chloroquine (CQ), a lysosomotropic weak base that alkalinizes the lysosomal lumen independently of v-ATPase inhibition (Extended Data Fig. 2d–f), (3) specifically inhibiting the activity of lysosomal proteases with a combination of pepstatin A (PepA) and E64 that does not affect lysosomal acidification (Fig. 2a–d) or (4) preventing the delivery of lysosomal enzymes to this compartment via transient knockdown or knockout (KO) of *N*-acetylglucosamine-1-phosphotransferase subunits alpha/beta (GNPTAB), a protein responsible for proper lysosomal enzyme sorting at the Golgi (Fig. 3a–h).

To test whether the persistent phosphorylation of S6K and 4E-BP1 in BafA1-treated cells is due to sustained activity of non-lysosomal mTORC1 or due to slower dephosphorylation kinetics, we performed BafA1 time-course experiments looking at both mTOR localization and activity, using S6K, 4E-BP1 and TFEB phosphorylation as read-outs. These experiments showed that mTOR lysosomal accumulations disappear already after 2 h of BafA1 treatment (Fig. 4a,b), closely correlating with the kinetics of TFEB dephosphorylation (Fig. 4c,d). In stark contrast, phosphorylation of S6K and 4E-BP1 stayed largely unaffected for the whole duration of the experiment (Fig. 4c,e). Dephosphorylation kinetics typically depend on the counteracting activities of a kinase and the respective phosphatase. Accordingly, by blocking mTORC1 kinase activity in control or RagA/B KO cells (in which mTOR is no longer lysosomal^18,22,23^, as in BafA1-treated cells; see also below) with rapamycin for different times, we looked at the rate of S6K dephosphorylation as a means to assess phosphatase activity. Notably, these experiments showed that phosphatase activity towards S6K is comparable between the two genotypes (Fig. 4f,g), further supporting that the persistent phosphorylation of cytoplasmic mTORC1 substrates in cells with non-lysosomal mTOR is not due to slower or impaired dephosphorylation (that is, due to compromised phosphatase activity).

In sum, using an orthogonal validation scheme, our data from five independent pharmacological or genetic perturbations that block basal lysosomal protein degradation (1) indicate the local AA production and release as the reason why a proportion of mTOR localizes to lysosomes, (2) reveal that non-lysosomal mTORC1 retains its activity towards its canonical substrates, but not towards its lysosomal substrates, under basal culture conditions and (3) show that the activity of this non-lysosomal mTORC1 pool is still regulated by exogenous AA availability. Therefore, we conclude that basal lysosomal proteolysis and local AA production are responsible for the lysosomal recruitment and regulation of mTORC1.

### The Rags are substrate- and location-specific mTORC1 regulators

**Fig. 5:**
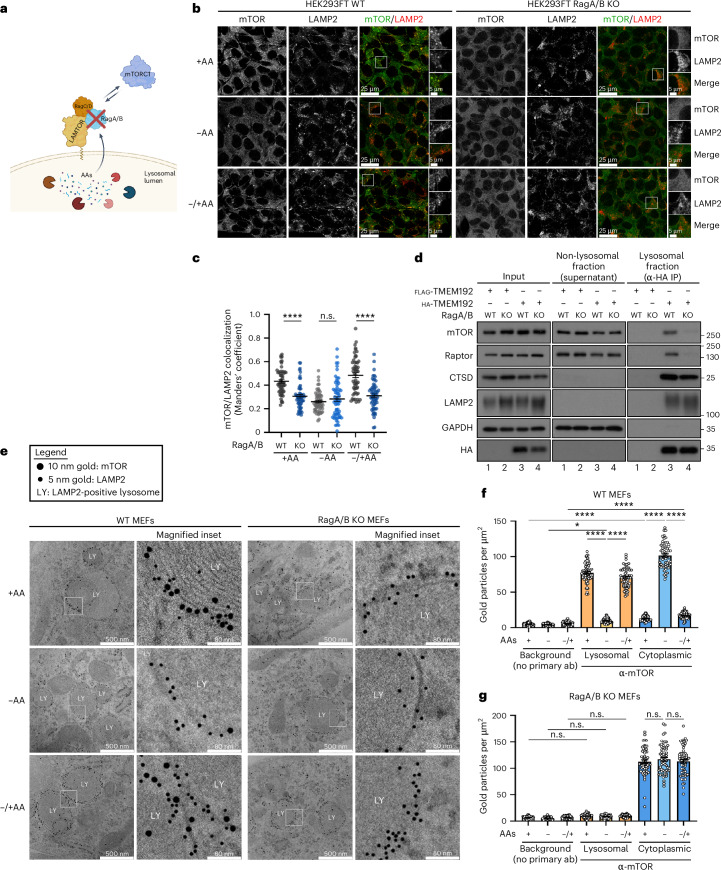
Diminished lysosomal localization of mTOR in Rag GTPase KO cells. **a**, A schematic model for the genetic removal of the Rag GTPases. **b**,**c**, Colocalization analysis of mTOR with LAMP2 (lysosomal marker) in HEK293FT WT and RagA/B KO cells (**b**) and its quantification (**c**), when treated as indicated, using confocal microscopy. For basal (+AA) conditions, culture media were replaced by +AA treatment media 90 min before fixation. For AA starvation (−AA), culture media were replaced by starvation media 1 h before fixation. For AA add-back samples (–/+AA), cells were first starved as described above and then starvation media were replaced by +AA treatment media for 30 min. The composition of all media is described in Methods. Magnified insets shown to the right in **b**. Scale bars, 25 μm and for insets, 5 μm. *n* = 55–60 individual cells from three or four independent fields per condition (see also Methods). **d**, Lyso-IP experiments with WT and RagA/B KO HEK293FT cells stably expressing HA-tagged TMEM192 (or FLAG-TMEM192 as negative control). Intact lysosomes were immunopurified by anti-HA IPs under native conditions, and the presence of the indicated proteins in lysosomal and non-lysosomal fractions as well as in whole-cell lysates was analysed by immunoblotting. **e**, Immuno-EM analysis of mTOR localization. Control (WT) or RagA/B KO MEFs, treated with media containing or lacking AAs, in basal (+AA), starvation (−AA) or add-back (–/+AA) conditions, treated as described in **b**, were stained with antibodies against endogenous mTOR (10 nm gold particles) and LAMP2 (5 nm gold particles) (**e**). Magnified insets shown on the right side; the area used for magnification is marked with a white square. Scale bars, 500 nm and for insets, 80 nm. LY, LAMP2-positive lysosomes. **f**,**g**, Quantification of mTOR localization at lysosomes or the cytoplasm in WT (**f**) or RagA/B KO MEFs (**g**), treated and analysed by immuno-EM as in **e**. Samples incubated with secondary antibodies only (no primary ab) were used as negative controls for background staining. Values represent number of gold particles per μm^2^. *n*_WT_ = 58–60 (**f**), *n*_KO_ = 60 (**g**) randomly selected areas (1 μm^2^ each) from three independent grids per condition. Data shown as mean ± s.e.m. *P < 0.05, ****P < 0.0001. n.s., non-significant. Source numerical data and unprocessed blots are available in Source data. See also Extended Data Fig. 3. Source data

As mTORC1 recruitment to lysosomes is mediated by the Rag GTPases, to genetically dissect the lysosomal from the non-lysosomal mTORC1 regulation and to study the functional consequences of lysosomal delocalization of mTORC1, we generated various Rag loss-of-function cell line models that lack RagA/B or RagC/D expression (Fig. 5a). As expected^18,22,23^, the lysosomal mTOR localization was diminished in RagA/B KO cells, under all nutritional conditions (Fig. 5b,c). To independently validate our microscopy data, we generated control or RagA/B KO cells stably expressing HA-tagged TMEM192 as a lysosomal anchor (or FLAG-tagged TMEM192 as a negative control), and developed a modified anti-HA lyso-immunoprecipitation (IP) protocol (original version described in ref. ^31^) that allowed us to isolate fractions enriched for intact lysosomes as well as non-lysosomal fractions. These experiments confirmed that a fraction of mTORC1 (shown by the presence of mTOR and Raptor) is specifically co-purified with lysosomes in a Rag-dependent manner, while both wild-type (WT) and RagA/B KO cells contain non-lysosomal mTORC1 complexes (Fig. 5d). Finally, we have expanded our mTOR localization analyses to quantitative immuno-electron microscopy (immuno-EM) experiments that provide superior subcellular resolution and allow the assignment of a given protein to a particular subcellular compartment with high confidence, especially when co-staining with specific organelle markers (for example, LAMP2 for lysosomes). Importantly, our immuno-EM data from WT or RagA/B KO MEFs independently validated the non-lysosomal localization of endogenous mTOR in RagA/B KO cells under all nutritional conditions, as well as in AA-starved WT cells (Fig. 5e–g).

**Fig. 6:**
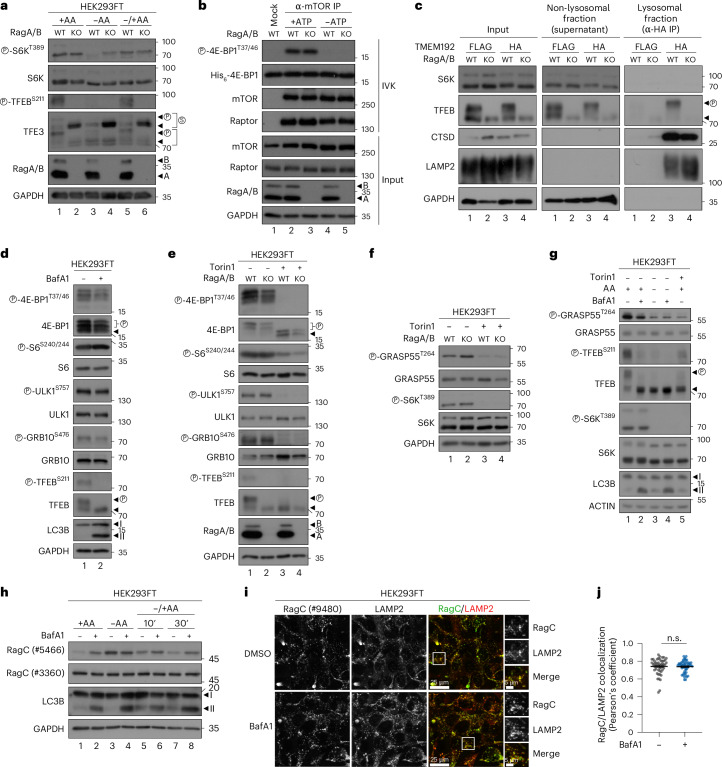
mTORC1 maintains activity towards its non-lysosomal substrates in cellular models of diminished lysosomal mTOR localization. **a**, Immunoblots with lysates from HEK293FT WT and RagA/B KO cells, treated with media containing or lacking AAs, in basal (+AA), starvation (−AA) or add-back (–/+AA) conditions, probed with the indicated antibodies. For basal (+AA) conditions, culture media were replaced by +AA treatment media 90 min before lysis. For AA starvation (−AA), culture media were replaced by starvation media 1 h before lysis. For AA add-back samples (–/+AA), cells were first starved as described above and then starvation media were replaced by +AA treatment media for 30 min. The composition of all media is described in Methods. **b**, In vitro kinase assays with mTORC1 immunopurified from WT or RagA/B KO HEK293FT cells and recombinant 4E-BP1 protein used as substrate, with 4E-BP1 phosphorylation detected by immunoblotting. No ATP samples (−ATP) used as negative controls. **c**, Lyso-IP experiments in WT and RagA/B KO HEK293FT cells stably expressing HA-tagged TMEM192 (or FLAG-TMEM192 as negative control). Intact lysosomes immunopurified by anti-HA IPs under native conditions, and the presence of the indicated proteins in the lysosomal and non-lysosomal fractions, as well as in whole-cell lysates, analysed by immunoblotting. Note the absence of S6K from lysosomal fractions and the presence of phospho-TFEB in the lysosomal fractions only of control cells. *n* = 2 independent experiments. **d**,**e**, Phosphorylation of multiple mTORC1 substrates is largely unaffected by BafA1 treatment (100 nM, 6 h) (**d**) or loss of Rag GTPases (**e**). In **e**, Torin1 (250 nM, 1 h) was used as a control for mTOR inhibition. **f**,**g**, GRASP55 phosphorylation by mTORC1 is retained in RagA/B KO (**f**) or BafA1-treated cells (100 nM, 6 h) (**g**), similarly to that of S6K. In **g**, starvation was performed as in **a**. Torin1 (250 nM, 1 h) was used as a control for mTOR inhibition. **h**, RagC is an additional lysosomal mTORC1 substrate that requires properly functioning lysosomes for its phosphorylation, similarly to TFEB/TFE3. AA starvation or blockage of lysosomal function with BafA1 (100 nM, 6 h) decrease RagC phosphorylation (shown as elevated RagC signal with #5466). Treatments performed as in **a**. **i**,**j**, Lysosomal localization of RagC is unaffected by BafA1 treatment (100 nM, 6 h) (**i**). Quantification of RagC/LAMP2 colocalization in (**j**). Scale bars, 25 μm and for insets, 5 μm. *n* = 50 individual cells from five independent fields per condition. Arrowheads indicate bands corresponding to different protein forms when multiple bands are present. P, phosphorylated form. Data in graphs shown as mean ± s.e.m. n.s., non-significant. Source numerical data and unprocessed blots are available in Source data. See also Extended Data Figs. 3–8. Source data

**Extended Data Fig. 4:**
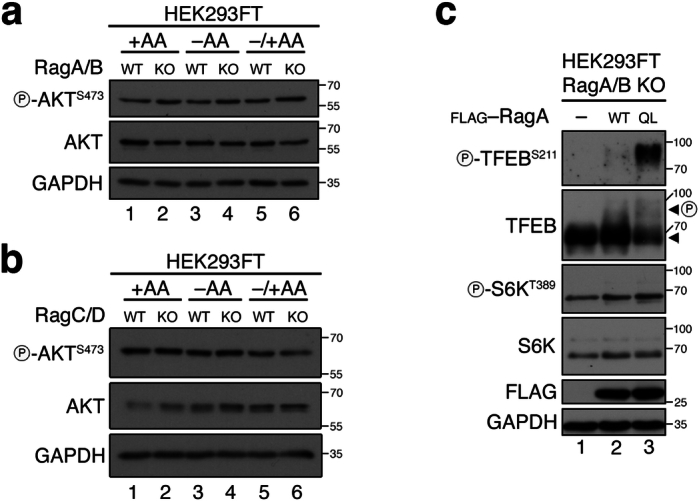
Rag loss-of-function does not affect mTORC2 activity, while its effect on mTORC1-TFEB is reversible by RagA re-expression. **(a)** Immunoblots with lysates from HEK293FT WT and RagA/B KO cells, treated with media containing or lacking AAs, in basal (+AA), starvation (–AA) or add-back (–/+AA) conditions, probed with the indicated antibodies. For basal (+AA) conditions, culture media were replaced with +AA treatment media 90 min before lysis. For AA starvation (–AA), culture media were replaced by starvation media 1 h before lysis. For AA add-back samples (–/+AA), cells were first starved as described above and then starvation media were replaced by +AA treatment media for 30 min. The composition of all media is described in the Methods (see ‘Cell culture treatments’). n = 2 independent experiments. **(b)** As in (a), but with WT and RagC/D KO HEK293FT cells. n = 3 independent experiments. (**c**) Re-expression of WT or active-locked RagA mutant (QL) in RagA/B KO cells rescues TFEB phosphorylation, without strongly affecting p-S6K levels. n = 2 independent experiments. Arrowheads indicate bands corresponding to different protein forms, when multiple bands are present. P: phosphorylated form. Unprocessed blots are available in source data. Source data

Interestingly, despite mTORC1 being non-lysosomal in RagA/B KO cells, phosphorylation of its canonical substrate S6K was largely unaffected in unchallenged cells grown under basal conditions (Fig. 6a), similar to our results using cells with perturbed lysosomal function (Figs. 1–4 and Extended Data Figs. 1 and 2). Confirming the well-established role of the Rags in the acute re-activation of mTORC1 towards S6K phosphorylation^18,22^, RagA/B KO cells showed blunted recovery of mTORC1 activity upon AA resupplementation, following AA depletion (Fig. 6a). Moreover, these cells demonstrated a partial resistance to AA starvation (Fig. 6a), which we and others have described previously^23,32^ and is due to loss of the Rag-mediated lysosomal recruitment of tuberous sclerosis complex (TSC; a negative regulator of mTORC1 activity) upon starvation^23^. In contrast to the behaviour of canonical substrate phosphorylation, that of the lysosomal TFEB/TFE3 substrates was completely abolished in RagA/B KO cells under all conditions of AA availability (Fig. 6a). Further strengthening the Rag-dependent separation of mTORC1 activities and showing that this is not a clonal artefact or a cell type-specific characteristic of the RagA/B KO HEK293FT cells, similar data were obtained using RagC/D KO HEK293FT cells (Extended Data Fig. 3a–c), RagA/B KO MEFs (Extended Data Fig. 3d–f) and RagA/B KO SW-620 colorectal cancer cells (Extended Data Fig. 3g). Moreover, the observed Rag- and AA-dependent effects were specific for mTORC1, as phosphorylation of AKT, a bona fide mTORC2 substrate was unaffected upon AA starvation or re-addition in control, RagA/B or RagC/D KO cells (Extended Data Fig. 4a,b). Finally, to assess the intrinsic activity of mTORC1, we immunopurified endogenous mTOR complexes from control or RagA/B KO cells and performed in vitro kinase (IVK) assays, using recombinant 4E-BP1 as a substrate. Strikingly, not only the phosphorylation of non-lysosomal mTORC1 substrates in cells, but also in vitro mTORC1 kinase activity was largely unaffected by Rag loss-of-function (Fig. 6b).

In support of our model that the Rags are involved primarily in the regulation of lysosomal mTORC1 signalling and the phosphorylation of its lysosomal substrates, exogenous expression of an active-locked RagA (Q66L) mutant in RagA/B KO cells caused a striking increase in TFEB phosphorylation, while it only marginally increased the phosphorylation of S6K. Re-expression of WT RagA also rescued TFEB phosphorylation, albeit to a much lesser extent (Extended Data Fig. 4c). These data are in line with a recent study from our group showing that stable reconstitution of a different Rag KO model with cancer-associated, activating RagC mutants (as a dimer with RagA) strongly increased TFEB/TFE3 phosphorylation without consistently affecting that of S6K (Fig. 5 in ref. ^21^). In sum, either loss or re-expression of the Rags only mildly influences mTORC1 activity towards S6K, while TFEB phosphorylation is fully dependent on the presence and the activation status of the Rag dimer.

In contrast to TFEB/TFE3, the canonical mTORC1 substrates such as S6K and 4E-BP1 were previously shown not to localize to lysosomes^27,33^ and are generally considered to be cytoplasmic mTORC1 substrates (with S6K partly also localizing in the nucleus^34^). Indeed, by lyso-IP experiments, we confirmed that S6K is found in the non-lysosomal fraction and does not co-purify with lysosomes of WT or RagA/B KO cells (Fig. 6c), in contrast to phosphorylated TFEB, which is found strongly enriched in the lysosomal fraction specifically of WT cells, but not Rag KOs. The absence of S6K localization on lysosomes is also supported by a previous proteomic study showing that the vast majority of p70-S6K1 interaction partners are cytosolic (or nuclear) proteins and not associated with lysosomes^35^. In sum, our data from multiple independent approaches suggest that mTOR, Raptor and S6K localize in the cytoplasm of Rag-deficient cells, where S6K phosphorylation likely takes place. This is consistent with the role of S6K in protein synthesis, a process that takes place primarily in the cytoplasm.

**Extended Data Fig. 5:**
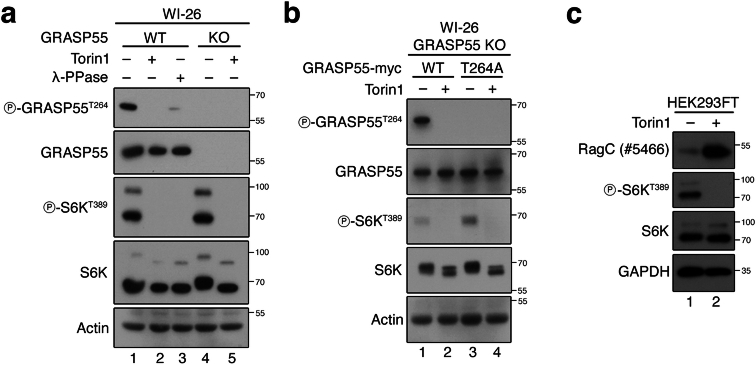
Characterization of reagents to study the phosphorylation of GRASP55 and RagC downstream of mTOR. **(a-b)** Characterization of the p-GRASP55 antibody. The signal from p-GRASP55 on T264 is diminished by Torin1 (250 nM, 2 h) or λ-phosphatase (λ-PPase) treatment, and is lost in WI-26 cells not expressing GRASP55 (a). GRASP55 KO WI-26 cells reconstituted with WT or T264A mutant GRASP55 (myc-tagged), and treated with Torin1, or DMSO as control. Note that p-GRASP55 signal is only detected in cells expressing WT GRASP55 with active mTORC1 (b). n = 2 independent experiments. **(c)** Downregulation of RagC phosphorylation by treatment with Torin1 (250 nM, 1 h), shown as an increase in RagC signal detected with the #5466 anti-RagC antibody from CST. n = 2 independent experiments. Unprocessed blots are available in source data. Source data

In line with our data on S6K and 4E-BP1, the phosphorylation of additional non-lysosomal mTORC1 substrates and downstream effectors such as ULK1, GRB10 and S6 was largely unaffected in BafA1-treated (Fig. 6d) or RagA/B KO cells (Fig. 6e), two conditions where mTOR is non-lysosomal and its lysosomal substrates such as TFEB are completely dephosphorylated. The Golgi re-assembly and stacking protein 55 (GRASP55) is a Golgi-residing protein that functions as a molecular switch for unconventional protein secretion (UPS), a cellular process activated upon starvation or stress. We have recently demonstrated that, when active, mTORC1 directly phosphorylates GRASP55 on Thr264 at the Golgi to inhibit UPS^36^. As with the other non-lysosomal substrates, the specific GRASP55 phosphorylation by mTORC1 was not affected by loss of the Rags, and only mildly affected by BafA1 treatment, as assayed with a custom-made phospho-specific antibody (Fig. 6f,g and Extended Data Fig. 5a,b). Finally, a recent study identified RagC as a direct mTORC1 substrate^37^. As its primary localization is on the lysosomal surface, we tested whether RagC phosphorylation requires lysosomal function or it behaves similarly to the cytoplasmic substrates. To achieve this, we made use of an anti-RagC antibody that is raised against the N-terminus of the protein and preferentially recognizes the non-phosphorylated RagC protein, therefore the signal in immunoblots anti-correlates with RagC phosphorylation. Accordingly, either Torin1 treatment (Extended Data Fig. 5c) or AA starvation (Fig. 6h, compare lanes 1 and 3) increased RagC signal. Notably, blocking lysosomal function and inhibiting lysosomal mTORC1 activity with BafA1 also led to a strong increase in RagC signal, indicating strongly decreased RagC phosphorylation (Fig. 6h), similarly to what we observed for another lysosomal mTORC1 substrate, TFEB. The dephosphorylation of RagC occurred despite the fact that its localization to lysosomes remained unaffected by BafA1 treatment (Fig. 6i,j), which indicates this is due to the delocalization of the kinase (that is, mTORC1) and not the substrate (that is, RagC) away from the lysosomal surface.

**Extended Data Fig. 6:**
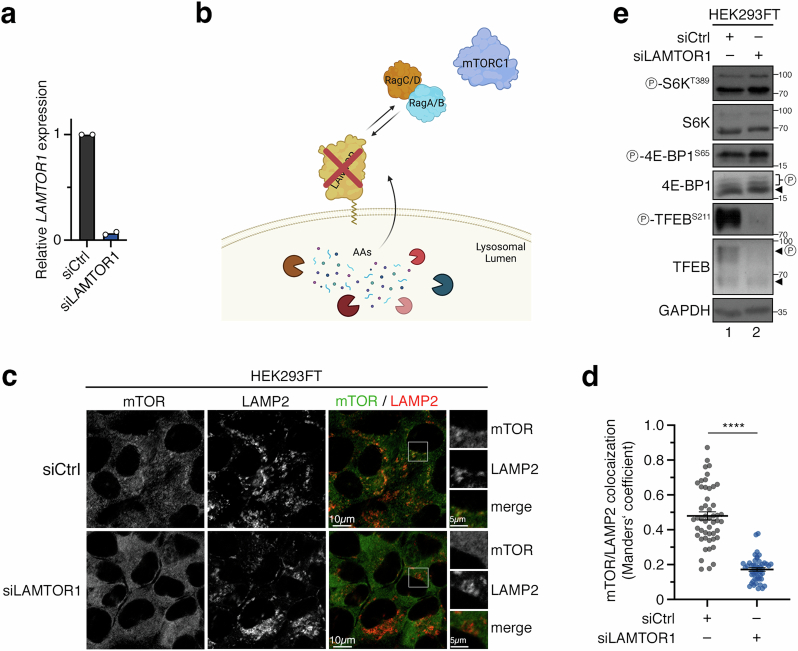
LAMTOR1/p18 knockdown recapitulates Rag loss-of-function, affecting primarily lysosomal mTORC1 signaling. **(a)** Expression analysis of *LAMTOR1* by qPCR confirms successful knockdown in HEK293FT cells. n = 2 independent experiments. **(b)** Schematic model of lysosomal tethering of the Rag dimer by the LAMTOR complex. **(c-d)** Colocalization analysis of mTOR with LAMP2 (lysosomal marker) in HEK293FT WT cells, using confocal microscopy. Cells were transiently transfected with siRNAs targeting *LAMTOR1* or a control RNAi duplex (siCtrl). Magnified insets shown to the right. Scale bars = 10 μm (for insets, 5 μm) (c). Quantification of colocalization in (d). n_siCtrl_ = 50, n_siLAMTOR1_ = 48 individual cells from 3 independent fields per condition. Representative data from one out of two independent experiments are shown as mean ± SEM. **** p < 0.001. **(e)** Immunoblots with lysates from HEK293FT WT cells, transiently transfected with siRNAs targeting *LAMTOR1* or a control RNAi duplex (siCtrl), cultured under basal conditions, and probed with the indicated antibodies. Arrowheads indicate bands corresponding to different protein forms, when multiple bands are present. P: phosphorylated form. n = 3 independent experiments. Source numerical data and unprocessed blots are available in source data. Source data

The Rag GTPase dimer is tethered to the lysosomal surface indirectly, via protein–protein interactions with the LAMTOR complex (also referred to as ‘Ragulator’)^22,38^. Therefore, we next transiently knocked down the LAMTOR1/p18 subunit of the LAMTOR complex (Extended Data Fig. 6a) as an additional means to dissociate mTORC1 from lysosomes, without perturbing the Rag dimer itself (Extended Data Fig. 6b). As expected, siLAMTOR1 cells showed diffuse cytoplasmic localization of mTOR with no lysosomal accumulations (Extended Data Fig. 6c,d). In agreement with our findings from Rag KO cells, *LAMTOR1* knockdown strongly diminished the phosphorylation of TFEB, without affecting S6K or 4E-BP1 phosphorylation under basal culture conditions (Extended Data Fig. 6e).

Overall, these data showed that, not only the Rags, but the complete lysosomal mTORC1 recruitment machinery is important for the regulation of the lysosomal mTORC1 substrates and that the spatial and functional separation of mTORC1 activities can be achieved, not only by perturbing the expression of the Rags, but also their anchoring to the lysosomal membrane. Moreover, we find that the Rags are largely dispensable for basal mTORC1 activity in unchallenged cells, while the re-activation of mTORC1 is Rag-dependent, as reported previously^18,23^. Finally, the Rag GTPases moonlight as location- and substrate-specific regulators of mTORC1.

### Cytoplasmic mTORC1 activation by exogenous AAs is Rag-network-independent

**Extended Data Fig. 7:**
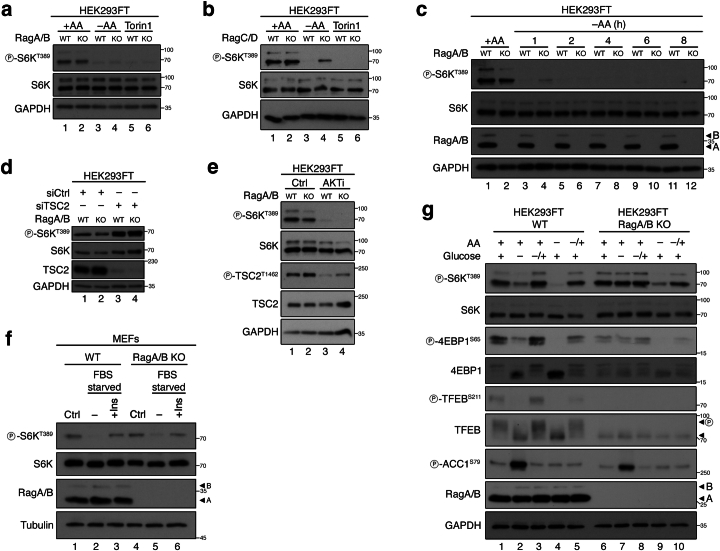
Phosphorylation of S6K by non-lysosomal mTORC1 responds to exogenous AAs and is regulated downstream of mTOR and growth factor signalling. **(a)** Immunoblots with lysates from WT and RagA/B KO HEK293FT cells, treated with media containing (+AA) or lacking AAs (–AA), or with Torin1 as shown, probed with the indicated antibodies. For basal (+AA) conditions, culture media were replaced by +AA treatment media 90 min before lysis. For AA starvation (–AA), culture media were replaced by starvation media 1 h before lysis. The composition of all media is described in the Methods (see ‘Cell culture treatments’). Torin1 (250 nM, 1 h) was used as a control for mTOR inhibition. n = 3 independent experiments. **(b)** As in (a), but for WT and RagC/D HEK293FT cells. n = 2 independent experiments. **(c)** AA starvation time-course (1-8 h) in WT and RagA/B KO HEK293FT cells shows that the residual S6K phosphorylation in the KOs at the early time points of starvation disappears at slightly later times. Starvation was performed as in (a). n = 3 independent experiments. **(d)** Immunoblots with lysates from WT and RagA/B KO HEK293FT cells, transiently transfected with siRNAs targeting *TSC2* or a control RNAi duplex (siCtrl), probed with the indicated antibodies. n = 2 independent experiments. **(e)** Immunoblots with lysates from WT and RagA/B KO HEK293FT cells, treated with Akt inhibitor (AKTi; 10 μM, 30 min) as shown, probed with the indicated antibodies. n = 3 independent experiments. **(f)** Growth factor removal and re-addition is sensed similarly in RagA/B KO and WT MEFs. Cells were starved for 1 h in media lacking FBS (–) and then re-stimulated with insulin (+Ins) for 30 min. n = 4 independent experiments. **(g)** Rag KO cells are deficient in sensing glucose depletion, in line with previous studies. WT and RagA/B KO HEKs were cultured under basal glucose- and AA-replete conditions, or starved with media lacking glucose or AAs for 1 h (–), or first starved and then re-stimulated (–/+) with glucose or AAs for 30 min. n = 3 independent experiments. Arrowheads indicate bands corresponding to different protein forms, when multiple bands are present. P: phosphorylated form. Unprocessed blots are available in source data. Source data

Using multiple genetic and pharmacological ways to target the lysosomal AA-sensing machinery, we dissociated the lysosomal localization of mTORC1 from its activity, revealing that non-lysosomal mTORC1 remains active towards its non-lysosomal substrates, including S6K and 4E-BP1. Indeed, by treating RagA/B or RagC/D KO cells with Torin1, a catalytic mTOR inhibitor, we confirmed that the S6K phosphorylation in these models is indeed mTOR kinase activity-dependent (Extended Data Fig. 7a,b). As mentioned above, albeit partially resistant at the early timepoints of AA starvation^23^, these cells do respond to AA starvation, with complete mTORC1 inactivation achieved at slightly later timepoints, thus showing that non-lysosomal mTORC1 can be regulated by exogenous AA availability (Extended Data Fig. 7a–c).

The TSC/Rheb signalling hub lies directly upstream of mTORC1, integrating information from most stimuli that regulate mTORC1 activity, including AAs and growth factors^4,23,39–41^. Transient downregulation of TSC2 in control or RagA/B KO cells—that leads to hyperactivation of Rheb—robustly elevated mTORC1 activity in both genotypes (Extended Data Fig. 7d), showing that these upstream regulators are relevant also for the activity of non-lysosomal mTORC1 towards S6K. Consistent with this, blocking growth factor signalling by using a pharmacological Akt inhibitor decreased S6K phosphorylation in RagA/B KO and control cells to a similar extent (Extended Data Fig. 7e). Accordingly, serum starvation and insulin stimulation experiments in Rag KO MEFs confirmed that mTORC1 activity properly responds to growth factors in Rag-deficient cells, as in the respective Rag-proficient cells (Extended Data Fig. 7f). However, in line with previous work on the role of the Rags in glucose signalling to mTORC1 on lysosomes (for example, fig. 2c in ref. ^32^), its activity (assayed by the phosphorylation of S6K and 4E-BP1) did not respond to glucose starvation (Extended Data Fig. 7g, compare lanes 6 and 7), while it was readily downregulated by AA starvation (Extended Data Fig. 7g, compare lanes 6 and 9). Importantly, this demonstrates that non-lysosomal mTORC1 specifically senses the absence of exogenous AAs, but not of glucose.

**Fig. 7:**
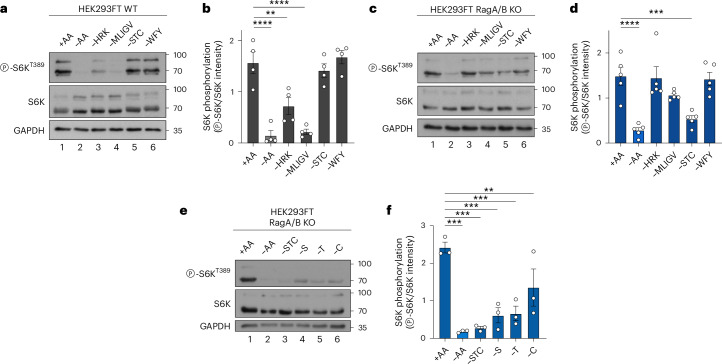
Non-lysosomal mTORC1 is regulated by distinct exogenous AAs. **a**,**b**, Immunoblots with lysates from HEK293FT WT cells treated for 1 h with media containing or lacking the AA groups as shown and probed with the indicated antibodies (**a**) and quantification of mTORC1 activity (p-S6K^T389^/S6K) (**b**). *n* = 4 independent experiments. **c**,**d**, As in **a** and **b** but for RagA/B KO HEK293FT cells (**c**) and quantification of mTORC1 activity (p-S6K^T389^/S6K) (**d**). *n* = 5 independent experiments. **e**,**f**, As in **c** and **d** but for RagA/B KO HEK293FTs treated with media lacking the indicated groups or individual AAs for 1 h (**e**) and quantification of mTORC1 activity (p-S6K^T389^/S6K) (**f**). Data in graphs shown as mean ± s.e.m. **P < 0.01, ***P < 0.001, ****P < 0.0001. Source numerical data and unprocessed blots are available in Source data. Source data

**Extended Data Fig. 8:**
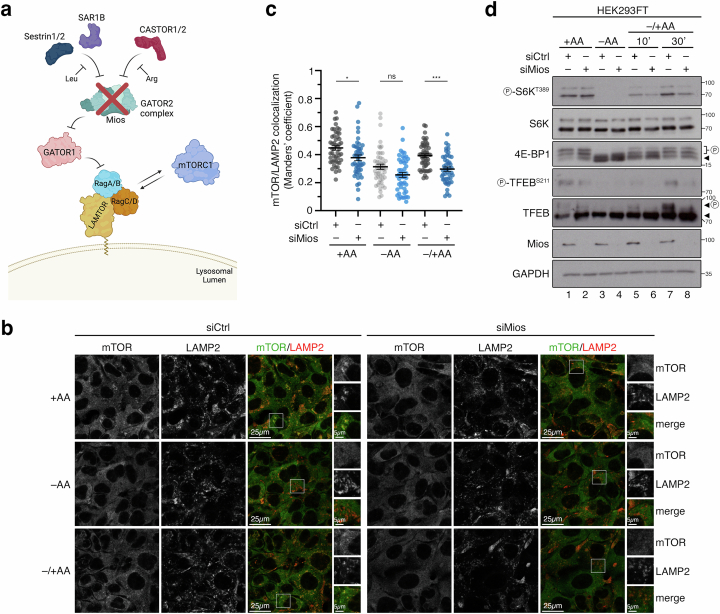
Non-lysosomal mTORC1 is regulated independently of the GATOR2 complex. **(a)** Schematic model of cytoplasmic AA sensing and signaling upstream of the Rags. See text for details. **(b-c)** Colocalization analysis of mTOR with LAMP2 (lysosomal marker) in HEK293FT WT cells, using confocal microscopy. Cells were transiently transfected with siRNAs targeting *Mios* or a control RNAi duplex (siCtrl) and treated as indicated. For basal (+AA) conditions, culture media were replaced with +AA treatment media 90 min before fixation. For AA starvation (–AA), culture media were replaced by starvation media 1 h before fixation. For AA add-back samples (–/+AA), cells were first starved as described above and then starvation media were replaced by +AA treatment media for 30 min. The composition of all media is described in the Methods (see ‘Cell culture treatments’). Magnified insets shown to the right. Scale bars = 25 μm (for insets, 5 μm) (b). Quantification of colocalization in (c). n_siCtrl(+AA)_ = 49, n_siMios(+AA)_ = 46, n_siCtrl(–AA)_ = 49, n_siMios(–AA)_ = 47, n_siCtrl(–/+AA)_ = 50, n_siMios(–/+AA)_ = 46 individual cells from 5 independent fields per condition. Representative data from one out of two independent experiments are shown. **(d)** Immunoblots with lysates from HEK293FT WT cells, transiently transfected with siRNAs targeting *Mios* or a control RNAi duplex (siCtrl), and treated with media containing or lacking AAs, in basal (+AA), starvation (–AA) or add-back (–/+AA; 10 or 30 min) conditions, probed with the indicated antibodies. Treatments were performed as in (b). n = 3 independent experiments. Arrowheads indicate bands corresponding to different protein forms, when multiple bands are present. P: phosphorylated form. Data in (c) shown as mean ± SEM. * p < 0.05, *** p < 0.001, ns: non-significant. Source numerical data and unprocessed blots are available in source data. Source data

The fact that the non-lysosomal activity of mTORC1 is under the control of exogenous AA sources hints at the existence of Rag- and lysosome-independent regulatory mechanisms via which different AA pools signal to mTORC1. The Sestrin1/2 and SAR1B proteins are cytoplasmic sensors for leucine, while the CASTOR1/2 proteins sense arginine levels, with all of them signalling AA availability to the Rag GTPases via the GATOR2 complex^42–48^ (Extended Data Fig. 8a). Therefore, we performed transient knockdown experiments targeting Mios, a key component of the GATOR2 protein complex, as a tool to study the possible involvement of the cytoplasmic AA sensors in the regulation of non-lysosomal mTORC1. Although downregulation of Mios strongly decreased lysosomal mTOR localization (Extended Data Fig. 8b,c), consistent with its previously described role in AA sensing upstream of the Rags^49,50^, and suppressed TFEB phosphorylation under all nutritional conditions, the phosphorylation of S6K and 4E-BP1 was largely unaffected in cells grown under basal conditions (Extended Data Fig. 8d). As observed also for cells with Rag loss-of-function, Mios knockdown potently blunted the re-activation of mTORC1 upon AA re-addition (Extended Data Fig. 8d). Therefore, although this complex was described to integrate information from proteins that sense AA sufficiency in the cytoplasm, it still signals through the lysosomal Rag-related machinery to regulate mTORC1. These data suggest that extracellular AAs signal to non-lysosomal mTORC1 via mechanisms that have not been resolved yet. Consistent with this hypothesis, we found that RagA/B KO cells respond to different AA groups, compared to their WT counterparts. In particular, while mTORC1 activity in Rag-proficient control cells is sensitive to depletion of hydrophobic (methionine, leucine, isoleucine, glycine, valine; ‘–MLIGV’) or positively charged (histidine, arginine, lysine; ‘–HRK’) AAs from the culture media (Fig. 7a,b), RagA/B KO cells do not respond significantly to removal of these AA groups (Fig. 7c,d). Interestingly, treatment with starvation media specifically lacking serine, threonine and cysteine (‘–STC’) downregulated mTORC1 activity in RagA/B KO, but not in control cells (Fig. 7a–d). In addition, each of the AAs within the ‘–STC’ mix was necessary for mTORC1 to stay active in Rag KO cells, as treating cells with media lacking serine, threonine or—to a lesser extent—cysteine, singly, downregulated mTORC1 comparably to the combined removal of S+T+C (Fig. 7e,f). Because Rag-deficient cells (in which mTORC1 is non-lysosomal) demonstrate differential sensitivity of mTORC1 activity to distinct AA subgroups, compared with Rag-proficient cells that show considerable lysosomal mTORC1 localization, it is intriguing to predict that different sensing mechanisms may exist to regulate mTORC1 at the two subcellular locations. Importantly, whereas the lysosomal Rag-related AA-sensing machinery has been described extensively, the proteins and pathways that signal the availability of specific exogenous AAs to non-lysosomal mTORC1 (directly, by AAs binding to cytoplasmic sensors, or indirectly, by sensing the levels of downstream metabolites) are completely unknown. Future endeavours will be necessary to put together a larger part of this AA-sensing network.

**Extended Data Fig. 9:**
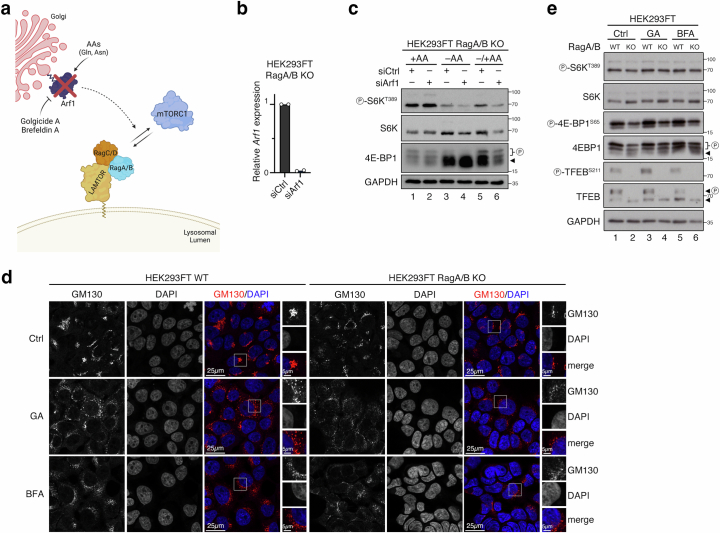
Non-lysosomal mTORC1 activity is independent of Arf1 and the Golgi apparatus. **(a)** Schematic model of Golgi-localized Arf1 regulating mTORC1 localization and activity in response to re-addition of specific AAs. Golgicide A (GA) or Brefeldin A (BFA) block Arf1 via targeting the ArfGEF GBF1. **(b)** Expression analysis of *Arf1* by qPCR confirms successful knockdown in RagA/B KO HEK293FT cells. n = 2 independent experiments. **(c)** Immunoblots with lysates from RagA/B KO HEK293FT cells, transiently transfected with siRNAs targeting *Arf1* or a control RNAi duplex (siCtrl), and treated with media containing or lacking AAs, in basal (+AA), starvation (–AA) or add-back (–/+AA) conditions, probed with the indicated antibodies. For basal (+AA) conditions, culture media were replaced with +AA treatment media 90 min before lysis. For AA starvation (–AA), culture media were replaced by starvation media 1 h before lysis. For AA add-back samples (–/+AA), cells were first starved as described above and then starvation media were replaced by +AA treatment media for 30 min. The composition of all media is described in the Methods (see ‘Cell culture treatments’). n = 3 independent experiments. **(d)** Golgi morphology using GM130 (Golgi marker) in HEK293FT WT and RagA/B KO cells, using confocal microscopy. Cells treated with Golgicide A (GA; 10 μM, 1 h) or Brefeldin A (BFA; 10 μg/ml, 1 h) as indicated. Magnified insets shown to the right. Scale bars = 25 μm (for insets, 5 μm). n = 3 independent experiments. **(e)** Immunoblots with lysates from WT and RagA/B KO HEK293FT cells, treated with GA or BFA as shown in the panel, probed with the indicated antibodies. n = 3 independent experiments. Arrowheads indicate bands corresponding to different protein forms, when multiple bands are present. P: phosphorylated form. Source numerical data and unprocessed blots are available in source data. Source data

Glutamine or asparagine resupplementation was recently reported to re-activate mTORC1 (towards its canonical substrates) via the Golgi-localized Arf1 GTPase and independently of the Rags^51,52^ (Extended Data Fig. 9a). Therefore, we next tested whether Arf1 is involved in the regulation of non-lysosomal mTORC1, by performing transient knockdown experiments in RagA/B KO cells (Extended Data Fig. 9b). Although Arf1 was indeed important for full re-activation of mTORC1 upon AA add-back, it did not influence its activity under basal conditions (Extended Data Fig. 9c). Similarly, treatment with golgicide A (GA) or brefeldin A (BFA), two drugs that target the ArfGEF GBF1 (Extended Data Fig. 9a), blocked Arf1 activation, as shown by the robust structural and morphological changes of the Golgi apparatus (Extended Data Fig. 9d). These treatments did not alter mTORC1 activity in either RagA/B KO or control cells, towards any of its substrates (Extended Data Fig. 9e). Therefore, like the Rags that are dispensable for basal mTORC1 activity, Arf1 is seemingly also involved only in the re-activation of mTORC1 upon resupplementation with specific AAs, for example, glutamine. Similar results were obtained previously in yeast cells, in which glutamine can regulate TORC1 in the absence of the yeast Rag homologues^53^. Taken together, these data suggest that non-lysosomal mTORC1 activation by specific exogenous AAs occurs independently of known Rag- and lysosome-related signalling network components.

### Spatially distinct mTORC1 complexes control different cellular processes

**Fig. 8:**
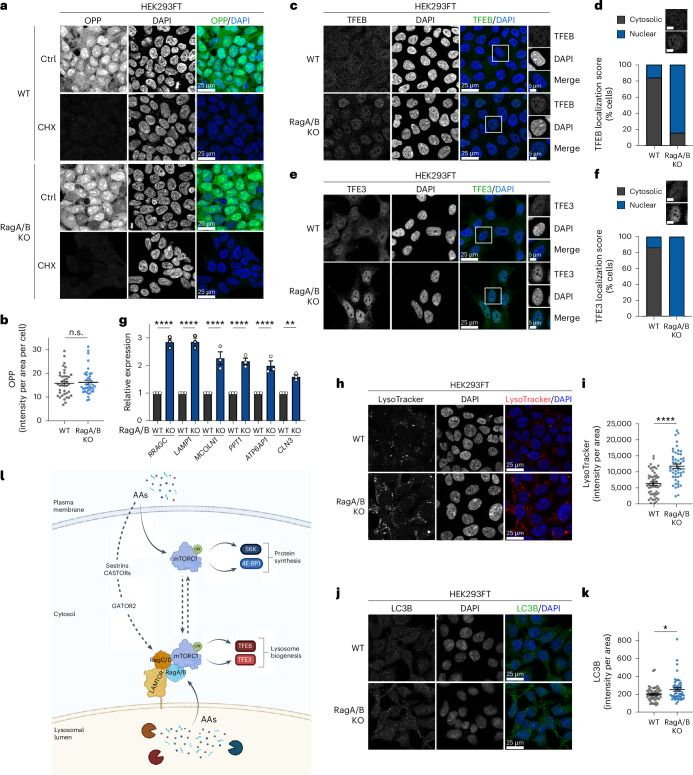
Functional separation of Rag-dependent and Rag-independent mTORC1 activities. **a**,**b**, De novo protein synthesis (OPP incorporation) assay using WT and RagA/B KO (ABKO) HEK293FT cells (**a**) and quantification of OPP signal (**b**). Nuclei stained with DAPI. Cycloheximide (CHX) inhibitor used as negative control. Scale bars, 25 μm (**a)**. *n* = 40 individual cells from five independent fields per condition. **c**,**d**, TFEB localization analysis in WT and RagA/B KO HEK293FT cells using confocal microscopy (**c**), and scoring of TFEB localization (**d**). Nuclei stained with DAPI. Magnified insets shown to the right in **c**. Scale bars, 25 μm and for insets, 5 μm. Individual cells were scored for nuclear or cytoplasmic TFEB localization as indicated in the example images. *n*_WT_ = 65 cells, *n*_ABKO_ = 102 cells. **e**,**f**, As in **c** and **d** but for TFE3 localization (**e**) with scoring in (**f**). *n* = 52 cells. **g**, Expression analysis of TFEB/TFE3 target genes in HEK293FT WT and RagA/B KO cells. **h**,**i**, LysoTracker staining in HEK293FT WT and RagA/B KO cells (**h**) and quantification of LysoTracker signal (**i**). Nuclei stained with DAPI. Scale bars, 25 μm (**h**). *n*_WT_ = 50, *n*_ABKO_ = 49 individual cells from five independent fields per condition. Representative data from one out of two independent experiments are shown. **j**,**k**, LC3B staining in HEK293FT WT and RagA/B KO cells (**j**) and quantification of LC3B signal (**k**). Nuclei stained with DAPI. Scale bars, 25 μm (**j**). *n*_WT_ = 50, *n*_ABKO_ = 48 individual cells from five independent fields per condition. **l**, Working model for the functional and spatial separation of mTORC1 activities in cells. See main text for details. Data in **b**, **g**, **i** and **k** shown as mean ± s.e.m. *P < 0.05, **P < 0.01, ****P < 0.0001. Source numerical data are available in Source data. See also Extended Data Fig. 9. Source data

**Extended Data Fig. 10:**
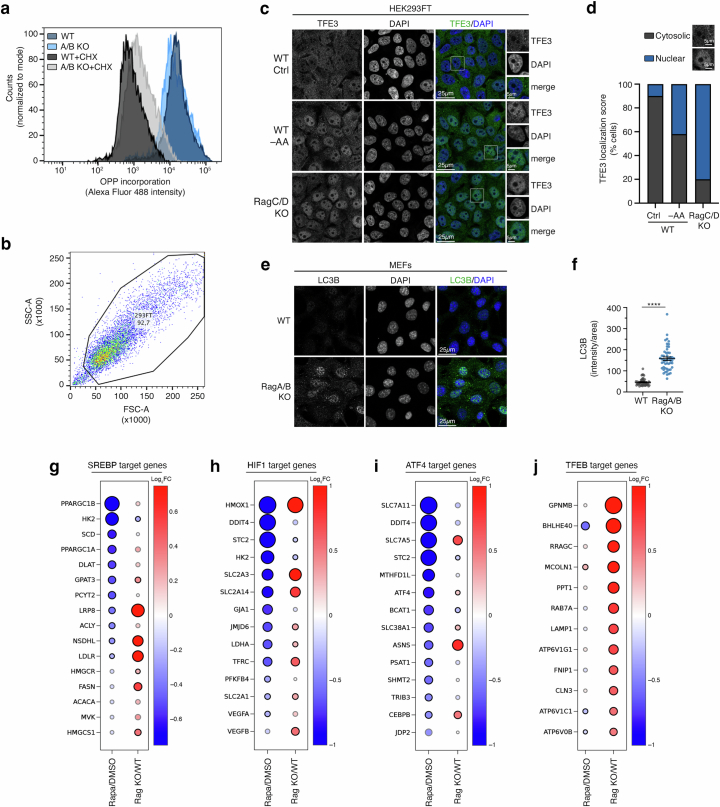
Functional separation of Rag-dependent and -independent mTORC1 activities. **(a)** *De novo* protein synthesis (OPP incorporation) assay with WT and RagA/B KO HEK293FT cells, analysed by flow cytometry. Cycloheximide (CHX) used as control to block protein synthesis. Cell counts: n_WT_ = 9306, n_ABKO_ = 9317, n_WT+CHX_ = 9572, n_ABKO+CHX_ = 8869. Representative data from one out of three independent experiments are shown. **(b)** Gating strategy for the flow-cytometry-based OPP incorporation assay in (a). **(c-d)** TFE3 localization analysis in WT and RagC/D KO HEK293FT cells, using confocal microscopy. AA starvation (–AA, 1 h) was used as control. Nuclei stained with DAPI. Magnified insets shown to the right. Scale bars = 25 μm (for insets, 5 μm) (c). Scoring of TFE3 localization in (d). Individual cells were scored for nuclear or cytoplasmic TFEB localization as indicated in the example images. n_WT(Ctrl)_ = 81 cells, n_WT(–AA)_ = 92 cells, n_ABKO_ = 103 cells. Representative data from one out of three independent experiments are shown. **(e-f)** LC3B staining in WT and RagA/B KO MEFs. Nuclei stained with DAPI. Scale bars = 25 μm (e). Quantification of LC3B signal in (f). n = 50 individual cells from 5 independent fields per condition. Representative data from one out of two independent experiments are shown. **(g)** Gene expression analyses from RNA-seq experiments comparing rapamycin- to control-treated HEK293FT cells (Rapa/DMSO) or RagA/B KO to WT HEK293FT cells (Rag KO/WT) indicate SREBP activity is not consistently affected by loss of the Rags. Dot plot showing the changes in the expression of selected SREBP target genes in each of the two datasets. For each dot, colour and size indicate log_2_-transformed fold change (Log_2_FC), and outline colour indicates significance (black: adj. p-value < 0.05; grey: adj. p-value ≥ 0.05). **(h)** As in (g), but for selected HIF1 target genes. **(i)** As in (g), but for selected ATF4 target genes. **(j)** As in (g), but for selected TFEB target genes. Note the specific upregulation of lysosome-related genes in the RagA/B KO vs. WT comparison, whereas no such changes are observed in the respective Rapamycin vs. DMSO comparison. Data in (f) shown as mean ± SEM. **** p < 0.0001. Source numerical data are available in source data. Source data

We describe here the spatial separation of mTORC1 activities towards different substrates and downstream effectors. Therefore, we next sought to investigate what is the physiological function of these two distinct mTORC1 entities in cells. As mTORC1 is known to regulate protein synthesis via the phosphorylation of S6K and 4E-BP1, we first assessed de novo protein synthesis by using a modified puromycin incorporation assay (*O*-propargyl-puromycin (OPP) assay), comparing RagA/B KO with control cells. Consistent with S6K and 4E-BP1 phosphorylation being largely unaffected in *Rag*-null cells grown in AA-replete media, de novo protein synthesis was independent of Rag presence (Fig. 8a,b and Extended Data Fig. 10a,b). Therefore, mTORC1 regulates protein synthesis independently of the Rag GTPases and the lysosomal AA-sensing machinery.

Unlike for S6K and 4E-BP1, the mTORC1-dependent phosphorylation of the TFEB and TFE3 transcription factors (TFs) is abolished in Rag-deficient cells. Accordingly, dephosphorylation of TFEB and TFE3 in RagA/B KO cells was accompanied by increased nuclear localization of these TFs (Fig. 8c–f). Similar data were obtained for TFE3 localization in RagC/D KO cells (Extended Data Fig. 10c,d). In the nucleus, TFEB/TFE3 upregulated the expression of target genes that are related to lysosome and autophagosome biogenesis (Fig. 8g), in agreement with previous studies^54–57^. Indeed, RagA/B KO HEK293FT cells showed an overall increase in lysosome abundance (assessed by LysoTracker staining) (Fig. 8h,i) as well as increased autophagosome content (shown by LC3 immunofluorescence) (Fig. 8j,k). Similar results were obtained in MEFs, with RagA/B KO cells demonstrating elevated LC3 levels (Extended Data Fig. 10e,f).

A vast gene expression programme is regulated downstream of mTORC1, directly or indirectly, via the activity of TFs such as SREBP, HIF1 (ref. ^58^) and ATF4 (ref. ^59^). To investigate whether this mTORC1-dependent gene expression programme depends on the presence of the Rags, we performed RNA-sequencing (RNA-seq) experiments and tested the expression of selected target genes for each of these TFs, comparing WT and RagA/B KO HEK293FT cells. As a control, we re-analysed our recently published RNA-seq dataset from rapamycin- versus dimethylsulfoxide (DMSO)-treated HEK293FT cells, in which mTORC1 is inhibited and S6K is dephosphorylated^60^. These analyses showed that selected SREBP, HIF1 and ATF4 target genes were consistently downregulated upon mTORC1 inhibition with rapamycin, confirming that the activity of these TFs is regulated downstream of mTORC1 (Extended Data Fig. 10g–i). In contrast, the expression of these target genes was not consistently affected in Rag KO cells (Extended Data Fig. 10g–i), indicating that these TFs do not require the activity of lysosomal mTORC1, and thus are probably regulated by non-lysosomal mTORC1 entities. As a control, we expanded our quantitative PCR (qPCR) analyses described above (Fig. 8g) to check the expression of additional TFEB targets in the two RNA-seq datasets. As expected from the loss of TFEB phosphorylation and its nuclear translocation in Rag KO cells, also the expression of its target genes was strongly upregulated (Extended Data Fig. 10j). Moreover, consistent with TFEB being a rapamycin-resistant substrate of mTORC1 (ref. ^61^), expression of these TFEB targets was largely unaffected in rapamycin-treated cells (Extended Data Fig. 10j). These data highlight the mTORC1-dependent transcriptional programme as an additional physiological cellular process that is specifically controlled by non-lysosomal (for example, for SREBP, HIF1 and ATF4 targets) or lysosomal mTORC1 (for example, for TFEB targets). Taken together, we report here that the lysosomal and non-lysosomal mTORC1 entities regulate distinct cellular functions via the differential phosphorylation of distinct mTORC1 substrates at these subcellular locations (Fig. 8l).

## Discussion

The reason for a lysosome-centred regulation of mTORC1 has been a major field of discussion since its introduction in 2008 (discussed in ref. ^26^). mTORC1 re-activation ‘makes sense’ to happen at lysosomes, where bulk macro-autophagy takes place upon AA starvation, degrading proteins to supply cells with fresh AAs, which in turn re-activate mTORC1. Therefore, mTORC1 is meaningfully localized in the vicinity of AA resupplementation—following nutrient starvation—and posed for re-activation. However, this model does not sufficiently explain why mTORC1 should be activated on lysosomes in non-starved, unchallenged cells. Indeed, although the Rag GTPases localize primarily on lysosomes and regulate mTORC1 by recruiting it there^22^, recent studies by us and others provide evidence that the lysosomal localization of mTORC1 and its activity (assayed by phosphorylation of S6K in most cases) are not always interrelated^21,23,27,51,62–65^. Consistent with this, its direct regulators (for example, Rheb, Rags and Arf1)^27,28,51,66–71^, many of its substrates^27,72–74^ and mTOR itself^27,28,36,68,70,73,75–79^ were also described to localize at multiple subcellular locations, besides lysosomes^26^. Furthermore, the relocalization of mTORC1 to lysosomes in response to AA resupplementation is seemingly transient, dynamic and involves only a fraction of the total cellular mTOR pool^27,28^. Therefore, our study supports a model whereby the regulation of mTORC1 signalling by AAs also involves non-lysosomal locations and thus Rag-independent mechanisms that function in parallel to the lysosome-based, Rag-related AA-sensing network (Fig. 8l). On the basis of these previous data, we hypothesize that these mTORC1 entities are not strictly separated from each other in WT cells, but instead the complexes dynamically relocalize between the lysosomal surface, the cytoplasm and presumably other organelles too (for example, the Golgi), in response to local AA stimuli. Such a spatial separation of mTORC1 regulation and activities, with mTORC1 phosphorylating different substrates at distinct, functionally relevant subcellular locations, would intuitively confer specificity and compartmentalization of its function. Simultaneously, the fact that exogenous AAs also signal to activate lysosomal mTORC1 signalling towards TFEB/TFE3 (for example, via binding to the respective cytoplasmic AA sensors)^42–47^, and that lysosomal recruitment and re-activation of mTORC1 (for example, upon AA resupplementation) are required also for the robust rephosphorylation of its cytoplasmic substrates, such as S6K^18,19,22,23^, suggest the existence of an intricate interplay between the cytoplasmic and the lysosomal regulation of mTORC1 in WT cells that remains to be elucidated in the future.

Previous attempts to identify novel regulators of dTOR (*Drosophila* TOR) activity in a genome-wide manner in untreated *Drosophila* cells, using the phosphorylation of S6 (a direct S6K substrate) as a read-out, failed to identify any Rag GTPase or LAMTOR orthologues among the hits^80^. Furthermore, animal models lacking Rag activity also support the notion that additional, Rag-independent mechanisms play an important role in regulating mTORC1. According to the previously described mouse models, RagA-null animals die around embryonic day (E)10.5 (ref. ^32^). Moreover, LAMTOR2-null mouse embryos, which lose proper Rag localization and activity, also die shortly before E10.5 (ref. ^81^). In contrast, mTOR and Raptor KOs die much earlier in embryogenesis (ca. E3.5 (refs. ^82,83^) and E6.5 (refs. ^84^), respectively), indicating that Rag loss-of-function does not phenocopy mTORC1 inactivation. In line with this, MEFs extracted from RagA-null mice grow similarly to their WT counterparts and exhibit persistent mTORC1 activity (assayed by phosphorylation of its canonical substrates), despite having blunted mTORC1 lysosomal localization^32^. In another mouse model, deletion of both RagA and RagB slightly reduced (approximately 30%) but did not abolish mTORC1 activity in MEFs^51^ or in cardiomyocytes^85^. Genetic studies in zebrafish also showed that TORC1 signalling is apparently normal in RagA-mutant larvae^86^. Hence, mTORC1 is active also in the absence of RagA/B, and therefore the Rags seem largely dispensable for physiological mTORC1 activation towards its canonical substrates.

Some of these previous studies interpreted the persistent phosphorylation of S6K in Rag-deficient cells or animals as the result of compensatory activation of other signalling pathways that lead to mTORC1 activation. Our findings, however, show that this is not the case, for several reasons: (1) the sustained S6K phosphorylation is indeed dependent on mTOR activity (Extended Data Fig. 7a,b), (2) transient LAMTOR knockdown mimics the effects of chronic Rag depletion (Extended Data Fig. 6), (3) short-term pharmacological interventions that perturb lysosomal function and local AA production and efflux are also sufficient to recapitulate the Rag KO phenotype (Figs. 1, 2 and 4 and Extended Data Fig. 2), (4) the phosphorylation of the lysosomal mTORC1 substrates (that is, TFEB, TFE3 and RagC) is actually diminished in all pharmacological or genetic models that we use here, therefore mTORC1 is selectively active towards its non-lysosomal substrates, (5) transient re-expression of active RagA is sufficient to rescue lysosomal activity of mTORC1 in RagA/B KO cells (Extended Data Fig. 4c) and (6) mTORC1 activity still responds properly to exogenous AA availability (Figs. 1–3, 6 and 7 and Extended Data Figs. 2, 3 and 7–9) and to growth factor signalling (Extended Data Fig. 7d–f) in cells where these complexes are non-lysosomal. Therefore, we propose that the non-lysosomal activity of mTORC1 is physiologically relevant in cells and presumably also in animal tissues.

If Rag KO mice maintain active mTORC1, why are they embryonic lethal? As cells with Rag loss-of-function do not completely respond to AA removal^23^ and show compromised mTORC1 re-activation upon AA add-back^18,23^, one possibility is that this is due to improper response to AA starvation and resupplementation. Alternatively, because *Rag*-mutant cells have constitutively activated TFEB/TFE3 signalling, and because these TFs play important roles in the metabolic and physiological response to starvation in mice^87,88^, we speculate that the dysregulation of the TFEB/TFE3 branch is the most important reason why *Rag* removal leads to lethality in mouse embryos.

Previous work by the Zoncu laboratory indicated that depletion of the FLCN–FNIP2 complex (an upstream regulator of RagC) alters TFE3 localization, without affecting the phosphorylation of S6K and 4E-BP1 (ref. ^89^). Along the same lines, an independent study from the Ballabio group showed that the phosphorylation and localization of TFEB is specifically controlled by a FLCN–RagC/D complex in response to AAs, but does not require growth factor signalling to Rheb^90^. Interestingly, a more recent publication from the Henske laboratory described the disconnection of mTORC1 substrate phosphorylation downstream of TSC: while S6K phosphorylation increases in cells lacking TSC expression, TFEB/TFE3 are dephosphorylated and translocate to the nucleus^91^. Our findings, presented here, expand these previous observations and provide a mechanistic and cell-biological explanation for the substrate specificity downstream of mTORC1, which—we now show—is defined by the spatial separation of mTORC1 activities both at the lysosomes and away from them. Our data are also in agreement with previous work in yeast by the De Virgilio group, suggesting that this phenomenon is evolutionarily conserved^92^. Importantly, together with these studies, our findings underscore the need to redefine the term ‘mTORC1 activity’ in the first place: stating that mTORC1 is generally ‘active’ or ‘inactive’ is clearly not sufficient, and one would need to specify whether mTORC1 is active or inactive towards a particular set of targets.

As we find that (1) under basal conditions, non-lysosomal mTORC1 is regulated primarily by exogenous AAs, while lysosomal mTORC1 mostly responds to AAs coming from the lysosomal lumen, (2) cells with non-lysosomal mTORC1 are sensitized to removal of different groups of exogenous AAs and (3) AAs signal to non-lysosomal mTORC1 independently of the lysosomal Rag-related machinery or the known cytoplasmic AA sensors, we propose that additional, currently unidentified proteins and pathways mediate AA signalling to mTORC1 away from lysosomes. As with the intricate, Rag-associated signalling network that has been put together over the last 15 years, we envisage that a similarly complex AA-sensing machinery remains to be identified in the years to come, to complete the picture of how cell growth, metabolism and nutrient sensing are coordinated in cells.

## Methods

### Cell culture

All cell lines were grown at 37 °C, 5% CO_2_. Human female embryonic kidney HEK293FT cells (#R70007, Invitrogen; RRID: CVCL_6911), immortalized MEFs and human male colorectal cancer SW-620 cells (#CCL-227, American Type Culture Collection (ATCC); RRID: CVCL_0547) were cultured in high-glucose Dulbecco’s modified Eagle medium (DMEM) (#41965039, Gibco), supplemented with 10% fetal bovine serum (FBS) (#F7524, Sigma; #S1810, Biowest). Human male diploid lung WI-26 SV40 fibroblasts (WI-26 cells; CCL-95.1, ATCC; RRID: CVCL_2758) were cultured in DMEM/F12 GlutaMAX medium (#31331093, Gibco) containing 10% FBS. All media were supplemented with 1× penicillin–streptomycin (15140-122, Gibco).

HEK293FT cells were purchased from Invitrogen. Wild-type (WT) control and RagA/B KO immortalized MEFs were a kind gift of Kun-Liang Guan (described in ref. ^51^). SW-620 cells were obtained from ATCC before the initiation of the project. The identity of the WI-26 cells was validated using the short tandem repeat profiling service, provided by Multiplexion GmbH. The identity of the HEK293FT cells was validated by the multiplex human cell line authentication test (Multiplexion GmbH), which uses a single nucleotide polymorphism typing approach, and was performed as described at www.multiplexion.de. No commonly misidentified cell lines were used in this study. All cell lines were regularly tested for *Mycoplasma* contamination, using a PCR-based approach and were confirmed to be *Mycoplasma* free.

### Cell culture treatments

AA starvation experiments were performed as described previously^23,39^. In brief, custom-made starvation media were formulated according to the Gibco recipe for high-glucose DMEM, specifically omitting all or individual AAs or specific AA groups, as indicated in the figures. The media were filtered through a 0.22 μm filter device and tested for proper pH and osmolality before use. For the respective AA-replete (+AA) treatment media, commercially available high-glucose DMEM was used (#41965039, Thermo Fisher Scientific). All treatment media were supplemented with 10% dialysed FBS (dFBS) and 1× penicillin–streptomycin (#15140-122, Gibco). For this purpose, FBS was dialysed against 1× PBS through 3,500 MWCO dialysis tubing. For basal (+AA) conditions, the culture media were replaced by +AA treatment media 60–90 min before lysis or fixation. For AA starvation (−AA), culture media were replaced by starvation media for 1 h, unless otherwise indicated in the figure legends. For AA add-back experiments, cells were first starved as described above and then starvation media were replaced by +AA treatment media for 10 or 30 min, unless otherwise indicated in the figures.

For growth factor starvation experiments, complete culture media were replaced by FBS-free DMEM supplemented with 1× penicillin–streptomycin (#15140-122, Gibco) for 1 h. For growth factor re-addition, cells were first starved for growth factors as described above and then 1 μM insulin (#I9278, Sigma) was added to the media for 30 min before collection. For glucose starvation experiments, cells were cultured for 1 h in glucose-free DMEM (#11966025, Gibco) supplemented with 10% dFBS and 1× penicillin–streptomycin. For the respective control wells, the culture media were replaced by high-glucose DMEM containing 10% dFBS and 1× penicillin–streptomycin at the beginning of the experiment. For glucose re-addition samples, cells were first starved for glucose as described above and media were then replaced by glucose-containing media for another 30 min before collection.

For bafilomycin A1 (#BML-CM110-0100, Enzo) treatments, the drug was added to a final concentration of 100 nM in the media for 6 h before lysis or fixation, unless otherwise indicated in the figure legends. For concanamycin A (ConA) (#C9705, Sigma) treatment, the drug was added to a final concentration of 100 nM in the media for 6 h before lysis or fixation. Chloroquine (CQ) (#C6628, Sigma) was added to the media to a final concentration of 50 μM for 6 h before lysis or fixation. Treatment with E64 (#2935.1, Roth) and PepA (#2936.1, Roth) to block lysosomal protease activity was performed by adding a combination of E64 (25 μM) and PepA (50 μM) in the media for 16 h before lysis or fixation. For experiments including treatments with +AA and −AA media, BafA1, ConA, CQ or E64 + PepA were kept also in the treatment media. For all experiments that did not include treatments with starvation media, the culture media were refreshed 90 min before lysis, also including fresh inhibitors. To inhibit mTOR kinase activity, Torin1 (#14379, Cell Signaling Technology) was added in the culture media (final concentration 250 nM) for 1 h (HEK293FT experiments) or 2 h (WI-26 experiments). Specific mTORC1 inhibition was performed by adding rapamycin (#S1039, Selleckchem) directly to the culture media (final concentration 20 nM) for the times indicated in the figure panels. Akt inhibition was achieved by addition of the Akt inhibitor VIII (#ENZ-CHM125, Enzo) in the culture media for 30 min (final concentration of 10 µM). Golgicide A (#345862, Sigma) and brefeldin A (#BUF075, Bio-Rad) were added in the culture media at final concentrations of 10 µM and 10 µg ml^−1^, respectively, for 1 h. For all drug treatments, DMSO (#4720.1, Roth) was used as control.

### Antibodies

A list of all primary antibodies used in this study can be found in Supplementary Table 1.

The H4B4 and ABL-93 antibodies against LAMP2 were obtained from the Developmental Studies Hybridoma Bank (DSHB), created by the National Institute of Child Health and Human Development (NICHD) of the National Institutes of Health (NIH) and maintained at The University of Iowa, Department of Biology. H4B4 was deposited to the DSHB by August, J.T./Hildreth, J.E.K. (DSHB Hybridoma Product H4B4)^93^. ABL-93 was deposited to the DSHB by August, J.T. (DSHB Hybridoma Product ABL-93)^94^.

### Plasmids and molecular cloning

The pETM-11-4E-BP1 vector, used to express His_6_-tagged 4E-BP1 in bacteria, was generated by PCR amplifying human 4E-BP1 from complementary DNA (cDNA) (prepared from HEK293FT cells) using appropriate primers and cloned in the NcoI-NotI restriction sites of pETM-11. For the GRASP55-myc expression vectors (pITR-TTP-GRASP55-myc-His), C-terminally myc-and His-tagged GRASP55 (WT and T264A mutant) was PCR amplified from the respective pcDNA4/TO/hGRASP55-myc-His plasmids (described in refs. ^36,95^) and cloned into the into the sleeping-beauty-based, doxycycline-inducible pITR-TTP vector^96^ using the SfiI/NotI restriction sites. The pcDNA3-Flag-hRagA WT and pcDNA3-Flag-hRagA Q66L constructs were described previously^23^. The pSpCas9(BB)-2A-Puro (PX459) V2.0 plasmid was purchased from Addgene (plasmid #62988; deposited by Feng Zhang) and described in ref. ^97^. The pLJC6-3xHA-TMEM192 and pLJC6-2xFlag-TMEM192 plasmids^98^ were purchased from Addgene (plasmids #104434 and #104435; deposited by the Sabatini laboratory). All restriction enzymes were purchased from Fermentas/Thermo Scientific. The integrity of all constructs was verified by sequencing. All DNA oligonucleotides used in this study are listed in Supplementary Table 1.

### mRNA isolation, cDNA synthesis and quantitative real-time PCR

Total messenger RNA was isolated from cells using a standard TRIzol/chloroform-based method (#15596018, Thermo Fisher Scientific), according to the manufacturer’s instructions. For cDNA synthesis, mRNA was transcribed to cDNA using the RevertAid H Minus Reverse Transcriptase kit (#EP0451, Thermo Fisher Scientific) according to the manufacturer’s instructions. The cDNAs were diluted 1:10 in nuclease-free water and 4 µl of diluted cDNA were used per reaction, together with 5 µl 2× Maxima SYBR Green/ROX qPCR master mix (#K0223, Thermo Fisher Scientific) and 1 µl primer mix (2.5 µM of forward and reverse primers). Reactions were set in technical triplicates in a StepOnePlus Real-Time PCR system (Applied Biosystems). Relative gene expression was calculated with the 2^−ΔΔCt^ method, with *RPL13a* as an internal control and normalized to the expression of the gene in the respective siCtrl or WT sample. All qPCR primers used in this study are listed in Supplementary Table 1.

### Plasmid DNA transfections

Plasmid DNA transfections in HEK293FT cells were performed using Effectene transfection reagent (#301425, Qiagen), according to the manufacturer’s instructions. For the reconstitution of GRASP55 KO WI-26 cells, plasmid DNA transfections were performed using the X-tremeGENE HP DNA transfection reagent (#06366236001, Roche) in a 2:1 DNA/transfection reagent ratio according to the manufacturer’s protocol.

### Generation of stable cell lines

For the generation of stable cell lines expressing HA-tagged TMEM192 (lyso-IP lines) or FLAG-tagged TMEM192 (negative control lines for anti-HA lyso-IPs), WT HEK293FT cells were transfected using the respective expression vectors. At 48 h post transfection, cells were selected with 3 μg ml^−1^ puromycin (#A11138-03, Thermo Fisher Scientific). Single-cell clones that express similar TMEM192 levels were used in lyso-IP experiments.

Stable cell lines expressing WT or T264A mutant GRASP55 were generated by reconstituting GRASP55 KO WI-26 cells using a doxycycline-inducible sleeping-beauty-based transposon system^96^ as described previously^36^. In brief, cells were transfected with the transposon-flanked pITR-TTP-GRASP55-myc-His plasmids (WT and T264A GRASP55) described above, together with the transposase expressing pCMV-Trp vector. At 24 h post transfection, puromycin (2 μg ml^−1^) was added to the medium and cells were selected for 5 days. Single-cell colonies were picked using cloning cylinders (#CLS31668, Sigma-Aldrich) and expanded. Clones that express similar levels of WT and T264 GRASP55 (leaky expression in the absence of doxycycline) were used in follow-up experiments.

### Generation of KO cell lines

The GRASP55 KO WI-26 cells were described previously^36^. The HEK293FT RagA/B ΚΟ, RagC/D ΚΟ, GNPTAB KO, SW-620 RagA/B ΚΟ, HEK293FT HA-TMEM192 RagA/B KO and FLAG-TMEM192 RagA/B KO cell lines were generated using the pX459-based CRISPR–Cas9 method, as described elsewhere^97^. The single guide RNA (sgRNA) expression vectors were generated by cloning appropriate DNA oligonucleotides (Supplementary Table 1) into the BbsI restriction sites of the pX459 vector (#62988, Addgene). An empty pX459 vector was used to generate matching control cell lines. In brief, transfected cells were selected with 3 μg ml^−1^ puromycin (#A11138-03, Thermo Fisher Scientific) 48 h post transfection. Single-cell clones were generated by single-cell dilution or single-cell sorting and KO clones were validated by immunoblotting and functional assays.

### Gene silencing experiments

Transient knockdown of *GNPTAB*, *LAMTOR1*, *TSC2*, *MIOS* and *ARF1*, were performed using siGENOME (pool of four) gene-specific short interfering RNAs (siRNAs) (Horizon Discoveries). An siRNA duplex targeting the *Renilla reniformis* luciferase gene (RLuc) (#P-002070-01-50, Horizon Discoveries) was used as control. Transfections were performed using 20 nM siRNA and the Lipofectamine RNAiMAX transfection reagent (#13778075, Thermo Fisher Scientific), according to the manufacturer’s instructions. Cells were collected or fixed 72 h post transfection and knockdown efficiency was verified by immunoblotting or quantitative real-time PCR.

### Cell lysis and immunoblotting

For standard sodium dodecyl sulfate–polyacrylamide gel electrophoresis (SDS–PAGE) and immunoblotting experiments, cells from a well of a 12-well plate were treated as indicated in the figures, washed once with serum-free DMEM and lysed in 250 μl of ice-cold Triton lysis buffer (50 mM Tris pH 7.5, 1% Triton X-100, 150 mM NaCl, 50 mM NaF, 2 mM Na-vanadate and 0.011 g ml^−1^ beta-glycerophosphate), supplemented with 1× PhosSTOP phosphatase inhibitors (#04906837001, Roche) and 1× cOmplete protease inhibitors (#11836153001, Roche) for 10 min on ice. Samples were clarified by centrifugation (19,000*g*, 15 min, 4 °C) and supernatants transferred to a new tube. Protein concentration was determined using a Protein Assay Dye Reagent (#5000006, Bio-Rad). Normalized samples were boiled in 1× SDS sample buffer for 5 min at 95 °C (6× SDS sample buffer: 350 mM Tris–HCl pH 6.8, 30% glycerol, 600 mM dithiothreitol, 12.8% SDS and 0.12% bromophenol blue). For WI-26 lysates, cells from a well of a 6-well plate were treated as indicated in the figures and lysed in well with 300 µl of ice-cold WI-26 lysis buffer (50 mM Tris–HCl pH 7.5, 0.5% Triton X-100, 150 mM NaCl and 0.1% SDS), supplemented with 1× PhosSTOP phosphatase inhibitors (#04906837001, Roche) and 1× cOmplete protease inhibitors (#11836153001, Roche). Samples were clarified by centrifugation (12,000*g*, 15 min, 4 °C) and supernatants transferred to a new tube. Samples were boiled in 1× SDS sample buffer for 5 min at 95 °C.

For protein secretion experiments, WT or GNPTAB KO HEK293FT cells were cultured in serum-free media for 16 h. Supernatants were collected and centrifuged (2,000*g*, 5 min, 4 °C) to remove dead cells and debris. Cleared supernatants were concentrated using 3 kDa cut-off concentrator tubes (#516-0227 P, VWR), according to the manufacturer’s instructions. SDS sample buffer (1×) was added to the concentrated supernatants and samples were boiled for 5 min at 95 °C before loading into SDS–PAGE gels.

Protein samples were subjected to electrophoretic separation on SDS–PAGE and analysed by standard western blotting techniques. In brief, proteins were transferred to nitrocellulose membranes (#10600002 or #10600001, Amersham) and stained with 0.2% ponceau solution (#33427-01, Serva) to confirm equal loading. Membranes were blocked with 5% skim milk powder (#42590, Serva) in PBS-T (1× PBS and 0.1% Tween-20 (#A1389, AppliChem)) for 1 h at room temperature, washed three times for 10 min with PBS-T and incubated with primary antibodies (1:1,000 in PBS-T, 5% bovine serum albumin (BSA; #10735086001, Roche)) rotating overnight at 4 °C. The next day, membranes were washed three times for 10 min with PBS-T and incubated with appropriate horseradish peroxidase (HRP)-conjugated secondary antibodies (1:10,000 in PBS-T, 5% milk) for 1 h at room temperature. For the experiments using WI-26 samples or HEK293FT samples to assay GRASP55 phosphorylation upon BafA1 treatment, proteins were transferred to polyvinylidene difluoride (PVDF) membranes (#10600023, Amersham). Equal loading was confirmed by staining with ponceau S, blocked with 5% skim milk powder (#42590, Serva) in TBS-T buffer (50 mM Tris–HCl pH 7.4, 150 mM NaCl and 0.1% Tween-20) and incubated with primary antibodies diluted in TBS-T, for 1 h at room temperature or overnight at 4 °C, followed by incubation with appropriate HRP-conjugated secondary antibodies (1:10,000 in TBS-T) for 1 h at room temperature. Signals were detected by enhanced chemiluminescence (ECL), using the ECL Western Blotting Substrate (#W1015, Promega) or SuperSignal West Pico PLUS (#34577, Thermo Scientific) and SuperSignal West Femto Substrate (#34095, Thermo Scientific) for weaker signals. Immunoblot images were captured on films (#28906835, GE Healthcare; #4741019289, Fujifilm). Blots were quantified using GelAnalyzer 19.1.

### Lambda-phosphatase treatment assays

Lambda-phosphatase treatment experiments were performed as one of the ways to validate the specificity of the custom-made phospho-specific antibody recognizing GRASP55 phosphorylated on T264. In brief, cells were lysed in 300 μl ice-cold WI-26 lysis buffer (50 mM Tris–HCl pH 7.5, 0.5% Triton X-100, 150 mM NaCl and 0.1% SDS), supplemented with 1× EDTA-free cOmplete protease inhibitors (#11873580001, Roche), as described above. Lysates were cleared by centrifugation (15 min, 12,000*g*) and 100 units of λ-phosphatase (#P0753, New England Biolabs) were added to the supernatants, followed by 30 min incubation at 30 °C. SDS sample buffer (1× final concentration) was added to the reactions, samples were boiled for 5 min at 95 °C and analysed by immunoblotting as described above.

### Lysosome purification (lyso-IP) assays

To biochemically isolate intact lysosomes and associated proteins, we developed a modified lyso-IP method, based on the protocol previously described by the Sabatini group^31^, which allowed us to also assess the non-lysosomal fractions. In brief, cells were seeded on a 15 cm dish until they reached 80–90% confluency, washed 2× with ice-cold PBS and scraped in 1 ml ice-cold PBS, containing 1× PhosSTOP phosphatase inhibitors (#04906837001, Roche) and 1× cOmplete protease inhibitors (#11697498001, Roche). Cells were then pelleted by centrifugation (1,000*g*, 2 min, 4 °C) and resuspended in 1 ml ice-cold PBS containing phosphatase and protease inhibitors. For input samples, 25 μl of the cell suspension were transferred in a new tube and lysed by the addition of 125 μl of Triton lysis buffer (50 mM Tris pH 7.5, 1% Triton X-100, 150 mM NaCl, 50 mM NaF, 2 mM Na-vanadate and 0.011 g ml^−1^ beta-glycerophosphate), supplemented with 1× PhosSTOP phosphatase inhibitors (#04906837001, Roche) and 1× cOmplete protease inhibitors (#11836153001, Roche) on ice for 10 min. Lysed input samples were then cleared by centrifugation (14,000*g*, 15 min, 4 °C), and the supernatant was transferred to new tubes containing 37.5 μl of 6× SDS sample buffer and boiled for 5 min at 95 °C. For the lysosomal and non-lysosomal fractions, the remaining cell suspension was homogenized with 20 strokes in pre-chilled 2 ml hand dounce homogenizers kept on ice. The homogenate was cleared by centrifugation to remove unbroken cells (1,000*g*, 2 min, 4 °C) and the supernatant was incubated with 100 μl pre-washed Pierce anti-HA magnetic beads (#88837, Thermo Fisher Scientific) on a nutating mixer for 3 min at room temperature. After incubation with the beads, the supernatant was transferred to a new tube and centrifuged at high speed (20,000*g*, 10 min, 4 °C) to remove membranes and other organelles and retrieve the non-lysosomal/cytoplasmic fraction. Then, 25 µl of the cleared supernatant was transferred in a new tube, mixed with 125 µl Triton lysis buffer and incubated for 10 min on ice. Next, 37.5 µl 6× SDS sample buffer was added and samples were boiled. For the lysosomal fraction, beads were washed three times with ice-cold PBS containing phosphatase and protease inhibitors using a DynaMag spin magnet (#12320D, Invitrogen). After the last wash, lysosomes were eluted from the beads by addition of 50 µl Triton lysis buffer and incubation for 10 min on ice. Isolated lysosomes were then transferred to a new tube, 12.5 µl 6× SDS sample buffer was added and samples were boiled.

### Production of recombinant His_6_-tagged 4E-BP1 protein in bacteria

Recombinant His_6_-tagged 4E-BP1 protein was produced by transforming *E.* *coli* BL21 RP electrocompetent bacteria with the pETM-11-4E-BP1 vector described above, according to standard procedures. In brief, protein expression was induced with isopropyl-β-d-thiogalactopyranoside (IPTG) for 3 h at 30 °C, and His_6_-4E-BP1 was purified using Ni-NTA agarose (#1018244, Qiagen) and eluted with 250 mM imidazole (#A1073, Applichem).

### mTORC1 kinase activity assays

In vitro mTORC1 kinase assays were developed based on previous reports^99–101^, using endogenous mTORC1 complexes immunopurified from HEK293FT WT or RagA/B KO cells. In brief, cells of a near-confluent 10 cm dish were lysed in ice-cold CHAPS IP buffer (50 mM Tris pH 7.5, 0.3% CHAPS, 150 mM NaCl, 50 mM NaF, 2 mM Na-vanadate and 0.011 g ml^−1^ beta-glycerophosphate), supplemented with 1× PhosSTOP phosphatase inhibitors (#04906837001, Roche) and 1× cOmplete protease inhibitors (#11836153001, Roche) for 10 min on ice. Samples were clarified by centrifugation (19,000*g*, 15 min, 4 °C), supernatants were collected and a portion was kept as input material. The remaining supernatants were subjected to immunoprecipitation by incubation with 2 μl of anti-mTOR antibody (#2983, Cell Signaling Technology) for 3 h (4 °C, rotating), followed by incubation with 30 μl of pre-washed Protein A agarose bead slurry (#11134515001, Roche) for an additional hour (4 °C, rotating). Beads were then washed four times with ice-cold CHAPS IP wash buffer (50 mM Tris pH 7.5, 0.3% CHAPS, 150 mM NaCl and 50 mM NaF) and once with kinase wash buffer (25 mM HEPES pH 7.4 and 20 mM KCl) and excess liquid was removed with a Hamilton syringe. Kinase reactions were prepared by adding 10 μl 3× kinase assay buffer (75 mM HEPES/KOH pH 7.4, 60 mM KCl and 30 mM MgCl_2_) to the beads. Reactions were started by adding 10 μl of kinase assay start buffer (25 mM HEPES/KOH pH 7.4, 140 mM KCl and 10 mM MgCl_2_), supplemented with 500 μM ATP and 35 ng recombinant His_6_-4E-BP1 substrate. Reactions lacking ATP were set up as negative controls. All reactions were incubated at 30 °C for 30 min, and stopped by the addition of one volume 2× SDS sample buffer and boiling for 5 min at 95 °C. Samples were run on SDS–PAGE and the mTORC1-mediated phosphorylation on 4E-BP1^T37/46^ was detected by immunoblotting with a specific antibody (#9459, Cell Signaling Technology).

### Immunofluorescence and confocal microscopy

Immunofluorescence/confocal microscopy experiments were performed as described previously^23,102^. In brief, cells were seeded on glass coverslips coated with fibronectin (#A8350, Applichem) or gelatin (#G1393, Sigma), treated as described in the figure legends, and fixed with 4% paraformaldehyde (PFA) in 1× PBS (10 min, room temperature), followed by two permeabilization/washing steps with PBT (1× PBS and 0.1 % Tween-20). Cells were blocked in BBT (1× PBS, 0.1% Tween-20 and 1% BSA) for 45 min. Staining with anti-mTOR (#2983, Cell Signaling Technology), anti-RagC (#9480, Cell Signaling Technology), anti-LAMP2 (#H4B4 for human cells; #ABL-93 for mouse cells; both from Developmental Studies Hybridoma Bank) and anti-GM130 (#610822, BD Transduction Laboratories) primary antibodies diluted 1:200 in BBT solution was performed for 2 h at room temperature. Staining with anti-TFEB (#4240, Cell Signaling Technology) or anti-TFE3 (#14779, Cell Signaling Technology) antibodies (1:200 in BBT) was performed by incubation for 16 h at 4 °C. After staining with primary antibodies, cells were washed three times with PBT. Next, cells were stained with highly cross-adsorbed fluorescent secondary antibodies (donkey anti-rabbit Alexa Fluor 488-conjugated, for all rabbit primary antibodies; donkey anti-mouse Alexa Fluor 594-conjugated for anti-GM130; donkey anti-mouse TRITC-conjugated, for all other mouse primary antibodies; all from Jackson ImmunoResearch) diluted 1:200 in BBT for 1 h. Nuclei were stained with 4,6-diamidino-2-phenylindole (DAPI) (#A1001, VWR) (1:2,000 in PBT) for 5 min and coverslips were washed three times with PBT solution before mounting on glass slides with Fluoromount-G (#00-4958-02, Invitrogen).

For LC3B or p62 staining, cells were fixed with 100% methanol for 15 min at −20 °C, permeabilized with 0.1% Triton X-100 (#A4975, AppliChem) for 5 min and blocked for 1 h in LC3B blocking solution (1× PBS, 5% FBS and 0.3% Triton X-100). Coverslips were incubated overnight at 4 °C with anti-LC3B (#3868, Cell Signaling Technology) or anti-p62 (#PM045, MBL) antibodies in LC3B/p62 staining solution (1× PBS, 1% BSA and 0.3% Triton X-100). Slides were washed three times in 1× PBS, incubated with donkey anti-rabbit Alexa Fluor 488 (Jackson ImmunoResearch) (1:500, in 1× PBS, 1% BSA and 0.3% Triton X-100) for 1 h at room temperature. Coverslips were then washed twice with 1× PBS, stained with DAPI (1:2,000 in 1× PBS) and mounted on glass slides with Fluoromount-G (#00-4958-02, Invitrogen). All images were captured on an SP8 Leica confocal microscope (TCS SP8 X or TCS SP8 DLS, Leica Microsystems) using a 40× oil objective lens. Image acquisition was performed using the LAS X software (Leica Microsystems). Images from single channels are shown in greyscale, whereas in merged images, Alexa Fluor 488 is shown in green, Alexa Fluor 594 and TRITC in red and DAPI in blue.

### LysoTracker staining

For LysoTracker staining experiments, cells were seeded in fibronectin-coated coverslips and grown until they reached 80–90% confluency. Lysosomes were stained by the addition of 100 nM LysoTracker Red DND-99 (#L7528, Invitrogen) in complete medium for 1 h in standard culturing conditions. Cells were then fixed with 4% PFA in PBS for 10 min at room temperature, washed and permeabilized with PBT solution (1× PBS and 0.1% Tween-20), and nuclei stained with DAPI (1:2,000 in PBT) for 10 min. Coverslips were mounted on slides using Fluoromount-G (#00-4958-02, Invitrogen). All images were captured on an SP8 Leica confocal microscope (TCS SP8 X or TCS SP8 DLS, Leica Microsystems) using a 40× oil objective lens. Image acquisition was performed using the LAS X software (Leica Microsystems).

### Quantification of colocalization

Colocalization analysis in confocal microscopy experiments was performed as in refs. ^39,102^, using the Coloc2 plugin of the Fiji software^103^. An average of 50 individual cells from three to five independent representative images per condition captured from one representative experiment (out of two to three independent replicate experiments as indicated in the figure legends) was used to calculate Manders’ colocalization coefficient with automatic Costes thresholding^104–106^. For experiments in which lysosomal size and morphology are affected (for instance, in BafA1-treated or GNPTAB KO cells), thus also influencing lysosomal signal distribution or intensity, Pearson’s correlation coefficient was used instead^106^. The area corresponding to the cell nucleus was excluded from the cell region of interest (ROI) to prevent false-positive colocalization due to automatic signal adjustments. Manders’ colocalization coefficient and Pearson’s correlation coefficient are defined as a part of the signal of interest (mTOR or RagC), which overlaps with a second signal (LAMP2).

### Quantification of LC3B, p62 and LysoTracker intensities

Signal intensity was calculated using the Fiji software. ROIs were determined for approximately 50 cells over 5 independent representative images per condition and integrated density was calculated, representing the sum of the values of all pixels in the given ROI. Exact numbers of individual cells analysed per experiment are indicated in the figure legends.

### Scoring of TFEB/TFE3 localization

Subcellular localization of TFEB and TFE3 was performed by scoring the distribution of signal in the cytoplasm and the nucleus. Five independent fields per condition were analysed for each experiment. The exact numbers of individual cells analysed per experiment are indicated in the figure legends.

### Immunoelectron microscopy

Immuno-EM experiments were performed by Colzyx AB (www.colzyx.com). In brief, WT or RagA/B KO MEF cells were treated as described in the figure legends and collected by centrifugation (5 min, 250*g*). Each sample (5 × 10^6^ cells) was gently transferred into a 1 ml sample tube and centrifuged at 2,500*g* for 20 min. Pellets were fixed in 2.5% glutaraldehyde in 0.1 M sodium cacodylate (pH 7.2) for 2 h at 4 °C and subsequently washed with 0.15 M cacodylate (pH 7.2). Samples were then post-fixed with 1% osmium tetroxide and 0.15 M sodium cacodylate (pH 7.2) for 1 h at 4 °C, washed 3× with 1 ml 0.15 M sodium cacodylate (pH 7.2), and further processed for transmission electron microscopy according to standard protocols. Briefly, the fixed and washed samples were dehydrated in ethanol and further processed for routine Epon embedding. Sections were cut with an LKB ultratome equipped with a diamond knife and mounted on Formvar-coated nickel grids.

Before immunostaining, grids were subjected to antigen unmasking with sodium metaperiodate as described previously^107^. Grids were incubated in a humidified chamber on 100 μl drops of a saturated sodium metaperiodate aqueous solution for 1 h at room temperature. For immunostaining, the grids were floated on 100 μl drops of immune reagents displayed on a sheet of Parafilm in a humidified chamber. Free aldehyde groups were blocked with 50 mM glycine, and the grids were then incubated with 5% donkey serum (ab7475, Abcam) in incubation buffer (0.2% BSA-c (#900.022, Aurion) in PBS, pH 7.6) for 15 min. The blocking procedure was followed by overnight incubation with the following primary antibodies at 4 °C: rabbit monoclonal anti-mTOR antibody against mTOR (#2983, CST; dilution 1:80) or rat monoclonal antibody against mouse LAMP2 (#ABL-93, Developmental Studies Hybridoma Bank; dilution 1:80). After washing the grids in a large volume (200 ml) of incubation buffer, staining with the gold particle-conjugated antibodies (10 nm size for mTOR and 5 nm size for LAMP2) was performed by floating the grids on drops containing the gold conjugate reagents (diluted 1:20 in incubation buffer) for 60 min at room temperature. After additional washes (3× in 50 ml incubation buffer each), the sections were post-fixed in 2% glutaraldehyde. Finally, sections were washed with distilled water and post-stained with uranyl acetate and lead citrate. Specimens were observed in a Philips/FEI CM100 transmission electron microscope (Philips/FEI) operated at 80 kV accelerating voltage and images were recorded with a side-mounted Olympus Veleta camera (Olympus) with a resolution of 2,048 × 2,048 pixels.

For quantifications, values are shown as number of 10 nm gold particles per square micrometer. Localization of gold particles to lysosomes or the cytoplasm was assessed based on morphological criteria and LAMP2 staining. Twenty randomly selected areas were evaluated per sample from three independently processed ultrathin sections (a total of 58–60 cellular profiles) per experimental condition.

### OPP assay

To test de novo protein synthesis, OPP incorporation assays were performed using the Click-iT Plus OPP Protein Synthesis Assay kit (#C10456, Thermo Fisher Scientific), according to the manufacturer’s instructions. In brief, cells were seeded in fibronectin-coated coverslips until they reached 80–90% confluence. Control samples were treated with 100 µM cycloheximide (#239765, Sigma) for 4 h before fixation to block translation. Click-iT OPP component A (20 µM) was added to the culture medium for 30 min, cells were fixed for 10 min at room temperature with 4% PFA and washed twice with PBT. Next, cells were incubated with Click-iT Plus OPP reaction cocktail for 30 min at room temperature protected from light, followed by one wash with Click-iT Reaction Rinse Buffer and further DAPI staining as described for immunofluorescence. All samples were imaged on an SP8 Leica confocal microscope (TCS SP8 X or TCS SP8 DLS, Leica Microsystems) using a 40× oil objective lens. Image acquisition was performed using the LAS X software (Leica Microsystems).

For cytometry-based detection of protein translation levels, 1 × 10^6^ cells were used per condition. Cells were incubated with 20 µM of Click-iT OPP component A for 1 h, collected and centrifuged for 3 min (200*g*) at room temperature. Samples were fixed with ice-cold 70% ethanol, incubated for 30 min on ice, washed once with 1× PBS, followed by two washes with 1× PBS, 0.3% BSA and permeabilization with 0.1% Saponin in 1× PBS, 0.3% BSA for 10 min at room temperature. Cells were then centrifuged for 3 min (200*g*) at room temperature, the supernatant was removed and cells were incubated with Click-iT Plus OPP reaction cocktail for 30 min. Next, cells were washed twice with 1× PBS, 0.3% BSA and Alexa Fluor 488 signal was detected with a FITC filter in a BD LSR Fortessa cell analyser flow cytometer (BD Biosciences) and further analysed with the FlowJo v10 software (TreeStar).

### Gene expression analysis

To assess mRNA expression of selected *SREBP*, *HIF1*, *ATF4* and *TFEB* target genes, we used two RNA-seq datasets: from rapamycin- or DMSO-treated HEK293FT cells (described in ref. ^60^; National Center for Biotechnology Information (NCBI) Sequence Read Archive PRJNA872474), and from RagA/B KO versus control HEK293FT cells (NCBI Sequence Read Archive PRJNA1131360). Target genes of each transcription factor (TF) were retrieved using the Harmonizome 3.0 online platform (https://maayanlab.cloud/Harmonizome/)^108^. For ATF4 targets, a list of non-redundant genes was assembled using the ATF4 gene set from Harmonizome 3.0 and previous literature^109,110^. Genes for follow-up analyses were selected following manual validation in the literature. Dot plots showing the changes in the expression of the selected target genes in each of the two RNA-seq datasets for each TF were generated using the Scatter Plot tool of the Flaski toolbox^111^ (https://flaski.age.mpg.de, developed and provided by the MPI-AGE Bioinformatics core facility). For each dot, colour and size indicate log_2_-transformed fold change (log_2_FC) and outline colour indicates significance (black: adjusted *P* value <0.05; grey: adjusted *P* value ≥0.05).

### Statistics and reproducibility

Statistical analysis and presentation of quantification data was performed using GraphPad Prism (versions 9.1.0 and 9.2.0). Data in graphs are shown as mean ± s.e.m. The normality of data distribution was tested using the Shapiro–Wilk and Kolmogorov–Smirnov tests in Prism. For graphs with only two conditions shown and normal data distribution (Figs. 2c,3h and 8b,i and Extended Data Figs. 2e and 6d), significance for pairwise comparisons was calculated using Student’s *t*-tests. For graphs with only two conditions shown and non-normal data distribution (Figs. 1c, 6j and 8k and Extended Data Figs. 1c,e, 2b and 10f), significance for pairwise comparisons was calculated using Mann–Whitney *U* tests. For graphs with three or more conditions shown (Figs. 1f, 3c, 4b,d,e,g, 5c,f,g and 8g and Extended Data Figs. 3b,e and 8c), significance for pairwise comparisons to the respective controls was calculated using one-way analysis of variance (ANOVA) with post hoc Holm–Sidak test. For experiments starving cells for various AA groups (Fig. 7b,d,f), significance for pairwise comparisons to the respective controls was calculated using one-way ANOVA with post hoc Dunnett’s test. Sample sizes (*n*) and significance values are indicated in figure legends (*P < 0.05, **P < 0.01, ***P < 0.001 and ****P < 0.0001. n.s., non-significant). For Fig. 3c, *n*_siCtrl(+AA)_ = 46, *n*_siGNPTAB(+AA)_ = 44, *n*_siCtrl(–AA)_ = 47, *n*_siGNPTAB(–AA)_ = 50, *n*_siCtrl(–/+AA 10’)_ = 48, *n*_siGNPTAB(–/+AA 10’)_ = 50, *n*_siCtrl(–/+AA 30’)_ = 46, *n*_siGNPTAB(–/+AA 30’)_ = 50 individual cells from 5 independent fields per condition. For Fig. 4b, *n* = 50 for all conditions, except for *n*_BafA1(8h)_ = 49 individual cells from 5 independent fields per condition. For Fig. 5c, *n*_WT(+AA)_ = 56, *n*_KO(+AA)_ = 55, *n*_WT(−AA)_ = 60, *n*_KO(−AA)_ = 60, *n*_WT(–/+AA)_ = 55, *n*_KO(–/+AA)_ = 60 individual cells from 3–4 independent fields per condition. For Fig. 5f, *n* = 60 for all conditions, except for *n*_Cyto-mTOR(+AA)_ = 58 randomly selected areas (1 μm^2^ each) from three independent grids per condition.

All findings were reproducible over multiple independent experiments, within a reasonable degree of variability between replicates. For all main figure panels, data shown are representative of three independent replicate experiments (Figs. 1–5, 6a,b,d–j, 7e,f and 8a–g,j–k), unless otherwise specified in the figure legends (Figs. 6c, 7a–d and 8h,i). For extended data figure panels, the number of independent experiments is given directly in the figure legends. No statistical method was used to predetermine sample sizes, which were determined in accordance with standard practices in the field, as reported in previous publications^21,23,39,40,101,102^. No data were excluded from the analyses. The experiments were not randomized, and the investigators were not blinded to allocation during experiments and outcome assessment.

### Materials availability

All unique plasmids and cell lines generated in this study are available from the corresponding author on reasonable request, with a completed material transfer agreement.

### Reporting summary

Further information on research design is available in the Nature Portfolio Reporting Summary linked to this article.

## Online content

Any methods, additional references, Nature Portfolio reporting summaries, source data, extended data, supplementary information, acknowledgements, peer review information; details of author contributions and competing interests; and statements of data and code availability are available at 10.1038/s41556-024-01523-7.

## Supplementary information

Reporting Summary

Supplementary Table 1 Lists of primary antibodies (a) and DNA oligonucleotides (b) used in this study.

## Source data

Source Data Fig. 1 Unprocessed western blots for Fig. 1.

Source Data Fig. 2 Unprocessed western blots for Fig. 2.

Source Data Fig. 3 Unprocessed western blots for Fig. 3.

Source Data Fig. 4 Unprocessed western blots for Fig. 4.

Source Data Fig. 5 Unprocessed western blots for Fig. 5.

Source Data Fig. 6 Unprocessed western blots for Fig. 6.

Source Data Fig. 7 Unprocessed western blots for Fig. 7.

Source Data Extended Data Fig. 1 Unprocessed western blots for Extended Data Fig. 1.

Source Data Extended Data Fig. 2 Unprocessed western blots for Extended Data Fig. 2.

Source Data Extended Data Fig. 3 Unprocessed western blots for Extended Data Fig. 3.

Source Data Extended Data Fig. 4 Unprocessed western blots for Extended Data Fig. 4.

Source Data Extended Data Fig. 5 Unprocessed western blots for Extended Data Fig. 5.

Source Data Extended Data Fig. 6 Unprocessed western blots for Extended Data Fig. 6.

Source Data Extended Data Fig. 7 Unprocessed western blots for Extended Data Fig. 7.

Source Data Extended Data Fig. 8 Unprocessed western blots for Extended Data Fig. 8.

Source Data Extended Data Fig. 9 Unprocessed western blots for Extended Data Fig. 9.

Source Data Figs. 1–8 and Extended Data Figs. 1–3, 6 and 8–10 Numerical Source Data for Figs. 1-8 and Extended Data Figs. 1–3, 6 and 8–10.

## Data Availability

The RNA-seq dataset from rapamycin- or DMSO-treated HEK293FT cells (NCBI Sequence Read Archive PRJNA872474) was described previously in Artoni et al.^60^. The RNA-seq dataset from RagA/B KO versus control HEK293FT cells is deposited as NCBI Sequence Read Archive PRJNA1131360. All other data supporting the findings of this study are available from the corresponding author on reasonable request. Source data are provided with this paper.

## Code availability

No code was generated in this study.
