# Supplementary material for: Spatial and functional separation of mTORC1 signalling in response to different amino acid sources
Source: Nat Cell Biol. 2024 Oct 9;26(11):1918–33. doi: 10.1038/s41556-024-01523-7 (PMC11567901; doi:10.1038/s41556-024-01523-7)
Supplement: Supplementary file 1 — Reporting Summary [file 41556_2024_1523_MOESM1_ESM.pdf]

Corresponding author(s): Dr Constantinos Demetriades
Last updated by author(s): Aug 26, 2024

Reporting Summary

Nature Portfolio wishes to improve the reproducibility of the work that we publish. This form provides structure for consistency and transparency in reporting. For further information on Nature Portfolio policies, see our Editorial Policies and the Editorial Policy Checklist.

Statistics

For all statistical analyses, confirm that the following items are present in the figure legend, table legend, main text, or Methods section.

n/a	Confirmed	
☐	☒	The exact sample size (*n*) for each experimental group/condition, given as a discrete number and unit of measurement
☐	☒	A statement on whether measurements were taken from distinct samples or whether the same sample was measured repeatedly
☐	☒	The statistical test(s) used AND whether they are one- or two-sided<br>*Only common tests should be described solely by name; describe more complex techniques in the Methods section.*
☒	☐	A description of all covariates tested
☐	☒	A description of any assumptions or corrections, such as tests of normality and adjustment for multiple comparisons
☐	☒	A full description of the statistical parameters including central tendency (e.g. means) or other basic estimates (e.g. regression coefficient) AND variation (e.g. standard deviation) or associated estimates of uncertainty (e.g. confidence intervals)
☐	☒	For null hypothesis testing, the test statistic (e.g. *F*, *t*, *r*) with confidence intervals, effect sizes, degrees of freedom and *P* value noted<br>*Give P values as exact values whenever suitable.*
☒	☐	For Bayesian analysis, information on the choice of priors and Markov chain Monte Carlo settings
☒	☐	For hierarchical and complex designs, identification of the appropriate level for tests and full reporting of outcomes
☒	☐	Estimates of effect sizes (e.g. Cohen's *d*, Pearson's *r*), indicating how they were calculated

Our web collection on statistics for biologists contains articles on many of the points above.

Software and code

Policy information about availability of computer code

Data collection

No code for data collection was generated in this study.

Commercial and non-commercial software packages from other developers used in this study are:
Confocal microscopy: Leica Application Suite X 3.5.7.23225; https://www.leica-microsystems.com/products/microscope-software/p/leica-las-x-ls/
Immunoblot intensity measurements: GelAnalyzer 19.1; http://www.gelanalyzer.com/

Data analysis

No code for data analysis was generated in this study.

Commercial and non-commercial software packages from other developers used in this study are:
Statistical analysis and graph preparation: GraphPad Prism 9.1.0 and 9.2.0; https://www.graphpad.com/scientific-software/prism/
Quantification of colocalization: Fiji Version 2.0.0-rc-67/1.53c; https://imagej.net/software/fiji/
Quantification of immunoblots: GelAnalyzer 19.1; http://www.gelanalyzer.com/
Flow cytometry analysis for OPP assay: FlowJo (v10); https://www.flowjo.com/solutions/flowjo/downloads
Scatter plot data presentation from RNA-seq: Scatter Plot tool of the Flaski v.3.16.18 toolbox; https://flaski.age.mpg.de

For manuscripts utilizing custom algorithms or software that are central to the research but not yet described in published literature, software must be made available to editors and reviewers. We strongly encourage code deposition in a community repository (e.g. GitHub). See the Nature Portfolio guidelines for submitting code & software for further information.

Data

Policy information about availability of data

All manuscripts must include a data availability statement. This statement should provide the following information, where applicable:

- Accession codes, unique identifiers, or web links for publicly available datasets
- A description of any restrictions on data availability
- For clinical datasets or third party data, please ensure that the statement adheres to our policy

Source data have been provided in Source Data. The RNA-seq dataset from rapamycin- or DMSO-treated HEK293FT cells (NCBI Sequence Read Archive PRJNA872474) was described previously in Artoni et al. (PMID: 37222020). The RNA-seq dataset from RagA/B KO versus control HEK293FT cells is deposited as NCBI Sequence Read Archive PRJNA1131360. All other data supporting the findings of this study are available from the corresponding author on reasonable request.

Field-specific reporting

Please select the one below that is the best fit for your research. If you are not sure, read the appropriate sections before making your selection.

☒ Life sciences ☐ Behavioural & social sciences ☐ Ecological, evolutionary & environmental sciences

For a reference copy of the document with all sections, see nature.com/documents/nr-reporting-summary-flat.pdf

Life sciences study design

All studies must disclose on these points even when the disclosure is negative.

Sample size	No statistical methods were used for sample size determination, which was determined in accordance with standard practices in the field and based on our long-standing experience in this type of experimental approaches (e.g., PMIDs 26868506, 33497611, 33974911).<br>For all microscopy studies, an average of 50 individual cells from 3-5 independent representative images per condition captured from one representative experiment (out of 2-3 independent replicate experiments as indicated in the figure legends and in the 'Statistics and Reproducibility' section in Methods) are shown in dot plots. The number of individual cells quantified per condition in each panel is provided in the figure legends or in the 'Statistics and Reproducibility' section in Methods.
Data exclusions	No data were excluded from the analyses.
Replication	All findings were reproducible over multiple independent experiments, within a reasonable degree of variability between replicates. The exact numbers of replicate experiments are provided in the respective figure legends or in the 'Statistics and Reproducibility' section in Methods.
Randomization	No sample randomization was performed for the cell-culture-based assays described in this study, as the order of processing, analysis, and data collection does not influence the experimental outcomes. Experiments were performed based on standard practices in the field and our long-standing experience in this type of experimental approaches (e.g., PMIDs 26868506, 33497611, 33974911) to ensure unbiased data interpretation.
Blinding	The investigators were not blinded to the immunoblotting experiments as they need to rank and load the samples on gels in a particular order, hence they need to have detailed information about the identity of each sample. For immunofluorescence experiments, fields were selected based on one channel (e.g., DAPI or LAMP2) that is different from the channel used to assess protein localization (e.g., for mTOR).<br>No further blinding was included in data collection or analysis, as the method of quantification over multiple replicates for all experiments and using individual cells (i.e., for microscopy experiments) ensures unbiased processing.

Reporting for specific materials, systems and methods

We require information from authors about some types of materials, experimental systems and methods used in many studies. Here, indicate whether each material, system or method listed is relevant to your study. If you are not sure if a list item applies to your research, read the appropriate section before selecting a response.

Materials & experimental systems

n/a	Involved in the study	
☐	☒	Antibodies
☐	☒	Eukaryotic cell lines
☒	☐	Palaeontology and archaeology
☒	☐	Animals and other organisms
☒	☐	Human research participants
☒	☐	Clinical data
☒	☐	Dual use research of concern

Methods

n/a	Involved in the study	
☒	☐	ChIP-seq
☐	☒	Flow cytometry
☒	☐	MRI-based neuroimaging

Antibodies

Antibodies used

Primary antibodies
Rabbit, monoclonal, anti-phospho-S6K (Thr389), clone D5U10, #97596, Cell Signaling Technology, Lot #3
Rabbit, polyclonal, anti-S6K, #9202, Cell Signaling Technology, Lot #20
Rabbit, monoclonal, anti-phospho-TFEB (Ser211), clone E9S8N, #37681, Cell Signaling Technology, Lot #2
Rabbit, polyclonal, anti-TFEB, #4240, Cell Signaling Technology, Lot #3
Rabbit, polyclonal, anti-TFEB, #A303-673A, Bethyl Laboratories, Lot #8
Rabbit, polyclonal, anti-TFE3, #14779, discontinued, Cell Signaling Technology, Lot #1
Rabbit, polyclonal, anti-phospho-AKT (Ser473), #9271, Cell Signaling Technology, Lot #15
Rabbit, polyclonal, anti-AKT, #9272, Cell Signaling Technology, Lot #28
Rabbit, monoclonal, anti-phospho-4E-BP1 (Ser65), clone D9G1Q, #13443, Cell Signaling Technology, Lot #1
Rabbit, polyclonal, anti-phospho-4E-BP1 (Thr37/46), #9459, Cell Signaling Technology, Lot #10
Rabbit, polyclonal, anti-4E-BP1, #9452, Cell Signaling Technology, Lot #12
Rabbit, monoclonal, anti-phospho-S6 (Ser240/244), clone D68F8, #5364, Cell Signaling Technology, Lot #8
Mouse, monoclonal, anti-S6, clone 52D2, #2317, Cell Signaling Technology, Lot #13
Rabbit, monoclonal, anti-phospho-ULK1 (Ser757), clone D7O6U, #14202, Cell Signaling Technology, Lot #5
Rabbit, monoclonal, anti-ULK1, clone D8H5, #8054, Cell Signaling Technology, Lot #7
Rabbit, monoclonal, anti-phospho-GRB10 (Ser476), clone D4E6, #11817, Cell Signaling Technology, Lot #1
Rabbit, polyclonal, anti-GRB10, #23591-1-AP, Proteintech, Lot #00018824
Rabbit, monoclonal, anti-mTOR, clone 7C10, #2983, Cell Signaling Technology, Lot #19
Rabbit, monoclonal, anti-LC3B, clone D11, #3868, Cell Signaling Technology, Lot #14
Rabbit, polyclonal, anti-p62/SQSTM, #PM045, MBL
Rabbit, monoclonal, anti-TAX1BP1, clone D1D5, #5105, Cell Signaling Technology, Lot #2
Mouse, monoclonal, anti-NBR1, clone 4BR, #sc-130380, Santa Cruz Biotechnology, Lot #K1021
Rabbit, polyclonal, anti-TSC2, #4308, Cell Signaling Technology, Lot #6
Rabbit, polyclonal, anti-phospho-TSC2 (Thr1462), #3611, Cell Signaling Technology, Lot #6
Rabbit, polyclonal, anti-phospho-ACC1 (Ser79), #3661, Cell Signaling Technology, Lot #10
Rabbit, monoclonal, anti-MIOS, clone D12C6, #13557, discontinued, Cell Signaling Technology, Lot #1
Rabbit, monoclonal, anti-RAPTOR, clone 24C12, #2280, Cell Signaling Technology, Lot #13
Rabbit, monoclonal, anti-RagA, clone D8B5, #4357, Cell Signaling Technology, Lot #3
Rabbit, monoclonal, anti-RagC, clone D8H5, #9480, Cell Signaling Technology, Lot #2
Rabbit, monoclonal, anti-RagC, clone D31G9, #5466, discontinued, Cell Signaling Technology, Lot #4
Rabbit, polyclonal, anti-RagC, #3360, Cell Signaling Technology, Lot #2
Rabbit, polyclonal, anti-RagD, #4470, Cell Signaling Technology, Lot #2
Rabbit, polyclonal, anti-Cathepsin D, #2284, Cell Signaling Technology, Lot #2
Rat, monoclonal, anti-HA, clone 3F10, #11867423001, Roche
Rabbit, polyclonal, anti-DYKDDDDK Tag, #2368, Cell Signaling Technology, Lot #12
Rabbit, monoclonal, anti-GAPDH, clone 14C10, #2118, Cell Signaling Technology, Lot #16
Mouse, monoclonal, anti-Alpha-Tubulin, clone DM1A, #T9026, Sigma
Mouse, monoclonal, anti-Actin, clone C4, #612656, BD Transduction Laboratories, Lot #2182968
Mouse, monoclonal, anti-GRASP55/GORASP2, clone 1C9A3, #66627-1-Ig, Proteintech, Lot #10006147
Rabbit, polyclonal, anti-phospho-GRASP55/GORASP2 (Thr264), custom-made, Cell Signaling Technology
Rabbit, polyclonal, anti-M6P, #AG949, ABCD Antibodies
Rat, monoclonal, anti-LAMP2, clone ABL-93, Developmental Studies Hybridoma Bank
Mouse, monoclonal, anti-LAMP2, clone H4B4, Developmental Studies Hybridoma Bank
Mouse, monoclonal, anti-GM130, clone 35, #610822, BD Transduction Laboratories, Lot #0072108

Secondary antibodies
Peroxidase-conjugated AffiniPure donkey anti-rabbit IgG (H+L), polyclonal, #711-035-152, Jackson ImmunoResearch
Peroxidase-conjugated AffiniPure donkey anti-mouse IgG (H+L), polyclonal, #715-035-151, Jackson ImmunoResearch
Peroxidase-conjugated AffiniPure donkey anti-rat IgG (H+L), polyclonal, #712-035-153, Jackson ImmunoResearch
Rhodamine (TRITC)-conjugated AffiniPure donkey anti-mouse IgG (H+L), polyclonal, #715-025-150, Jackson ImmunoResearch
Alexa Fluor 488-conjugated AffiniPure donkey anti-rabbit IgG (H+L), polyclonal, #711-545-152, Jackson ImmunoResearch
Alexa Fluor 594-conjugated AffiniPure donkey anti-mouse IgG (H+L) polyclonal, #715-585-150, Jackson ImmunoResearch

A list of all primary antibodies used in this study is also provided in Suppl. Table 1.

Validation

Specificity of phospho-antibodies extensively verified in this study and in the context of other projects in the Demetriades lab, using inhibitors for the respective kinases or starvation media.
Specificity of total protein antibodies extensively verified in this study and in the context of other projects in the Demetriades lab, using knock-out cell lines or knock-down and overexpression experiments.

Additional information for all primary antibodies used in this study (aalso in Suppl. Table 1) can be found in the manufacturer's website for each product:

anti-phospho-S6K (Thr389), #97596, Cell Signaling Technology, validated for Western Blotting, relevant citations can be found on the manufacturer's website (https://www.cellsignal.com/products/primary-antibodies/phospho-p70-s6-kinase-thr389-d5u1o-rabbit-mab/97596)

anti-S6K, #9202, Cell Signaling Technology, validated for Western Blotting and Immunoprecipitation, relevant citations can be found on the manufacturer's website (https://www.cellsignal.com/products/primary-antibodies/p70-s6-kinase-antibody/9202)

anti-phospho-TFEB (Ser211), #37681, Cell Signaling Technology, validated for Western Blotting, relevant citations can be found on

the manufacturer's website (https://www.cellsignal.com/products/primary-antibodies/phospho-tfeb-ser211-e9s8n-rabbit-mab/37681)

anti-TFEB, #4240, Cell Signaling Technology, validated for Western Blotting and Immunoprecipitation, relevant citations can be found on the manufacturer's website (https://www.cellsignal.com/products/primary-antibodies/tfeb-antibody/4240)

anti-TFEB, #A303-673A, Bethyl Laboratories, validated for Western Blotting and Immunoprecipitation, relevant citations can be found on the manufacturer's website (https://www.biomol.com/products/antibodies/primary-antibodies/general/anti-tfeb-a303-673a-t?number=A303-673A)

anti-TFE3, #14779, discontinued, Cell Signaling Technology, validated for Western Blotting, relevant citations can be found on the manufacturer's website (https://www.cellsignal.com/products/primary-antibodies/tfe3-antibody/14779)

anti-phospho-AKT (Ser473), #9271, Cell Signaling Technology, validated for Western Blotting, Immunoprecipitation, Immunofluorescence and Flow Cytometry, relevant citations can be found on the manufacturer's website (https://www.cellsignal.com/products/primary-antibodies/phospho-akt-ser473-antibody/9271)

anti-AKT, #9272, Cell Signaling Technology, validated for Western Blotting, Immunoprecipitation, Immunofluorescence and Flow Cytometry, relevant citations can be found on the manufacturer's website (https://www.cellsignal.com/products/primary-antibodies/akt-antibody/9272)

anti-phospho-4E-BP1 (Ser65), #13443, Cell Signaling Technology, validated for Western Blotting and Immunoprecipitation, relevant citations can be found on the manufacturer's website (https://www.cellsignal.com/products/primary-antibodies/phospho-4e-bp1-ser65-d9g1q-rabbit-mab/13443)

anti-phospho-4E-BP1 (Thr37/46), #9459, Cell Signaling Technology, validated for Western Blotting, relevant citations can be found on the manufacturer's website (https://www.cellsignal.com/products/primary-antibodies/phospho-4e-bp1-thr37-46-antibody/9459)

anti-4E-BP1, #9452, Cell Signaling Technology, validated for Western Blotting and Immunoprecipitation, relevant citations can be found on the manufacturer's website (https://www.cellsignal.com/products/primary-antibodies/4e-bp1-antibody/9452)

anti-phospho-S6 (Ser240/244), #5364, Cell Signaling Technology, validated for Western Blotting, Immunohistochemistry, Immunofluorescence and Flow Cytometry, relevant citations can be found on the manufacturer's website (https://www.cellsignal.com/products/primary-antibodies/phospho-s6-ribosomal-protein-ser240-244-d68f8-xp-rabbit-mab/5364)

anti-S6, #2317, Cell Signaling Technology, validated for Western Blotting, Immunohistochemistry, Immunofluorescence and Flow Cytometry, relevant citations can be found on the manufacturer's website (https://www.cellsignal.com/products/primary-antibodies/s6-ribosomal-protein-54d2-mouse-mab/2317)

anti-phospho-ULK1 (Ser757), #14202, Cell Signaling Technology, validated for Western Blotting, Immunoprecipitation, Immunofluorescence and Flow Cytometry, relevant citations can be found on the manufacturer's website (https://www.cellsignal.com/products/primary-antibodies/phospho-ulk1-ser757-d7o6u-rabbit-mab/14202)

anti-ULK1, #8054, Cell Signaling Technology, validated for Western Blotting and Immunoprecipitation, relevant citations can be found on the manufacturer's website (https://www.cellsignal.com/products/primary-antibodies/ulk1-d8h5-rabbit-mab/8054)

anti-phospho-GRB10 (Ser476), #11817, Cell Signaling Technology, validated for Western Blotting and Immunoprecipitation, relevant citations can be found on the manufacturer's website (https://www.cellsignal.com/products/primary-antibodies/phospho-grb10-ser476-d4e6-rabbit-mab/11817)

anti-GRB10, #23591-1-AP, Proteintech, validated for Western Blotting, Immunohistochemistry and Immunofluorescence, relevant citations can be found on the manufacturer's website (https://www.ptglab.com/products/GRB10-Antibody-23591-1-AP.htm)

anti-mTOR, #2983, Cell Signaling Technology, validated for Western Blotting, Immunoprecipitation, Immunohistochemistry, Immunofluorescence and Flow Cytometry, relevant citations can be found on the manufacturerr's website (https://www.cellsignal.com/products/primary-antibodies/mtor-7c10-rabbit-mab/2983)

anti-LC3B, #3868, Cell Signaling Technology, validated for Western Blotting, Immunofluorescence and Flow Cytometry, relevant citations can be found on the manufacturer's website (https://www.cellsignal.com/products/primary-antibodies/lc3b-d11-xp-rabbit-mab/3868)

anti-p62, #PM045, MBL, validated for Western Blotting, Immunocytochemistry, Immunohistochemistry and Immunoprecipitation, relevant citations can be found on the manufacturer's website (https://products.mblintl.com/products/pm045/)

anti-TAX1BP1, #5105, Cell Signaling Technology, validated for Western Blotting and Immunoprecipitation, relevant citations can be found on the manufacturer's website (https://www.cellsignal.com/products/primary-antibodies/tax1bp1-d1d5-rabbit-mab/5105)

anti-NBR1, #sc-130380, Santa Cruz Biotechnology, validated for Western Blotting and Immunoprecipitation, relevant citations can be found on the manufacturer's website (https://www.scbt.com/p/nbr1-antibody-4br)

anti-TSC2, #4308, Cell Signaling Technology, validated for Western Blotting, Immunoprecipitation, Immunofluorescence and Flow Cytometry, relevant citations can be found on the manufacturer's website (https://www.cellsignal.com/products/primary-antibodies/tuberin-tsc2-d93f12-xp-rabbit-mab/4308)

anti-phospho-TSC2 (Ser1462), #3611, Cell Signaling Technology, validated for Western Blotting, relevant citations can be found on the manufacturer's website (https://www.cellsignal.com/products/primary-antibodies/phospho-tuberin-tsc2-thr1462-antibody/3611)

anti-phospho-ACC1 (Ser79), #3661, Cell Signaling Technology, validated for Western Blotting, Immunoprecipitation and Immunohistochemistry, relevant citations can be found on the manufacturer's website (https://www.cellsignal.com/products/primary-antibodies/phospho-acetyl-coa-carboxylase-ser79-antibody/3661)

anti-Mios, #13557, discontinued, Cell Signaling Technology, validated for Western Blotting and Immunoprecipitation, relevant citations can be found on the manufacturer's website (https://www.cellsignal.com/products/primary-antibodies/mios-d12c6-rabbit-mab/13557)

anti-RAPTOR, #2280, Cell Signaling Technology, validated for Western Blotting and Immunoprecipitation, relevant citations can be found on the manufacturer's website (https://www.cellsignal.com/products/primary-antibodies/raptor-24c12-rabbit-mab/2280)

anti-RagA, #4357, Cell Signaling Technology, validated for Western Blotting and Immunoprecipitation, relevant citations can be found on the manufacturer's website (https://www.cellsignal.com/products/primary-antibodies/raga-d8b5-rabbit-mab/4357)

anti-RagC, #9480, Cell Signaling Technology, validated for Western Blotting, Immunoprecipitation, Immunofluorescence and Flow Cytometry, relevant citations can be found on the manufacturer's website (https://www.cellsignal.com/products/primary-antibodies/ragc-d8h5-rabbit-mab/9480)

anti-RagC, #5466, discontinued, Cell Signaling Technology, validated for Western Blotting, Immunoprecipitation, and Immunohistochemistry, relevant citations can be found on the manufacturer's website (https://www.cellsignal.com/products/primary-antibodies/ragc-d31g9-xp-rabbit-mab/5466)

anti-RagC, #3360, Cell Signaling Technology, validated for Western Blotting, Immunoprecipitation and Immunofluorescence, relevant citations can be found on the manufacturer's website (https://www.cellsignal.com/products/primary-antibodies/ragc-antibody/3360)

anti-RagD, #4470, Cell Signaling Technology, validated for Western Blotting, relevant citations can be found on the manufacturer's website (https://www.cellsignal.com/products/primary-antibodies/ragd-antibody/4470)

anti-Cathepsin D, #2284, Cell Signaling Technology, validated for Western Blotting, relevant citations can be found on the manufacturer's website (https://www.cellsignal.com/products/primary-antibodies/cathepsin-d-antibody/2284)

anti-HA, #11867423001, Roche, validated for Western Blotting, Dot Blots, ELISA, Immunocytochemistry and Immunoprecipitation, relevant citations can be found on the manufacturer's website (https://www.sigmaaldrich.com/DE/en/product/roche/roahaha)

anti-DYKDDDDK Tag, #2368, Cell Signaling Technology, validated for Western Blotting, Immunoprecipitation and Flow Cytometry, relevant citations can be found on the manufacturer's website (https://www.cellsignal.com/products/primary-antibodies/dykddddk-tag-antibody-binds-to-same-epitope-as-sigma-s-anti-flag-m2-antibody/2368)

anti-GAPDH, #2118, Cell Signaling Technology, validated for Western Blotting, Immunohistochemistry, Immunofluorescence and Flow Cytometry, relevant citations can be found on the manufacturer's website (https://www.cellsignal.com/products/primary-antibodies/gapdh-14c10-rabbit-mab/2118)

anti-alpha-tubulin, #T9026, Sigma, validated for Western Blotting and Immunofluorescence, relevant citations can be found on the manufacturer's website (https://www.sigmaaldrich.com/DE/en/product/sigma/t9026)

anti-Actin, #612656, BD Trandusction Laboratories, validated for Western Blotting and Immunofluorescence, relevant citations can be found on the manufacturer's website (https://www.bdbiosciences.com/en-ca/products/reagents/microscopy-imaging-reagents/immunofluorescence-reagents/purified-mouse-anti-actin-ab-5.612657)

anti-GRASP55/GORASP2, #66627-1-Ig, Proteintech, validated for Western Blotting, Immunohistochemistry, Immunofluorescence and ELISA, relevant citations can be found on the manufacturer's website (https://www.ptglab.com/de/products/GORASP2-Antibody-66627-1-Ig.htm)

anti-phospho-GRASP55/GORASP2 (Thr264), custom-made, Cell Signaling Technology, validated for Western Blotting in this manuscript, Extended Data Figure 5 a-b.

anti-M6P, #AG949, ABCD antibodies, validated for Western Blotting, relevant citations can be found on the manufacturer's website (https://abcd-antibodies.com/products/anti-m6p-antibody)

anti-LAMP2, clone ABL93, Developmental Studies Hybridoma Bank, validated for Western Blotting, FACS, Immunofluorescence, Immunohistochemistry and Immunoprecipitation, relevant citations can be found on the manufacturer's website (https://dshb.biology.uiowa.edu/ABL-93)

anti-LAMP2, clone H4B4, Developmental Studies Hybridoma Bank, validated for Western Blotting, FACS, Function Blocking, Immunofluorescence, Immunohistochemistry and Immunoprecipitation, relevant citations can be found on the manufacturer's website (https://dshb.biology.uiowa.edu/H4B4)

anti-GM130, #610822, BD Trandusction Laboratories, validated for Western Blotting, Immunofluorescence and Immunohistochemistry, relevant citations can be found on the manufacturer's website (https://www.bdbiosciences.com/en-us/products/reagents/microscopy-imaging-reagents/immunofluorescence-reagents/purified-mouse-anti-gm130.610822)

Eukaryotic cell lines

Policy information about cell lines

Cell line source(s)	The parental HEK293FT cells were purchased from Invitrogen before the initiation of the project. Wild-type and RagA/B KO immortalized MEFs were a kind gift of Kun-Liang Guan (described in PMID: 25567907). SW-620 cells were obtained from ATCC (#CCL-227). WI-26 cells (ATCC, #CCL-95.1) are part of the Demetriades lab collection, originally obtained from the Institute for Biochemistry of the University of Cologne.
Authentication	The identity of the HEK293FT cells was validated by the Multiplex human Cell Line Authentication test (Multiplexion GmbH), which uses a single nucleotide polymorphism (SNP) typing approach, and was performed as described at www.multiplexion.de. The identity of the WI-26 cells was validated using the Short Tandem Repeat (STR) profiling service, provided by Multiplexion GmbH. The other cell lines (MEFs, SW-620) were not authenticated in the context of this study.
Mycoplasma contamination	All cell lines were regularly tested for Mycoplasma contamination, using a PCR-based approach and were confirmed to be Mycoplasma-free.
Commonly misidentified lines (See ICLAC register)	No commonly misidentified cell lines were used in this study.

Flow Cytometry

Plots

Confirm that:

☒ The axis labels state the marker and fluorochrome used (e.g. CD4-FITC).

☒ The axis scales are clearly visible. Include numbers along axes only for bottom left plot of group (a 'group' is an analysis of identical markers).

☒ All plots are contour plots with outliers or pseudocolor plots.

☒ A numerical value for number of cells or percentage (with statistics) is provided.

Methodology

Sample preparation	See 'OPP assay' part in the Methods section
Instrument	BD LSR Fortessa (TM) Cell Analyzer flow cytometer (BD Biosciences)
Software	FlowJo (TM) v10 software (TreeStar)
Cell population abundance	10 000 cells were acquired and around 90% were analysed after gating.
Gating strategy	Intact cells

☒ Tick this box to confirm that a figure exemplifying the gating strategy is provided in the Supplementary Information.
