# Supplementary figures and images for: Spatial and functional separation of mTORC1 signalling in response to different amino acid sources
Source: Nat Cell Biol. 2024 Oct 9;26(11):1918–33. doi: 10.1038/s41556-024-01523-7 (PMC11567901; doi:10.1038/s41556-024-01523-7)

## Uncropped blots for Fig. 1g

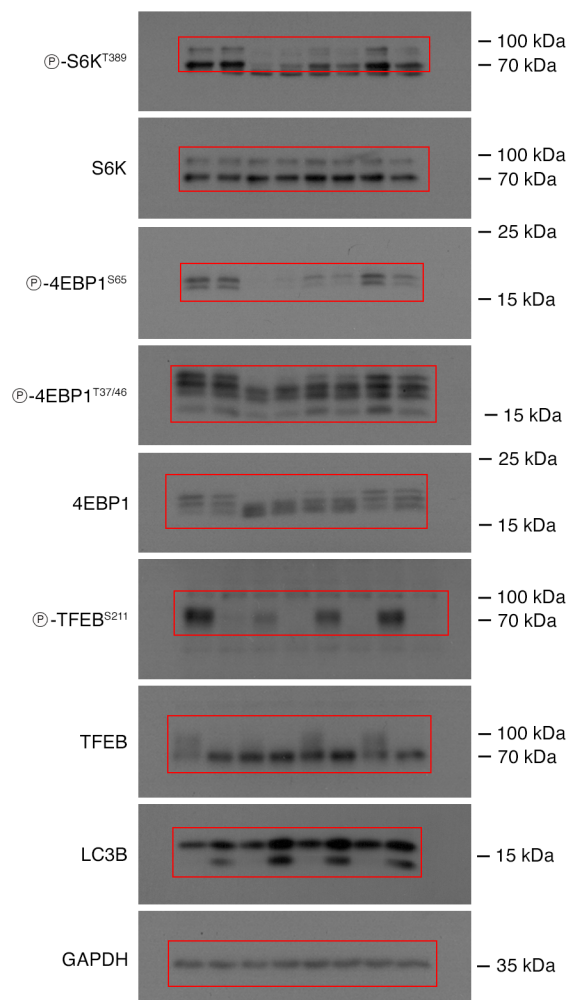

Supplement: Supplementary file 3 — Unprocessed western blots for Fig. 1. [file 41556_2024_1523_MOESM3_ESM.pdf]

## Uncropped blots for Fig. 2d

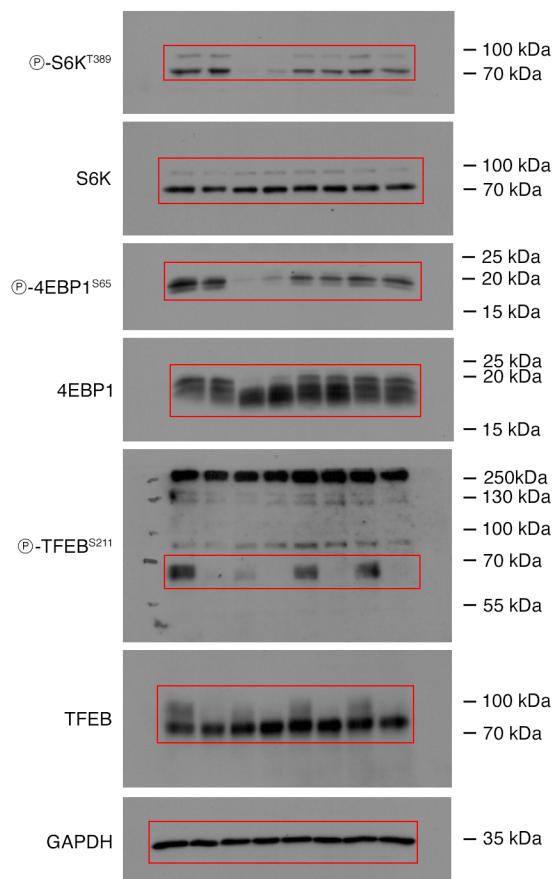

Supplement: Supplementary file 4 — Unprocessed western blots for Fig. 2. [file 41556_2024_1523_MOESM4_ESM.pdf]

### Uncropped blots for Fig. 3d

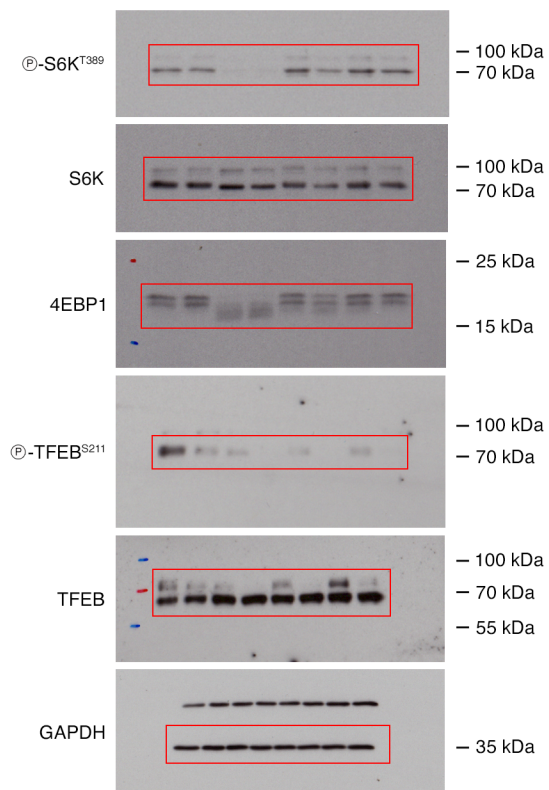

# Uncropped blots for Fig. 3e

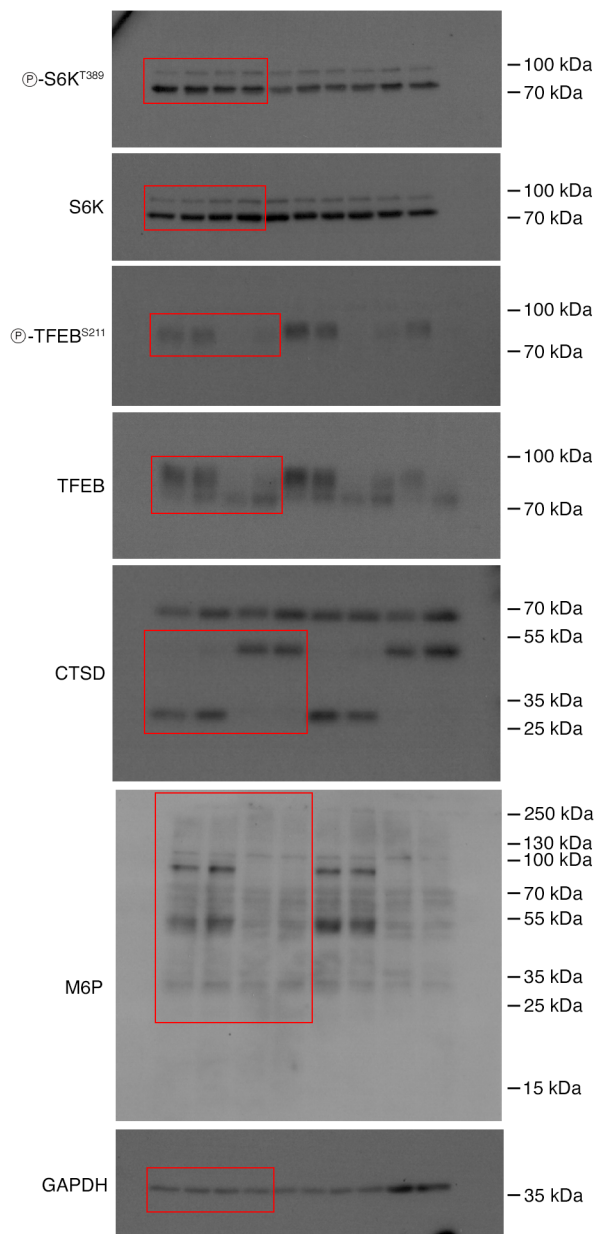

Uncropped blots for Fig. 3f

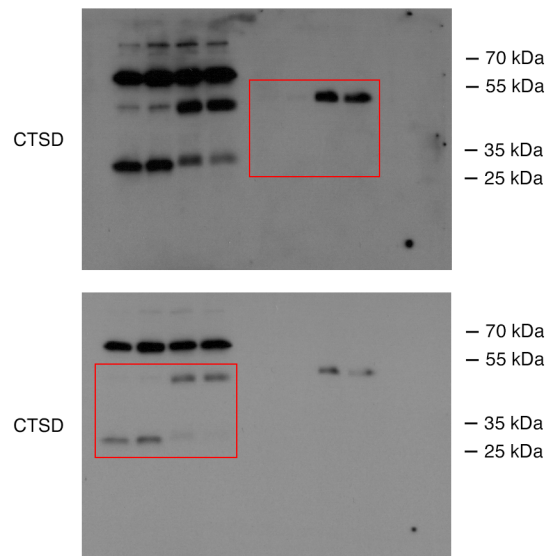

Supplement: Supplementary file 5 — Unprocessed western blots for Fig. 3. [file 41556_2024_1523_MOESM5_ESM.pdf]

## Uncropped blots for Fig. 4c

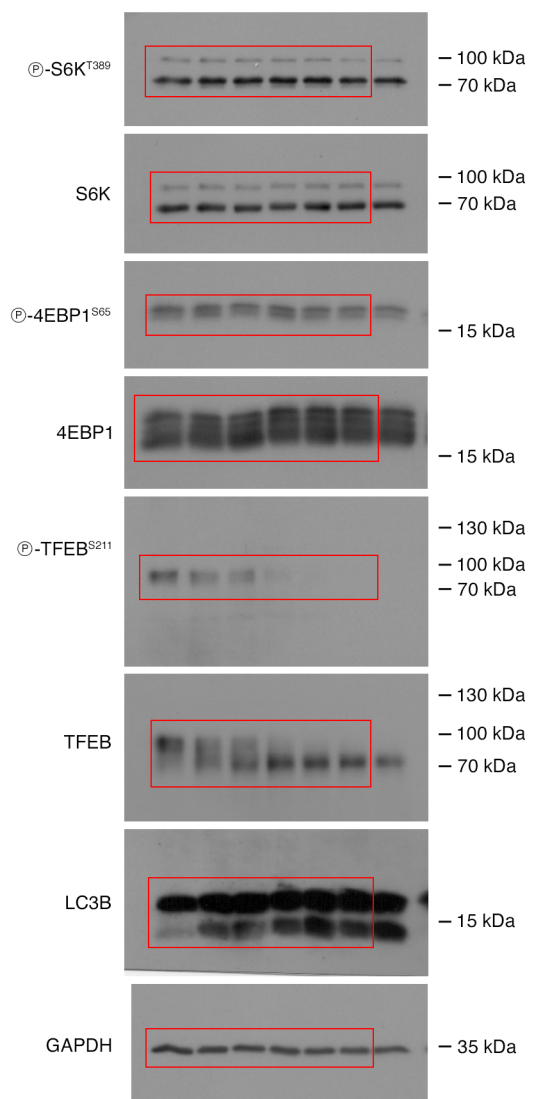

# Uncropped blots for Fig. 4f

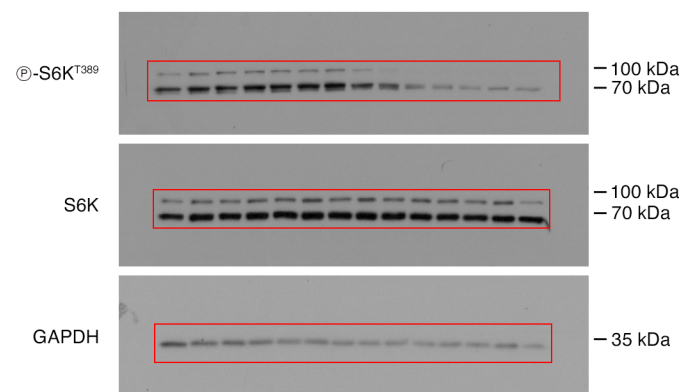

Supplement: Supplementary file 6 — Unprocessed western blots for Fig. 4. [file 41556_2024_1523_MOESM6_ESM.pdf]

Uncropped blots for Fig. 5d

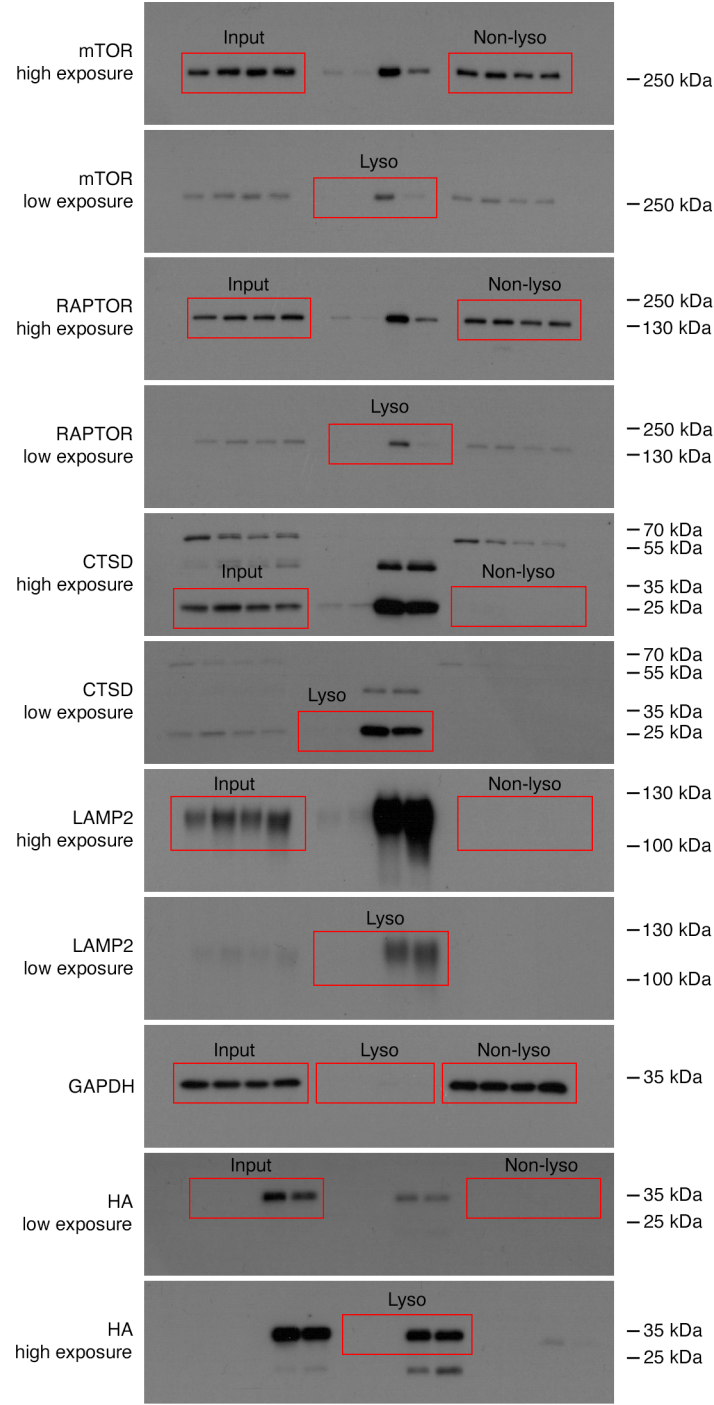

Supplement: Supplementary file 7 — Unprocessed western blots for Fig. 5. [file 41556_2024_1523_MOESM7_ESM.pdf]

**Uncropped blots for Fig. 7a**

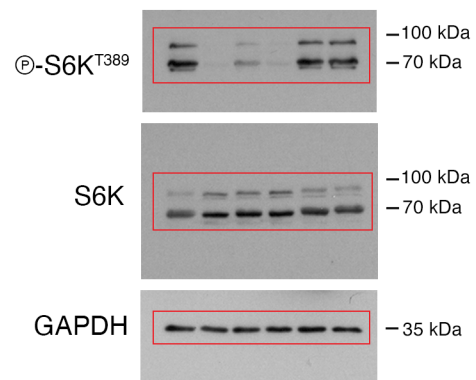

# Uncropped blots for Fig. 7c

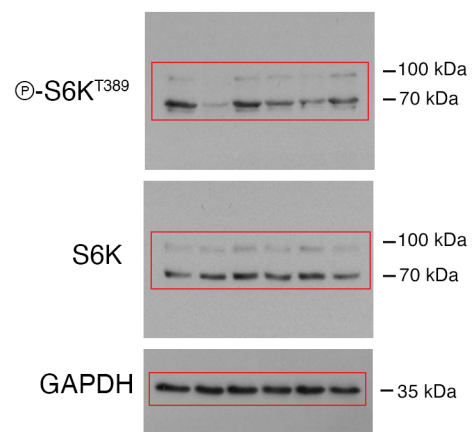

**Uncropped blots for Fig. 7e**

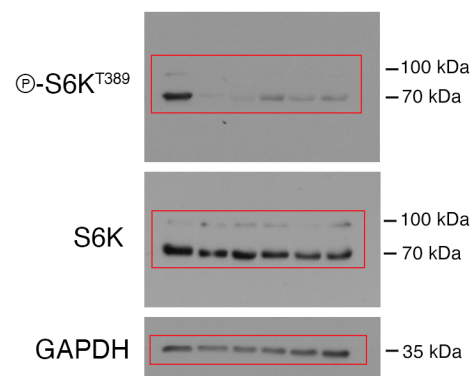

Supplement: Supplementary file 9 — Unprocessed western blots for Fig. 7. [file 41556_2024_1523_MOESM9_ESM.pdf]

# Uncropped blots for Extended Data Fig. 1a

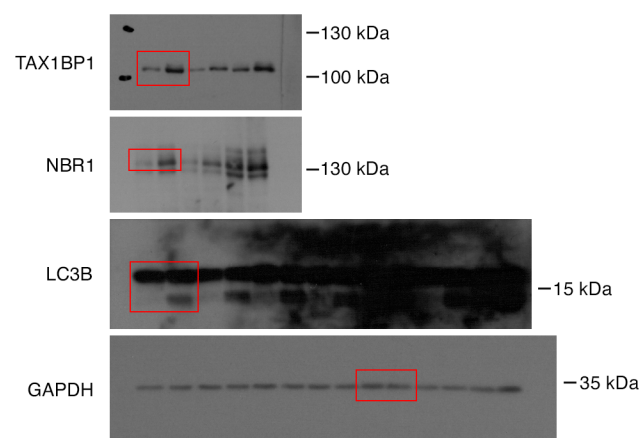

Supplement: Supplementary file 10 — Unprocessed western blots for Extended Data Fig. 1. [file 41556_2024_1523_MOESM10_ESM.pdf]

## Uncropped blots for Extended Data Fig. 2c

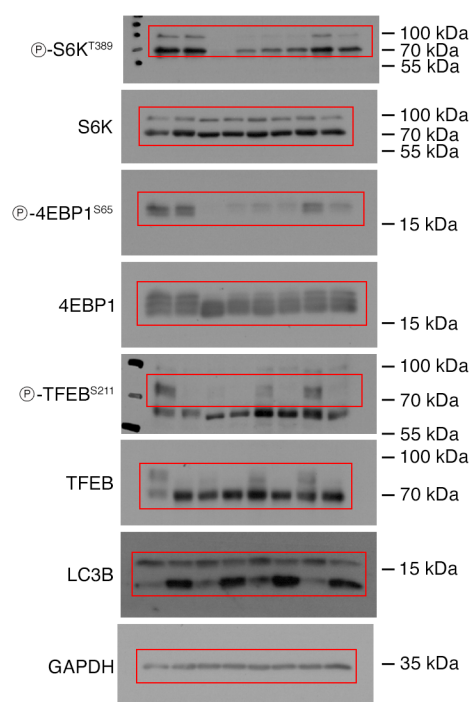

## Uncropped blots for Extended Data Fig. 2f

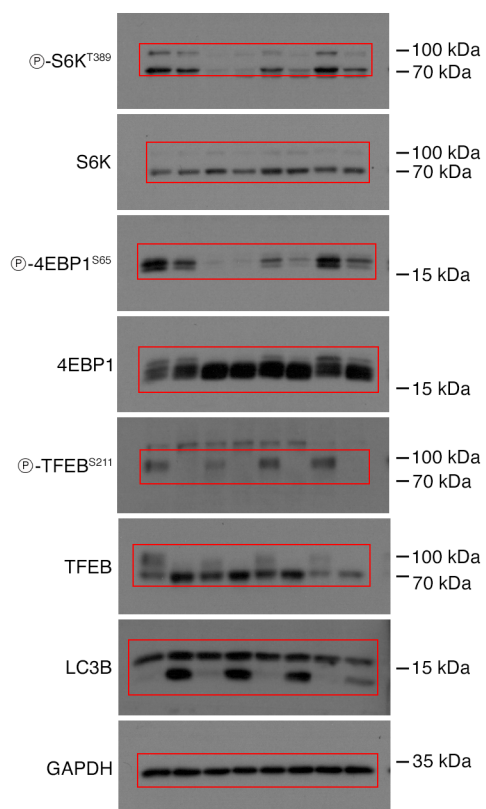

Supplement: Supplementary file 11 — Unprocessed western blots for Extended Data Fig. 2. [file 41556_2024_1523_MOESM11_ESM.pdf]

## Uncropped blots for Extended Data Fig. 6e

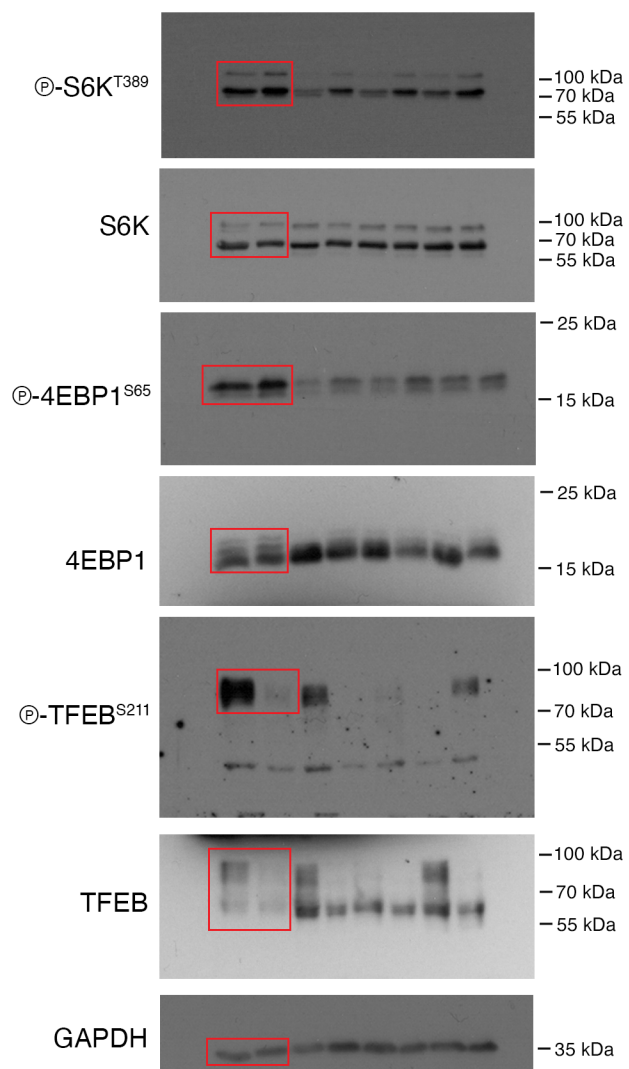

Supplement: Supplementary file 15 — Unprocessed western blots for Extended Data Fig. 6. [file 41556_2024_1523_MOESM15_ESM.pdf]

Uncropped blots for Extended Data Fig. 8d

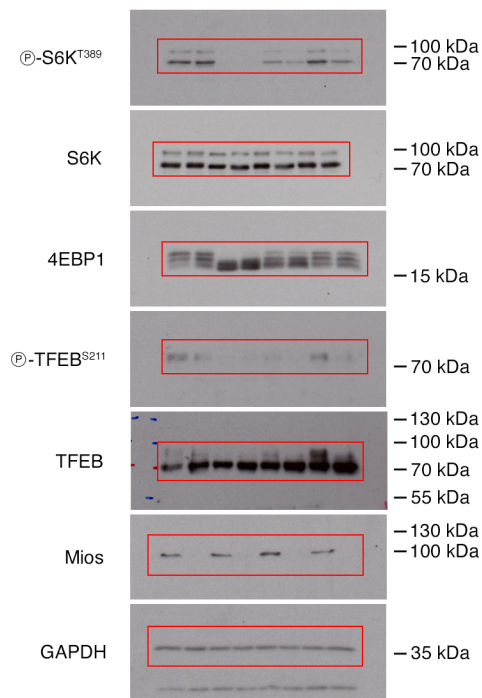

Supplement: Supplementary file 17 — Unprocessed western blots for Extended Data Fig. 8. [file 41556_2024_1523_MOESM17_ESM.pdf]

# Uncropped blots for Extended Data Fig. 9c

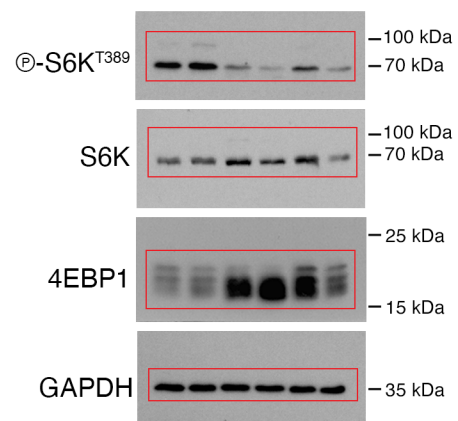

## Uncropped blots for Extended Data Fig. 9e

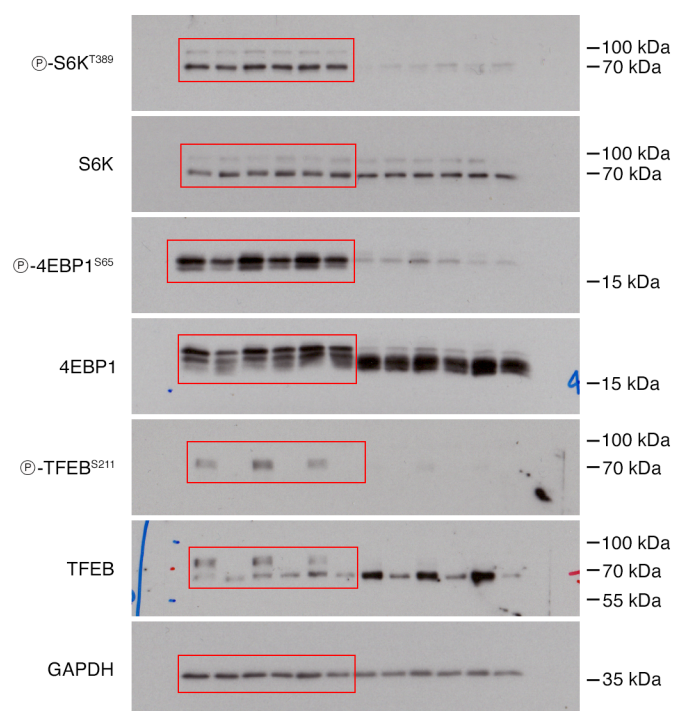

Supplement: Supplementary file 18 — Unprocessed western blots for Extended Data Fig. 9. [file 41556_2024_1523_MOESM18_ESM.pdf]
