# Supplementary material for: Spatial and functional separation of mTORC1 signalling in response to different amino acid sources
Source: Nat Cell Biol. 2024 Oct 9;26(11):1918–33. doi: 10.1038/s41556-024-01523-7 (PMC11567901; doi:10.1038/s41556-024-01523-7)

Uncropped blots for Fig. 6a

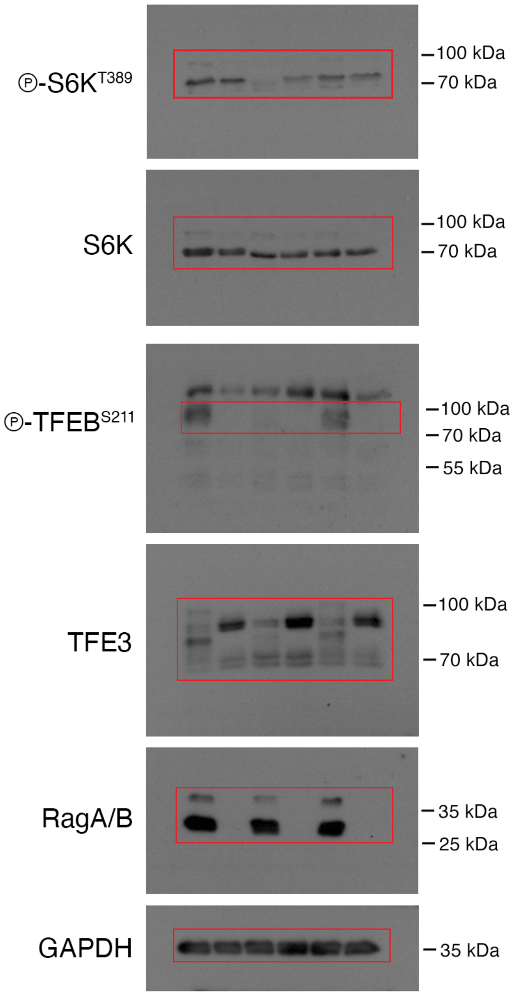

Uncropped blots for Fig. 6b

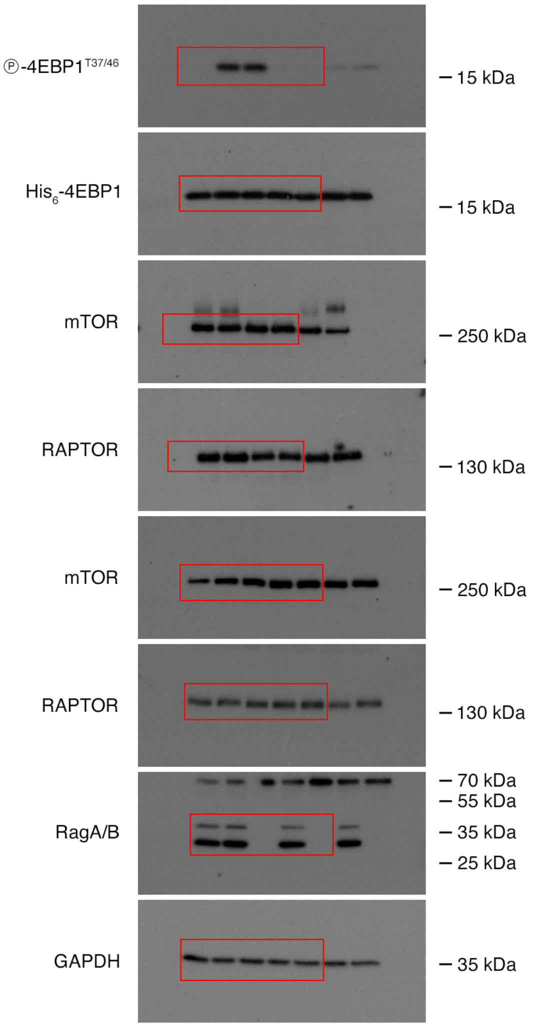

Uncropped blots for Fig. 6c

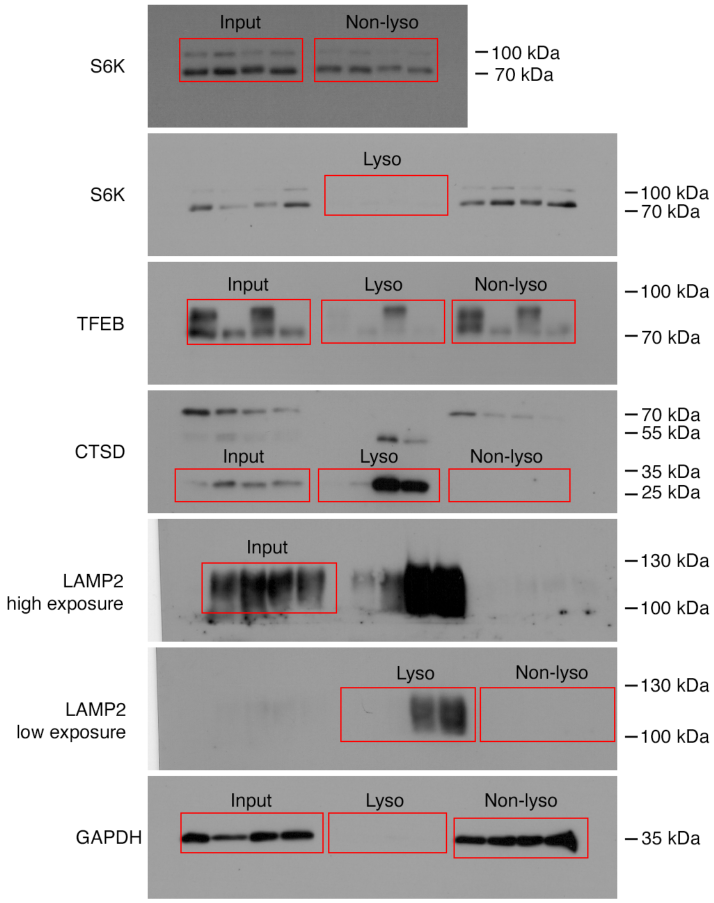

Uncropped blots for Fig. 6d

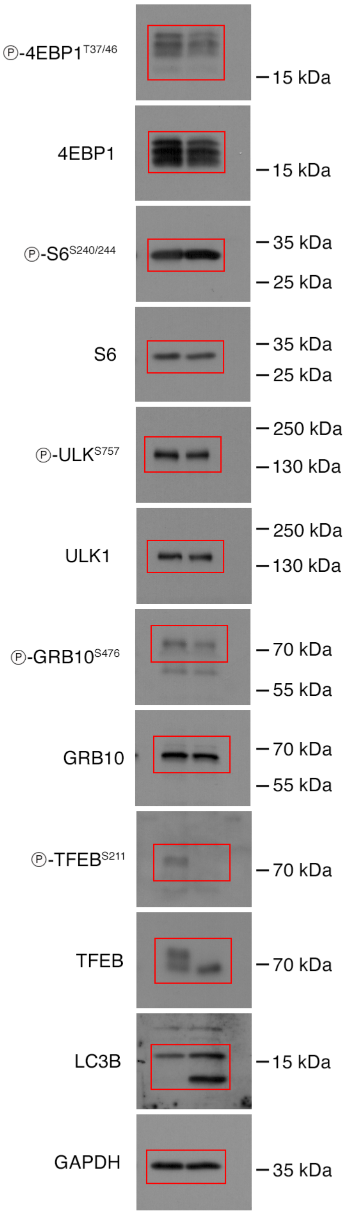

Uncropped blots for Fig. 6e

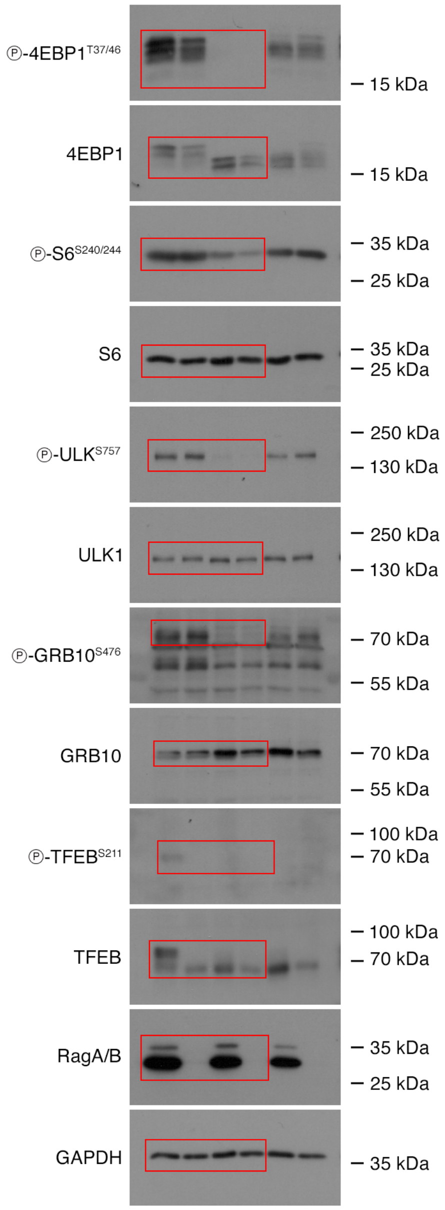

Uncropped blots for Fig. 6f

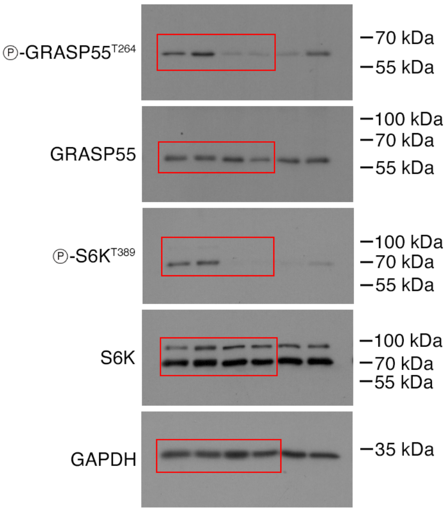

Uncropped blots for Fig. 6g

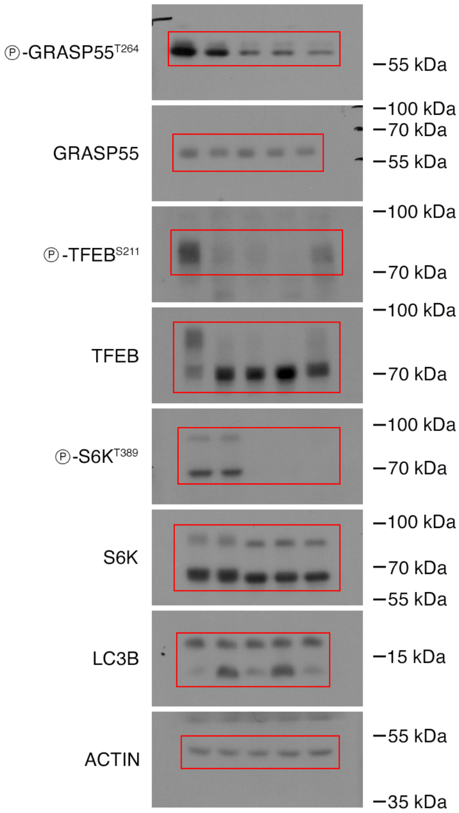

Uncropped blots for Fig. 6h

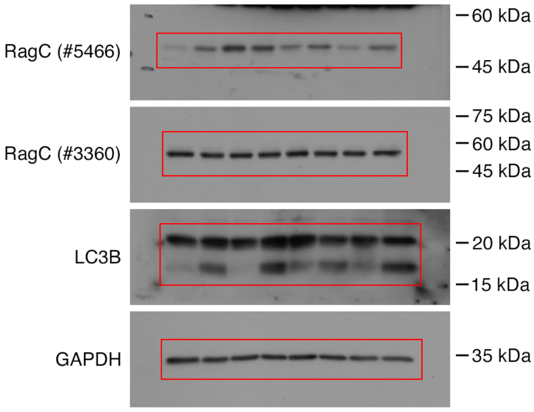

Supplement: Supplementary file 8 — Unprocessed western blots for Fig. 6. [file 41556_2024_1523_MOESM8_ESM.pdf]
