# Supplementary material for: Spatial and functional separation of mTORC1 signalling in response to different amino acid sources
Source: Nat Cell Biol. 2024 Oct 9;26(11):1918–33. doi: 10.1038/s41556-024-01523-7 (PMC11567901; doi:10.1038/s41556-024-01523-7)

Uncropped blots for Extended Data Fig. 3c

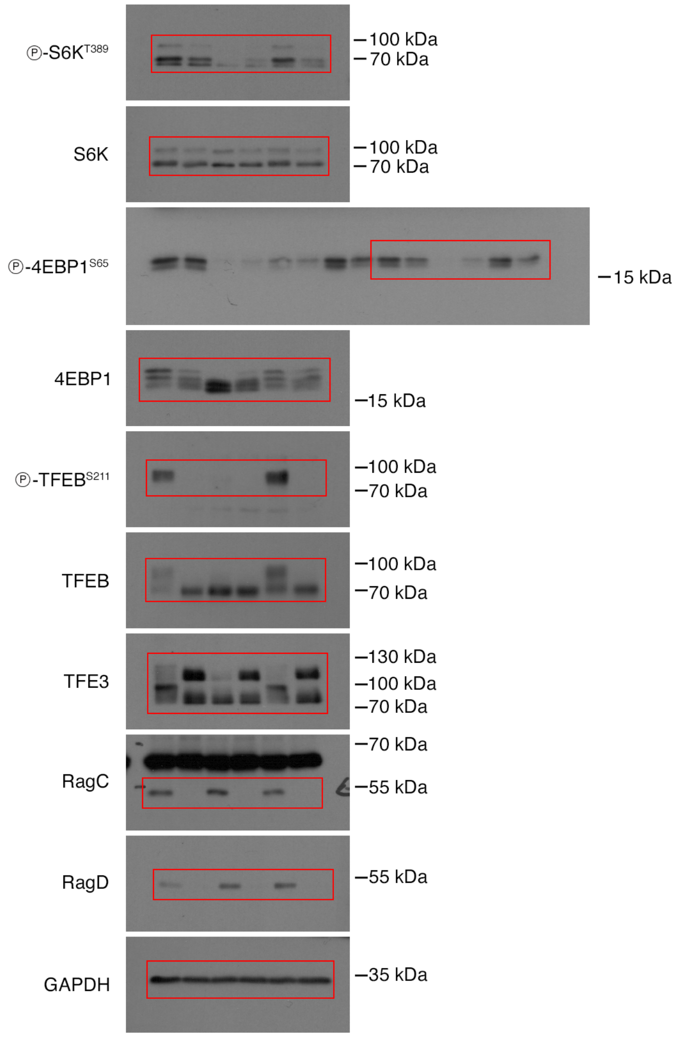

Uncropped blots for Extended Data Fig. 3f

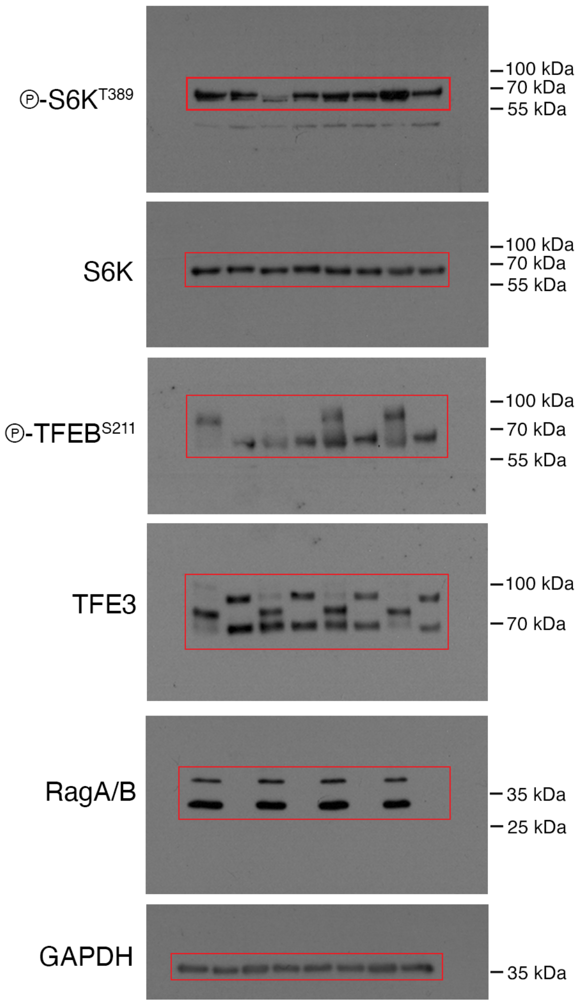

Uncropped blots for Extended Data Fig. 3g

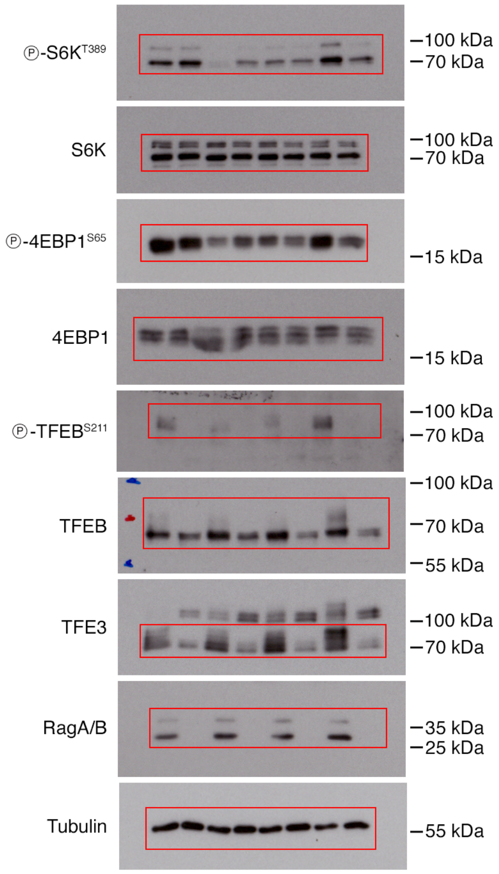

Supplement: Supplementary file 12 — Unprocessed western blots for Extended Data Fig. 3. [file 41556_2024_1523_MOESM12_ESM.pdf]
