# Supplementary material for: Spatial and functional separation of mTORC1 signalling in response to different amino acid sources
Source: Nat Cell Biol. 2024 Oct 9;26(11):1918–33. doi: 10.1038/s41556-024-01523-7 (PMC11567901; doi:10.1038/s41556-024-01523-7)

Uncropped blots for Extended Data Fig. 5a

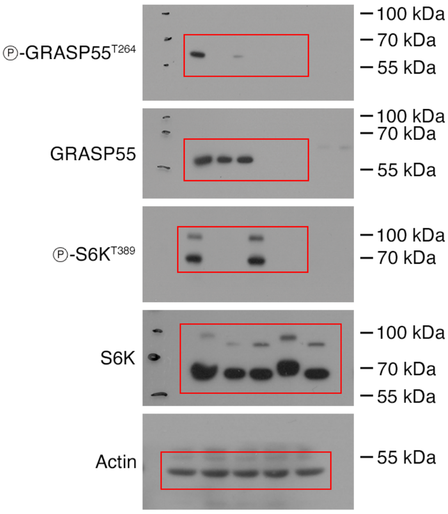

Uncropped blots for Extended Data Fig. 5b

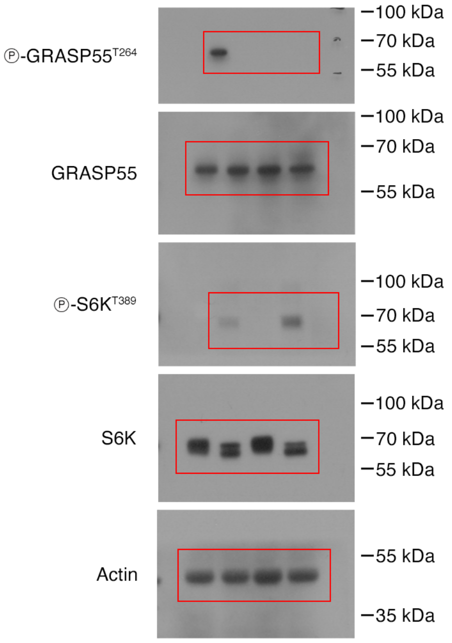

Uncropped blots for Extended Data Fig. 5c

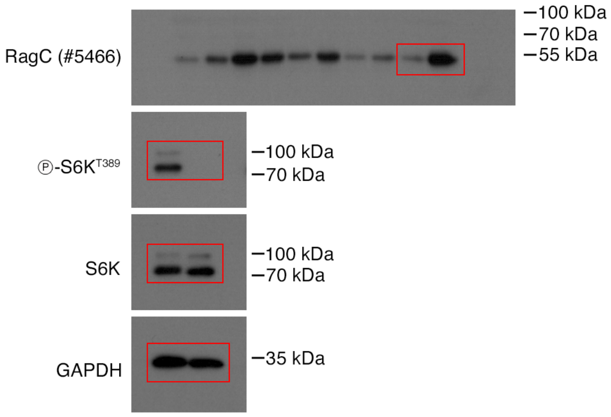

Supplement: Supplementary file 14 — Unprocessed western blots for Extended Data Fig. 5. [file 41556_2024_1523_MOESM14_ESM.pdf]
