# Supplementary material for: Spatial and functional separation of mTORC1 signalling in response to different amino acid sources
Source: Nat Cell Biol. 2024 Oct 9;26(11):1918–33. doi: 10.1038/s41556-024-01523-7 (PMC11567901; doi:10.1038/s41556-024-01523-7)

Uncropped blots for Extended Data Fig. 7a

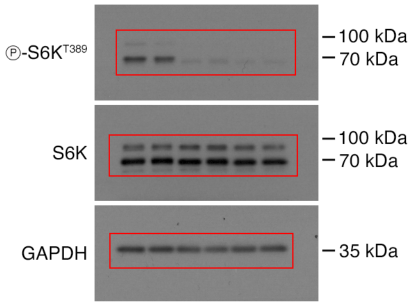

Uncropped blots for Extended Data Fig. 7b

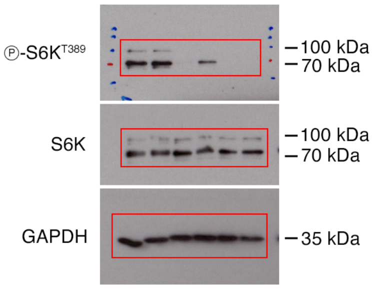

Uncropped blots for Extended Data Fig. 7c

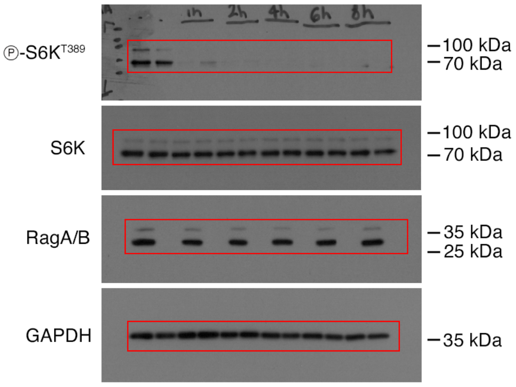

Uncropped blots for Extended Data Fig. 7d

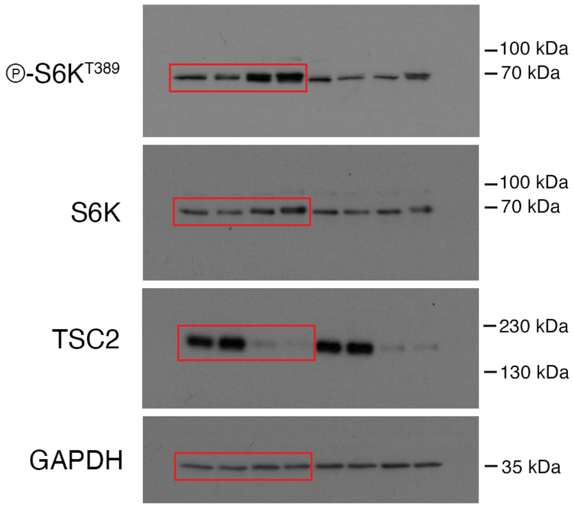

Uncropped blots for Extended Data Fig. 7e

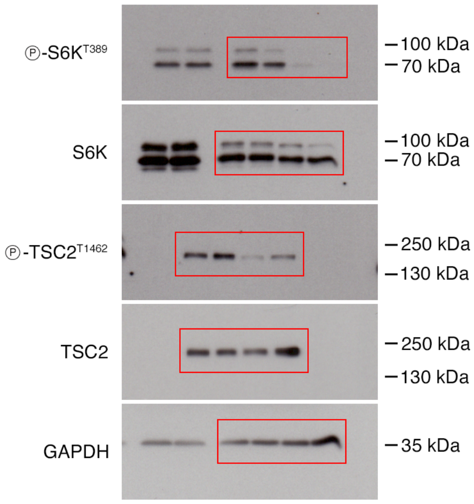

Uncropped blots for Extended Data Fig. 7f

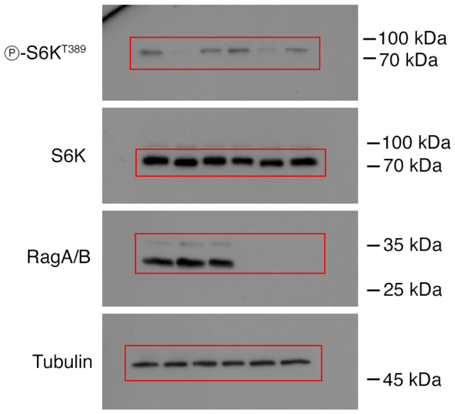

Uncropped blots for Extended Data Fig. 7g

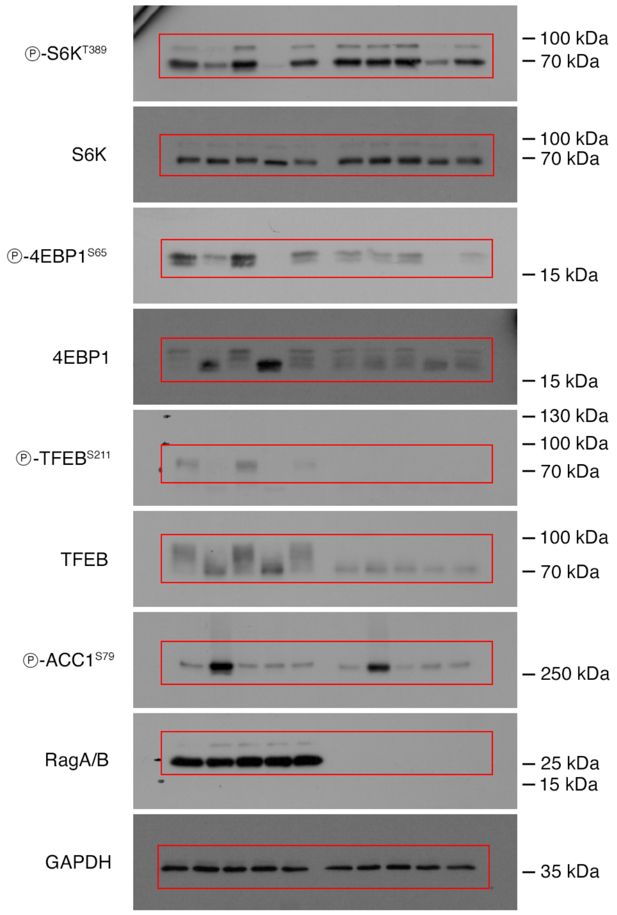

Supplement: Supplementary file 16 — Unprocessed western blots for Extended Data Fig. 7. [file 41556_2024_1523_MOESM16_ESM.pdf]
